# Macrocyclic FKBP51 Ligands Define a Transient Binding Mode with Enhanced Selectivity

**DOI:** 10.1002/anie.202017352

**Published:** 2021-05-07

**Authors:** Andreas M. Voll, Christian Meyners, Martha C. Taubert, Thomas Bajaj, Tim Heymann, Stephanie Merz, Anna Charalampidou, Jürgen Kolos, Patrick L. Purder, Thomas M. Geiger, Pablo Wessig, Nils C. Gassen, Andreas Bracher, Felix Hausch

**Affiliations:** ^1^ Department Chemistry and Biochemistry Clemens-Schöpf-Institute Technical University Darmstadt Alarich-Weiss Strasse 4 64287 Darmstadt Germany; ^2^ Research Group Neurohomeostasis Department of Psychiatry and Psychotherapy University of Bonn Venusberg Campus 1 53127 Bonn Germany; ^3^ Universität Potsdam Institut für Chemie Karl-Liebknecht-Strasse 24–25 14476 Potsdam Germany; ^4^ Max-Planck-Institute of Biochemistry Am Klopferspitz 18 82152 Martinsried Germany

**Keywords:** FKBP51, immunophilin, macrocycle, subtype selectivity, transient binding pocket

## Abstract

Subtype selectivity represents a challenge in many drug discovery campaigns. A typical example is the FK506 binding protein 51 (FKBP51), which has emerged as an attractive drug target. The most advanced FKBP51 ligands of the SAFit class are highly selective vs. FKBP52 but poorly discriminate against the homologs and off‐targets FKBP12 and FKBP12.6. During a macrocyclization pilot study, we observed that many of these macrocyclic analogs have unanticipated and unprecedented preference for FKBP51 over FKBP12 and FKBP12.6. Structural studies revealed that these macrocycles bind with a new binding mode featuring a transient conformation, which is disfavored for the small FKBPs. Using a conformation‐sensitive assay we show that this binding mode occurs in solution and is characteristic for this new class of compounds. The discovered macrocycles are non‐immunosuppressive, engage FKBP51 in cells, and block the cellular effect of FKBP51 on IKKα. Our findings provide a new chemical scaffold for improved FKBP51 ligands and the structural basis for enhanced selectivity.

## Graphical abstract

Macrocyclic FKBP51 ligands reveal a new transient binding pocket for the FK506‐binding protein 51 and enable the first ligands that also discriminate against the close homologs FKBP12 and FKBP12.6. This macrocyclic motif represents a new scaffold to develop drugs for depression, obesity or chronic pain.

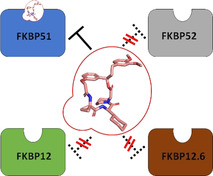

## Introduction

Proteins often cluster in families with similar structure. The discovery of selective ligands that can discriminate between these close homologs remains a formidable challenge in chemical biology as well as in drug development. Most proteins are flexible and differential dynamics have been suggested as a way to distinguish between otherwise very similar proteins.^[1]^

**Figure 1:**
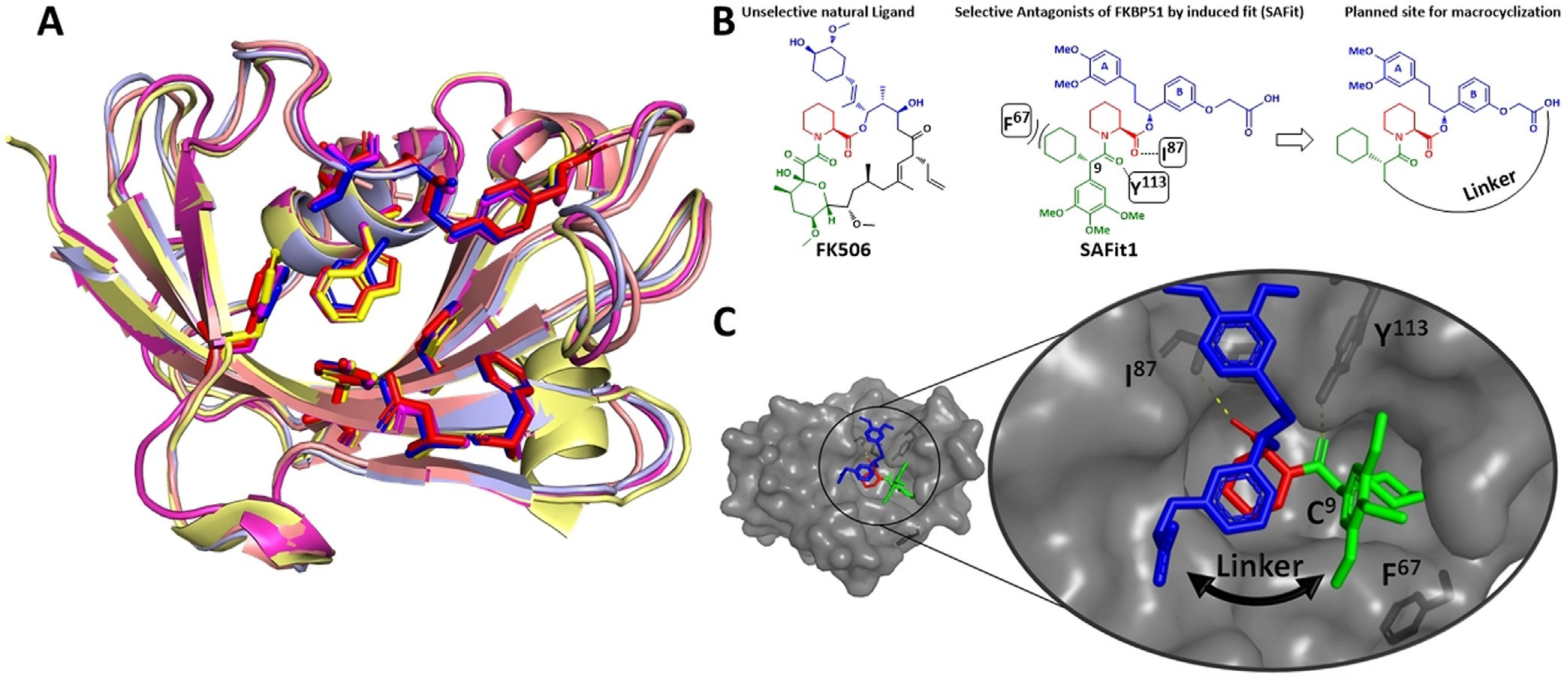
A) Superimposition of FKBP12 (red, PDB‐ID: 1FKJ), FKBP12.6 (blue, PDB‐ID: 5HKG), FKBP51 (yellow, PDB‐ID: 3O5R) and FKBP52 (magenta, PDB‐ID: 4LAX), in complex with FK506 or Rapamycin (not shown for clarity). B) The chemical structure of the FKBP ligands FK506 and SAFit1. The key interactions of SAFit1 with FKBP51 and the macrocyclization strategy are indicated. C) SAFit1‐analog iFit4 in complex with the FK1 domain of FKBP51 (PDB‐ID: 4TW7) highlighting the key interactions with the amino acid residues I^87^, Y^113^ and F^67^ and the structural basis for macrocyclization indicated by the black arrow.

A typical example is the family of FK506‐binding proteins (FKBPs) that possess a highly conserved binding pocket (Figure 1 A) but have diverged to perform diverse biological functions. The larger homolog FKBP51^[2]^ is a regulator of glucocorticoid receptor (GR) signaling^[3]^ and has emerged as a potential target for depression,^[4]^ obesity‐induced diabetes^[5]^ and chronic pain.^[6]^ In contrast, the homologous proteins FKBP12, FKBP12.6 and FKBP52 are considered anti‐targets due to their important roles in cardiology, sexual development and female infertility, emphasizing the need for selective inhibition.^[7]^

The most advanced FKBP51 ligands are compounds of the SAFit class (Selective Antagonists of FKBP51 by induced fit),^[8]^ which bind to a transient binding pocket unavailable to FKBP52^[1]^ and are up to 10 000‐fold selective for FKBP51 over FKBP52.[^8^, ^9^] However, SAFit‐like ligands still bind FKBP12 and its isoform FKBP12.6 with substantial affinities. These FKBPs are cofactors of the ryanodine receptor^[10]^ and play an important role in fine‐tuning the excitability of smooth or heart muscle. FKBP12 knockout or knockdown lead to severe cardiac defects in mice,^[11]^ underscoring the importance of selectivity for FKBP51 over FKBP12/12.6 in FKBP51‐based therapies.

Macrocyclization is a popular approach to improve drug‐like properties for compounds outside the rule‐of‐five space^[12]^ and is thought to be crucial for the unusually beneficial properties of the clinically used natural products FK506 (Figure 1 B), Rapamycin and Cyclosporin.^[13]^ Macrocyclization was key to enhance the affinities or physicochemical properties of synthetic ligands for FKBP12^[14]^ and cyclophilins.^[15]^ In a pilot study on the macrocyclization of SAFit analogs,^[16]^ we surprisingly observed a rearrangement of the FKBP51 binding pocket, which in turn allowed an unprecedented selectivity against the off‐targets FKBP12 and FKBP12.6.

## Results and Discussion

**Scheme 1:**
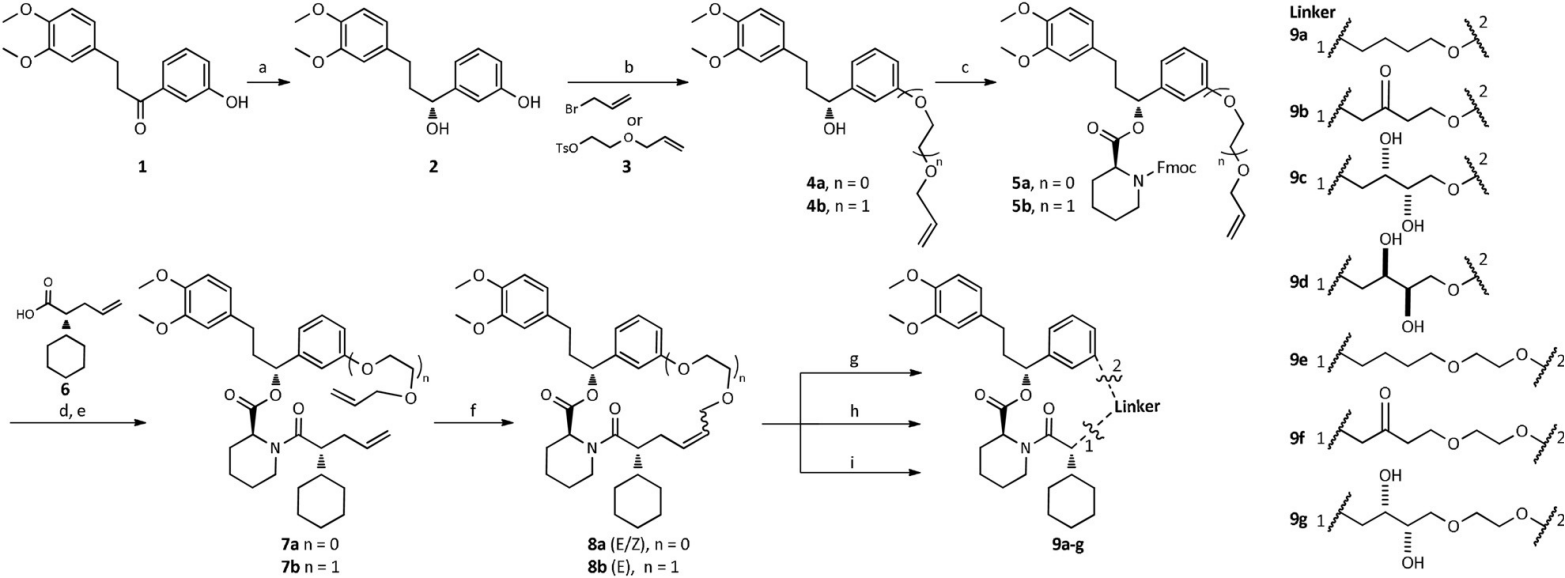
First generation of SAFit‐derived macrocycles. a) RuCl_2_[(*S*)‐(DM‐SEGPHOS®)][(*S*)‐DAIPEN], THF, 10 bar H_2_, KOtBu, rt; b) K_2_CO_3_, allyl bromide or **3**, MeCN, rt; c) DCC, DMAP, Fmoc‐*S*‐pipecolate, DCM; d) 20 % 4‐methylpiperidine in DMF; e) **6**, HATU, HOAt, DIPEA, DMF; f) Grubbs‐2 cat., DCM; g) Wilkinson cat. or Pt/C, DCM/MeOH, 1 bar H_2_; h) PdCl_2_, *p*‐benzoquinone, THF/H_2_O; i) OsO_4_, NMO, Ac/H_2_O.

Based on the structure–affinity relationship findings of SAFit analogs[^8^, ^9^] and the highly conserved binding mode (Figure 1 C), we chose to keep the pipecolate, the chalcone‐derived A/B rings, and cyclohexyl ring constant and to cyclize between the latter two. The synthesis started from compound **1**,^[8]^ where the ketone was reduced by an asymmetric Noyori catalyst to the chiral alcohol **2** and then coupled with allyl bromide or linker **3** to the alcohols **4 a**/**b** (Scheme 1). After coupling with Fmoc‐*S*‐pipecolate,^[17]^ **5 a**/**b** were deprotected and coupled with **6**.^[9b]^ The linear precursors **7 a**/**b** were cyclized by RCM to yield **8 a** and **b**. For **8 a** we were able to separate both *E* and *Z* isomer (ratio in crude mixture=89:11) and for the larger macrocycle **8 b** we only observed and isolated the *E* isomer. Unfortunately, none of these macrocycles showed detectable binding to FKBP51 in a fluorescence polarization assay.^[18]^ To introduce additional functionalities into the linker, we further derivatized the *E* isomers of **8 a**/**b** by Wacker oxidation, dihydroxylation or hydrogenation (**9 a**–**g**), which for **8 a**/**b** resulted in only one Wacker product (**9 b**/**f**). After dihydroxylation of the smaller macrocycle, we obtained the diastereomers (**9 c**/**d**) and an inseparable dihydroxylated diastereomeric mixture (**9 g**, dr=1:1 by NMR). Gratifyingly, the dihydroxylated derivative **9 g** of the larger macrocycle bound to FKBP51 with a *K*_i_ of 1.2 μm, whereas for **9 a**–**f** no binding to FKBP51 could be detected. To our great surprise, **9 g** did not bind to FKBP12 or FKBP12.6.

**Scheme 2:**
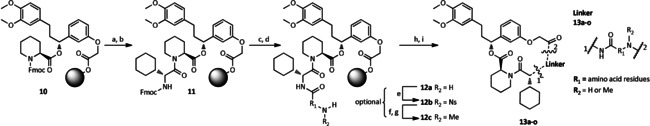
Second generation of SAFit‐derived macrocycles. The steps e, f and g are optionally applied in case of N‐methylation. a) 20 % 4‐methylpiperidine in DMF, rt, 3×10 min; b) Fmoc‐d‐Chg‐OH, HATU, HOAt, DIPEA, DMF; c) 5 % 4‐methylpiperidine in DMF, 0 °C, 3×5 min; d) Fmoc‐AA‐OH, HATU, HOAt, DIPEA, DMF; e) NosCl, Collidine, DMF; f) PPh_3_, dry MeOH, DIAD, dry THF, rt, 3×10 min; g) 2‐mercaptoethanol, DBU, DMF, rt, 3×10 min; h) 20 % HFIP in DCM; i) 1 mm in DMF, HATU, DIPEA.

Therefore, we set out to investigate this finding in more detail, resorting to amino acids to rapidly explore the effect of the linker (Scheme 2). Using an Fmoc SPPS strategy, we started from the immobilized SAFit1 precursor **10** ^[19]^ to introduce d‐cyclohexyl glycine as the FKBP52‐discriminating moiety, followed by coupling with a variety of amino acids yielding the immobilized intermediates. The deprotection of **11** had to be optimized to suppress diketopiperazine formation.^[20]^ Prior to derivatization of the intermediates **12 a** an optional N‐methylation sequence was included to probe the influence of the resulting amide groups (**12 a**–**c**).^[21]^ Finally, the linear peptides **12 a** or **12 c** were cleaved from the resin and cyclized by macrolactamization (**13 a**–**o**).

All final compounds were screened for affinity towards the FKBPs 12, 12.6, 51 and 52 in a competitive fluorescence polarization assay (Table 1).[^18^, ^22^]

**Table 1:** Affinities of the macrocycles with amino acid containing linker determined by a competitive fluorescence polarization assay.

Cmpd.	Linker	FKBP51FK1	FKBP12	FKBP12.6
		*K*_i_ [μm]^[a]^		
SAFit1	No linker	0.004±0.001^[b]^	0.163±0.009^[b]^	0.019±0.002^[b]^
FK506	Figure 1 A	0.104^[c]^	0.0006^[c]^	0.004^[c]^
**13 a**	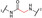	2.30±0.05^[d]^	>80	>80
**13 b**	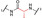	1.00	>80	>80
**13 c**	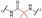	0.29±0.05^[b]^	>80	>80
**13 d**	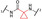	0.40±0.05^[b]^	>80	>80
**13 e**	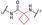	0.40	>80	>80
**13 f**	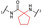	1.30	>80	>80
**13 g**	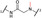	3.10	>80	>80
**13 h**	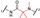	0.37	>80	>80
**13 i**	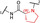	0.80	>80	>80
**13 j**	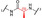	17	>80	>80
**13 k**	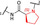	5.10	>80	>80
**13 l**	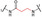	7.40	>80	>80
**13 m**	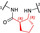	1.80	>80	>80

[a] The value >80 μm indicates the highest measurable concentration before compound precipitation; no binding to FKBP52FK1 was observed for any of the tested compounds up to 80 μm. [b] Standard error from three independent measurements. [c] Values derived from literature.^[23]^ [d] Error from two independent measurements.

Gratifyingly, most of the macrocycles with amino acid‐based linkers bound to FKBP51 in the low to submicromolar range and as expected none to FKBP52 (not shown). The glycine derivative **13 a** had an affinity of 2.3 μm, which gradually increased with increasing substitution (**13 b** (d‐Ala): 1.0 μm, **13 c** (Aib): 0.29 μm). For geminal cyclic amino acids the affinity slightly decreased with size (**13 d**: 0.40 μm, **13 e**: 0.40 μm, **13 f**: 1.3 μm). N‐methylation and N‐cyclization did not substantially affect affinity [**13 g** (R_2_=Me): 3.1 μm), **13 h** (Aib + R_2_=Me), **13 i** (d‐Pro): 0.8 μm].

In contrast, the l‐Ala derivative **13 j** bound more weakly, consistent with the substantially reduced affinity of the l‐Pro derivative **13 k**. Longer linkers such as β‐Ala **13 l** and GABA (no binding, not shown) displayed reduced affinity, which could be compensated by appropriate rigidification as in **13 m** (1.8 μm; other diastereomers were inactive, not shown). Notably, none of the tested linear precursors bound to FKBP51, underlining the significance of the macrocyclization. Most importantly, however, none of the macrocycles did show any affinity towards FKBP12 or FKBP12.6.

The affinity of **13 a** and **d** for FKBP51 was confirmed by isothermal calorimetry (ITC), yielding an enthalpy‐driven *K*_d_=3.6 μm ±0.9 μm for **13 a** and *K*_d_=0.6 μm ±0.1 μm for **13 d**, respectively (Figure S1).

**Figure 2:**
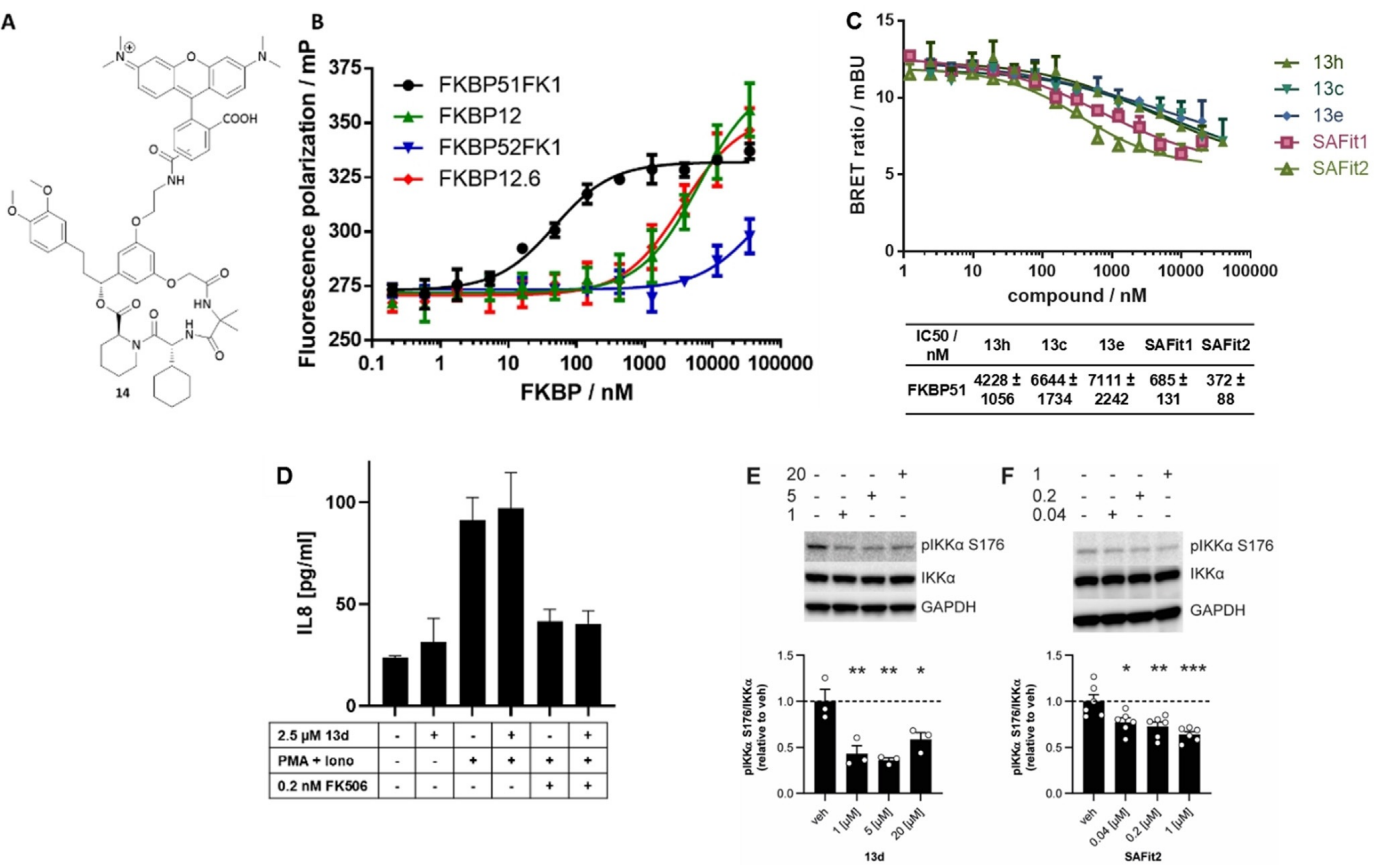
A) Structure of the TAMRA labelled tracer **14** [5/6‐TAMRA regio isomer]. B) Binding of the TAMRA‐tracer **14** to FKBPs 51, 52, 12 and 12.6 determined by a fluorescence polarization assay (*K*_d_^FKBP51FK1^=45±7 nm, *K*_d_^FKBP12^=6±1.4 μm, *K*_d_^FKBP12.6^=3.6±0.8 μm, *K*_d_^FKBP52FK1^ >80 μm). Error bars represent the standard deviation of three independent experiments. C) NanoBRET assay in HEK293T cells transiently expressing FKBP51‐NLuc. Data points represent means and standard deviations of three independent cellular assays per concentration. D) FK506, but not **13 d**, blocks the phorbol/ionomycin‐induced IL8 secretion in Jurkat cells and **13 d** does not interfere with FK506‐mediated immunosuppression. E,F) Inhibition of IKKα phosphorylation by **13 d** and SAFit2 in murine microglia SIM‐A9 cells. Bars below represent means and standard errors of three and six independent cellular assays, respectively, quantified by Western blots (for full blots see Supporting Information). *: *p*<0.05, **: *p*<0.01, ***: *p*<0.001.

We also prepared the fluorescent analog **14** of the best binding compound **13 c** (Figure 2 A, synthesis see Scheme S1), which bound in a fluorescence polarization assay with high affinity to FKBP51 (*K*_d_=45±7 nm) but poorly to FKBP12, FKBP12.6 or FKBP52 (Figure 2 B). The affinity of the tracer was further confirmed in a FRET assay with a fluorescein‐labeled FKBP51FK1 domain (*K*_d_=80±10 nm; Figure S2).

To check if compounds of the new class of macrocycles were able to engage FKBP51 inside cells, we performed a NanoBRET assay using a transiently expressed FKPB51–NLuc construct. The assay utilizes a fluorescent tracer for the FKBP51–Nluc construct, which accepts the luminescent energy to generate a BRET signal. If compounds engage the FKBP–Nluc construct inside cells, the tracer is displaced, reducing the BRET signal and allowing direct quantification of FKBP51–NLuc occupation. Representative macrocyclic compounds **13 c**, **13 e** and **13 h** as well as SAFit1 and ‐2 all dose‐dependently competed with a fluorescent NanoBRET tracer inside cells (Figure 2 C). The lower potencies of the macrocycles compared to SAFit1 and ‐2 are in line with the lower affinities of the macrocycles.

FK506 works as an immunosuppressant by an FKBP12‐dependent gain‐of‐function mechanism. A cellular analysis showed that compound **13 d** has neither immunostimulatory nor immunosuppressive properties on its own (Figure 2 D). Importantly, unlike the pan‐selective FKBP ligand [4.3.1]16h^[21]^ (Figure S6A) **13 d** also did not block the immunosuppressive activity of FK506, in line with its selectivity against FKBP12 (Figure 2 D).

We next explored if the macrocyclic ligands could interfere with the cellular functions of FKBP51. We therefore treated SIM‐A9 cells, which were recently discovered as a SAFit‐sensitive cellular model for stress‐mediated secretory autophagy.^[24]^ Compound **13 d** (Figure 2 E) as well as compounds **13 c**, **13 e**, **13 h** and **13 i** (Figure S6B) all inhibited IKKα phosphorylation, similar to SAFit1 and SAFit2^[4f]^ (Figure 2 F and S6C). For compounds **13 e**, **13 i**, SAFit1 and SAFit2 a clear dose‐dependence was observed. For **13 c**, **13 d** and **13 h**, the apparent maximal inhibition was already reached at the lowest tested concentration of 1 μm, possibly reflecting the higher affinities and/or improved cell permeability of **13 c**, **13 d** and **13 h** compared to **13 e** and **13 i**. Taken together, these results show that the here discovered macrocycles can penetrate human cells, intracellularly occupy FKBP51 and interfere with its function.

**Figure 3:**
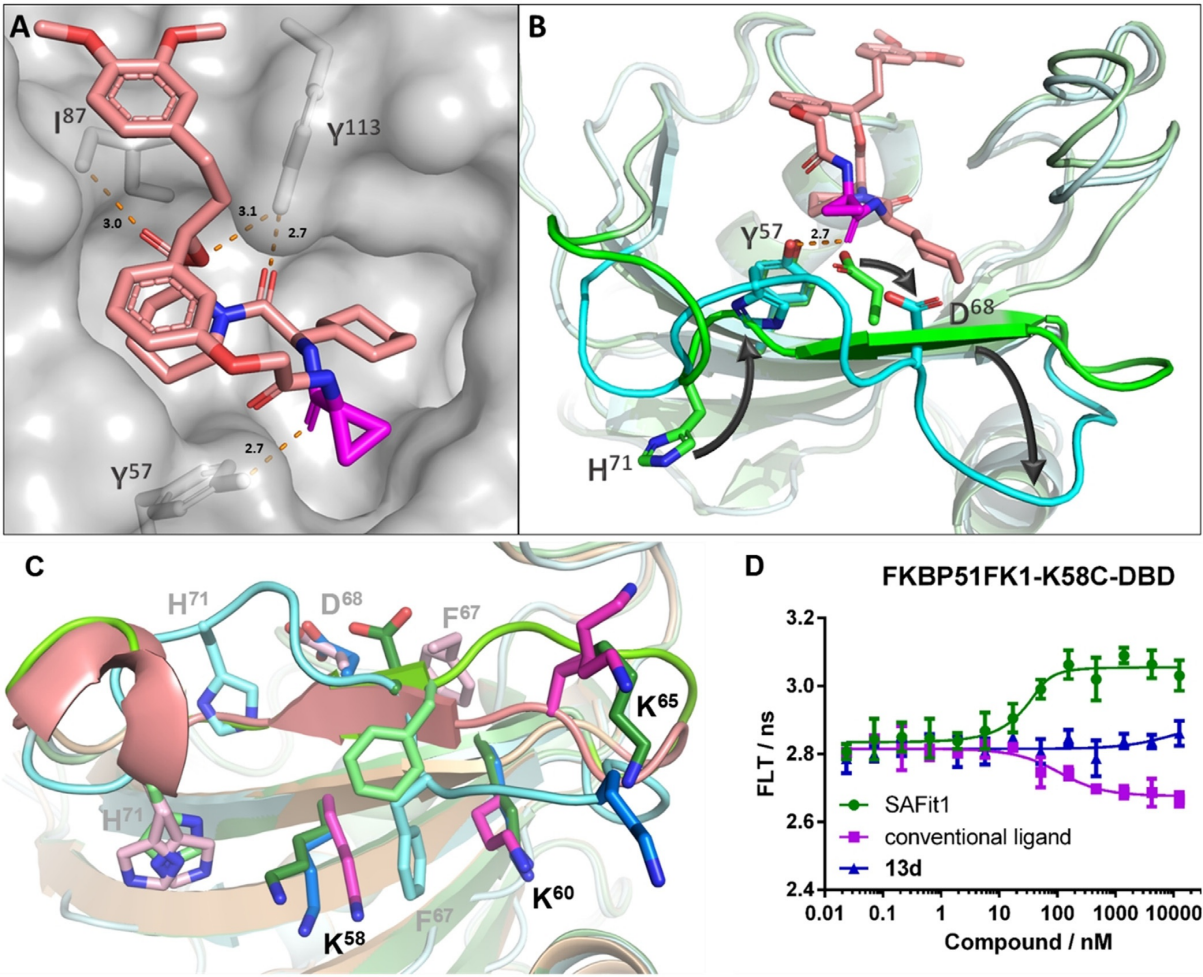
A) Crystal structure of the FK1 domain of FKBP51 in complex with **13 d** (pink‐colored sticks), key interactions with the residues I^87^, Y^113^ and Y^57^ indicated as orange broken line (distance annotated in Å), PDB‐ID: 7AOU. B) Crystal structure of the FK1 domain of FKBP51 (pale cyan) in complex with **13 d** (pink‐colored sticks) superimposed to the cocrystal structure of the SAFit1‐analog iFit4 in complex with FKBP51FK1 (pale green, PDB‐ID: 4TW7, iFit4 has been omitted for clarity). The β3b strand is highlighted in cyan and green, respectively, and key residues Y^57^, D^68^ and H^71^ are shown as sticks. The key carbonyl of **13 d** displacing D^68^ is highlighted in magenta and the new hydrogen bond between Y^57^ and the **13 d** carbonyl is indicated as orange broken line (distance annotated in Å). C) Side view of FKBP51 from cocrystal structures with **13 d** (pale cyan, 7AOU), with the SAFit analog iFit4 (pale green, 4TW7), and the conventional binding‐mode ligand [4.3.1]‐16h (pale magenta, 5OBK). The residues F^67^, D^68^ and H^71^ are shown as pale sticks. K^58^, K^60^ and K^65^ as the attachment point for the environment‐sensitive dye are highlighted as intense colored sticks. D) Fluorescence life‐time analysis of DBD‐labeled FKBP51FK1^K58C^ in the presence of the indicated ligands.

To clarify the structural basis for this unprecedented selectivity, we solved the cocrystal structures of **13 a**, **13 d** and **13 h** in complex with FKBP51 (Figure 3 A and S7A/C; PDB‐ID: 7AOU, 7AOT, 7AWF).^[25]^

As intended, the interactions of the pipecolate, the A‐ and B‐rings, as well as the cyclohexyl group with FKBP51 were completely conserved in comparison to previous FKBP51‐SAFit co‐crystal structures (Figure 3 A). This includes a displacement of F^67^, which is responsible for the strong selectivity vs. FKBP52 of SAFit‐like ligands.[^8^, ^9^] However, the β3b strand, which contains F^67^ and which we and others previously showed to display enhanced basal mobility,[^1^, ^26^] was substantially rearranged (Figure 3 B and S7B/D). Strikingly, we observed that the carbonyl group of **13 a**, **13 d** and **13 h** displaced D^68^ and replaced it as a hydrogen bond acceptor for the ϵ‐hydroxy group of Y^57^. The rearrangement of the β3b strand is stabilized by an inward flip of H^71^, which partially replaces S^70^ and substitutes the former as a hydrogen bond donor for the backbone carbonyl of Y^57^. A similar inward flip of H^71^ has previously been observed for FKBP51 in complex with Rapamycin and FRB (PDB‐ID: 4DRH).^[27]^ Intriguingly, H^71^ is replaced in FKBP12 and FKBP12.6 by an arginine (R^40^ in FKBP12/12.6 numbering), which can be expected to be less efficient in stabilizing the **13 a**‐binding conformation, providing a molecular rationale for the discrimination vs. FKBP12/12.6 observed for the macrocycles.

To clarify if the structural rearrangement of the β3b strand was also stabilized in solution, we developed a set of conformation‐sensitive assays^[28]^ that are responsive to alterations of the β3b strand. Towards this end, we introduced environment‐responsive dyes selectively at positions 58, 60 and 65 in the β2 strand below the β3 strand or in the β2–β3b loop (Figure 3 C). Remarkably, all three sensors clearly differentiated between ligands with canonical and a SAFit‐like binding mode (Figure 3 D and S8A/B). When using the K^60^C‐ and K^65^C‐based sensors, compound **13 d** induced similar changes in fluorescence lifetime as SAFit1, but with lower potency in accordance with its lower affinity. However, the K^58^C‐based sensor clearly differentiated macrocycle **13 d** from both SAFit1 as well as canonical FKBP ligands (Figure 3 D). This strongly suggests that the new peptide‐based macrocycles stabilize a new conformation in solution that is different from the known FK506‐like or SAFit‐like ligands.

## Conclusion

Macrocycles have repeatedly been discussed to impart improved physicochemical properties. However, they have rarely been associated with selectivity. Here we show that macrocycles can also provide the basis for subtype selectivity. In this particular case, the macrocycles provide the scaffold for the proper positioning of the key carbonyl group that displaces Asp^68^, which was not possible in the linear analogs. With regards to FKBP51, our results provide the first ligands that robustly discriminate between FKBP51 and FKBP12/FKBP12.6 and provide a structural basis for the rational design for further optimization regarding affinity, stability, specificity and cellular activity.

## Conflict of interest

The authors declare no conflict of interest.

## Supporting information

As a service to our authors and readers, this journal provides supporting information supplied by the authors. Such materials are peer reviewed and may be re‐organized for online delivery, but are not copy‐edited or typeset. Technical support issues arising from supporting information (other than missing files) should be addressed to the authors.

Supplementary
