# Supplementary material for: Macrocyclic FKBP51 Ligands Define a Transient Binding Mode with Enhanced Selectivity
Source: Angew Chem Int Ed Engl. 2021 May 7;60(24):13257–63. doi: 10.1002/anie.202017352 (PMC8252719; doi:10.1002/anie.202017352)
Supplement: Supplementary file 1 — Supplementary [file ANIE-60-13257-s001.pdf]

Angewandte Chemie
Eine Zeitschrift der Gesellschaft Deutscher Chemiker

Supporting Information

Macrocyclic FKBP51 Ligands Define a Transient Binding Mode with Enhanced Selectivity

*Andreas M. Voll, Christian Meyners, Martha C. Taubert, Thomas Bajaj, Tim Heymann, Stephanie Merz, Anna Charalampidou, Jürgen Kolos, Patrick L. Purder, Thomas M. Geiger, Pablo Wessig, Nils C. Gassen, Andreas Bracher, and Felix Hausch**

anie_202017352_sm_miscellaneous_information.pdf

1. ITC of compounds 13a and 13d 3

2. FRET assay of tracer 14a........ 4

3. Synthesis of the macrocyclic tracer 14a 5

4. FP-Assay 6

4.1 Binding curves of 13a, c and d 6

5. Cellular Assays……..8

5.1 Immunostimulation/supression in JURKAT cells 8

5.2 IKKα inhibition in SIM-A9 cells 8

5.3 Competitive NanoBRET 11

6. Procedure of co-crystallization 12
6.1. Crystallization 12
6.2. Structure solution and refinement 12
6.3. Refinement data........ 12

7. Co-crystal structure of compound 13a and 13h 13

8. Fluorescence Life Time Analysis……...14

9. Compound synthesis........ 15
9.1 General information 15
9.2. Ring closure by metathesis and derivatization (1st macrocycle generation) 15
9.3. Solid phase peptide synthesis (SPPS, 2nd macrocycle generation) 32
9.3.1 General procedures........ 32
9.3.2 Macrocycle synthesis 33
9.4. Tracer synthesis........ 47

10. Analytical data (HPLC, NMR)........ 55

11. References........ 98

12. Author contributions........ 98

1. ITC of compounds 13a and 13d

Figure S1. A) Binding of compound **13a** to FKBP51FK1 determined by isothermal titration calorimetry at 25 °C. A 20 µM solution FKBP51FK1 formulated in 20 mM HEPES pH 8.0, 20 mM NaCl, 5% glycerol and 2% DMSO was placed in the sample cell of a PEAK-ITC instrument. The syringe was filled with a 200 µM solution of **13a** formulated in the same buffer. After equilibration the compound was titrated into the sample cell by 12 injections of 3 µl each. The obtained data was analyzed using the provided software package and fitted to a one-site binding model yielding a K_d-value of 3600 ± 900 nM (mean ± SD from 3 independent experiments). **B)** Binding of compound **13d** to FKBP51FK1 determined by isothermal titration calorimetry at 25 °C. A 13 µM solution FKBP51FK1 formulated in 20 mM HEPES pH 8.0, 20 mM NaCl, 5% glycerol and 2% DMSO was placed in the sample cell of a PEAK-ITC instrument. The syringe was filled with a 100 µM solution of **13d** formulated in the same buffer. After equilibration the compound was titrated into the sample cell by 12 injections of 3 µl each. The obtained data was analyzed using the provided software package and fitted to a one site binding model yielding a K_d-value of 600 ± 100 nM (mean ± SD from 3 independent experiments).

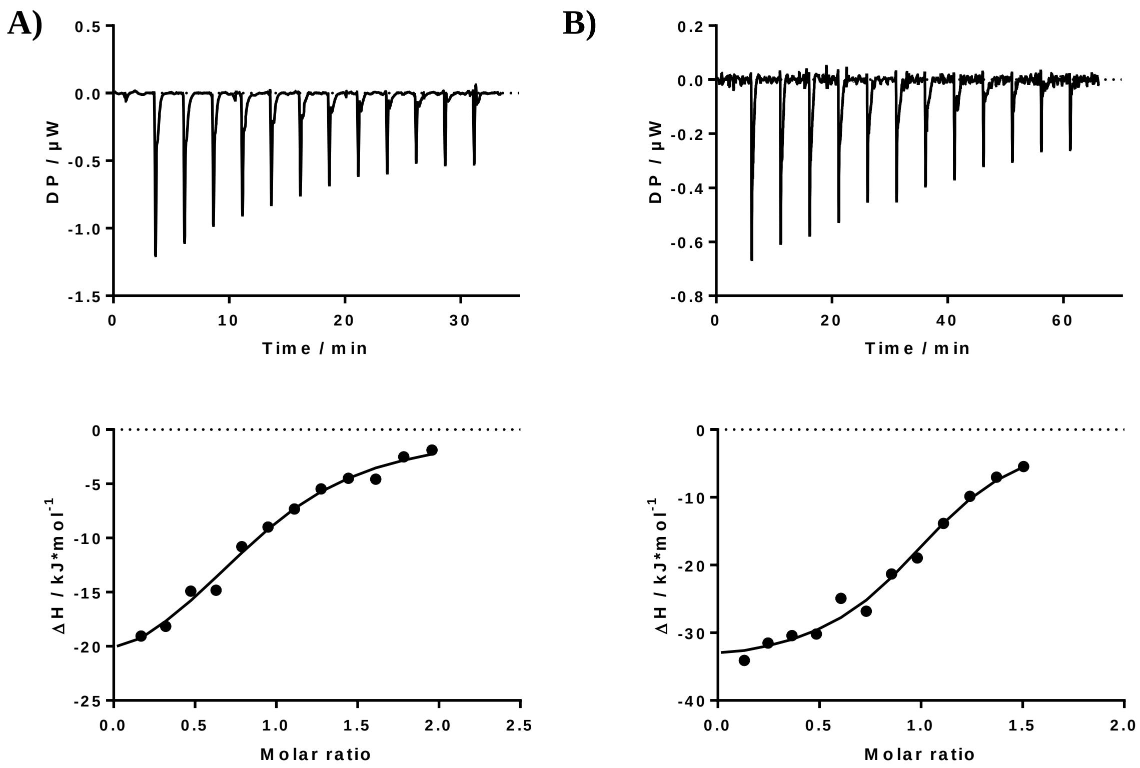

2. FRET assay of tracer 14a

Figure S2. Binding of the tracer **14a** to fluorescein-labeled FKBP51FK1 determined by a FRET binding assay. A serial dilution series of the tracer was placed in a microplate and supplemented with 5 nM of fluorescein-labeled FKBP51FK1. The binding of the tracer resulted in quenching of the fluorescein signal observable as decrease in the fluorescence intensity at 530 nm upon excitation at 485 nm. The obtained data were plotted against the tracer concentration and fitted to a binary binding model yielding a K_d-value of 80 ±10 nM. The error bars represent the standard deviation of three independent experiments.

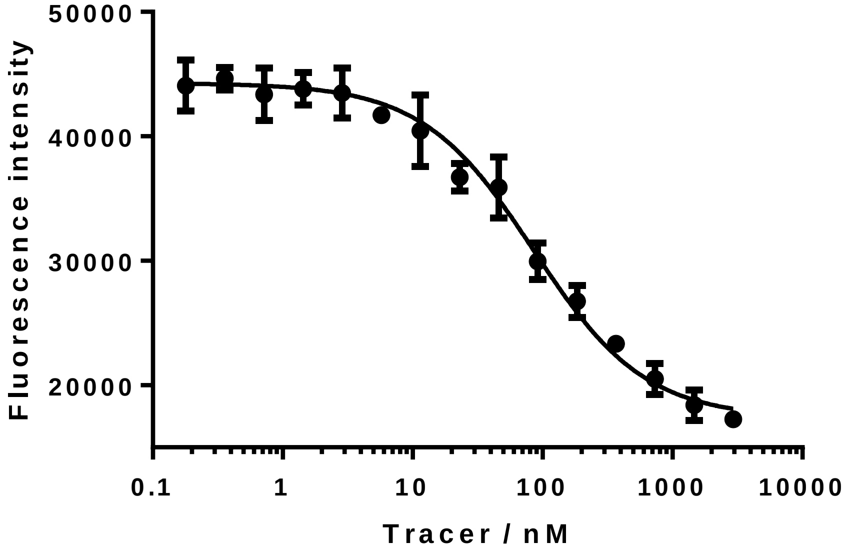

3. Synthesis of the macrocyclic tracer 14a

Scheme S1. Reaction scheme for the meta B ring modification and the TAMRA coupled fluorescent tracer. a) NaOH, EtOH/H_2O, 0°C-rt; b) Zn, NH_4Cl, MeOH; c) $RuCl_2$[(*S*)-(DM-SEGPHOS)][(*S*)-DAIPEN], THF, 10 bar H_2, KOtBu, rt; d) TrtCl, K_2CO_3, MeCN; e) **19**, K_2CO_3, MeCN; f) DCC, Fmoc-*S*-Pipecolate, DMAP, DCM; g) 1% TFA in DCM; h) 2-CTC-resin, DIPEA, DCM; i) 20% 4-Me-piperidine in DMF; j) Fmoc-D-Chg-OH, HATU, HOAt, DIPEA, DMF; k) 5% 4-Me-piperidine in DMF, 0°C, 3x5 min; l) Fmoc-Aib-OH, HATU, HOAt, DIPEA, DMF; m) $Pd(OAc)_2$, morpholine, PPh_3, THF; n) 20% HFIP in DCM; o) HATU, pentafluoro phenol, 1 mM in DMF, p) MeI, K_2CO_3, q) Ag_2CO_3, *tert*-butyl (2-bromoethyl) carbamate; (MeCN) r) DCM/TFA, 4/1 s) TEA, 5 (6)-NHS-TAMRA, (DMF).

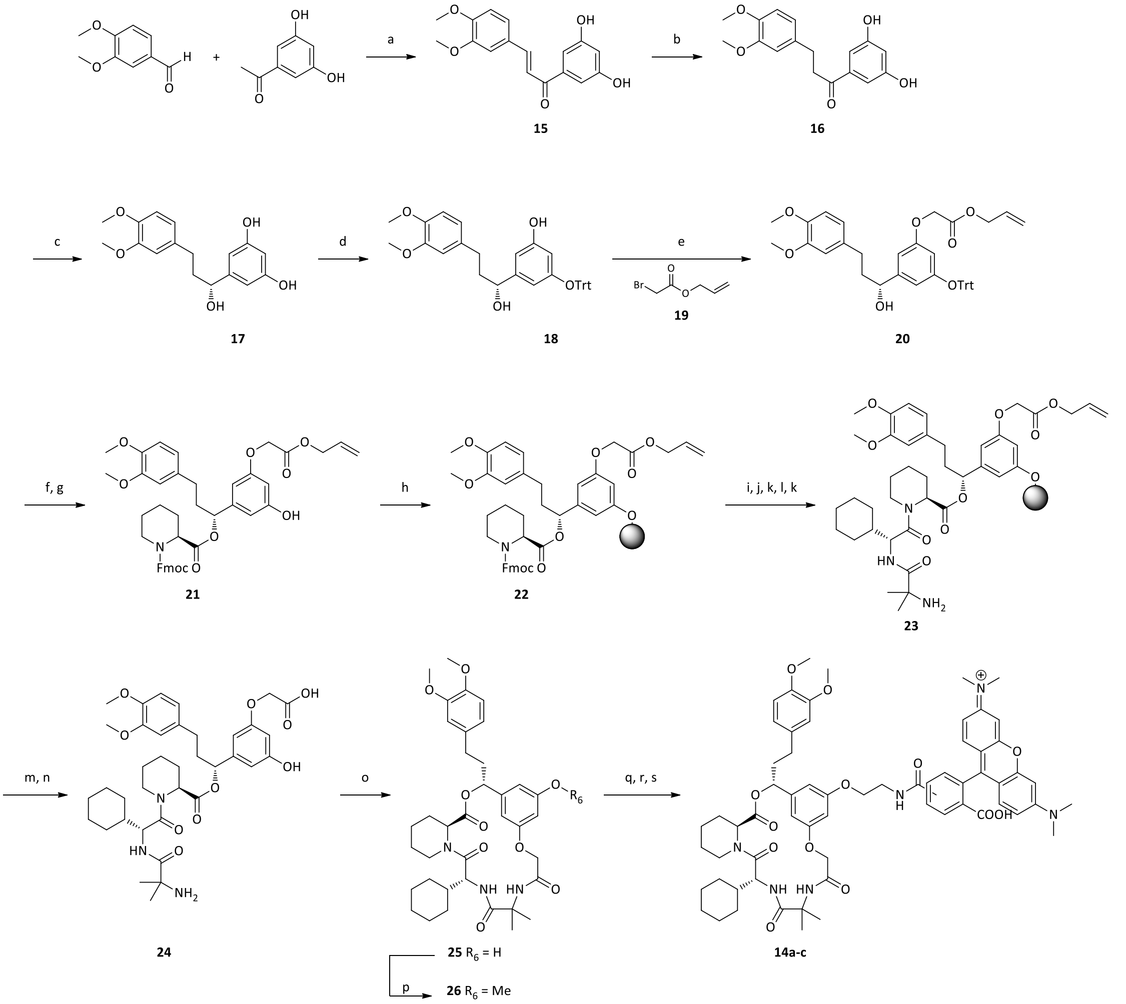

The synthesis started with the *Claisen-Schmidt* reaction of the respective ketone and aldehyde resulting in the chalcone **15**, which was chemo-selectively (**16**) and then asymmetrically reduced (**17**). The diphenol needed protection for further handling, which was done by mono tritylation (**18**) followed by reaction with linker **19**. Compound **20** was then coupled with Fmoc-*S*-pipecolate and the trityl group was removed yielding **21**. The free phenol was loaded onto 2-chlorotrityl chloride (CTC) resin (**22**) and further coupled under standard SPPS conditions providing **23**. The allyl ester was deprotected, the construct was cleaved from the resin (**24**), cyclized in solution (**25**) and further methylated (**26**). [Cyclization on solid support was tested but resulted in incomplete conversion] For the cyclization reaction in solution, a side product with fitting mass to the guadinylated free phenol was observed in quite high amounts. This was prevented using pentafluoro phenol as scavenger of the reactive species. The macrocyclic tracer was synthesized starting from compound **25**. First a linker was added by nucleophilic substitution reaction with *tert*-butyl-(2-bromoethyl) carbamate, which was deprotected with TFA and finally coupled with a *N*-hydroxy succinimide activated 5 (6)-TAMRA fluorophore [5(6)-Carboxytetramethylrhodamine *N*-succinimidyl ester] providing the regioisomers of compound **14**. The regioisomers could be separated and three fractions were obtained. **14a** (pure regioisomer 1), **14b** (mixed fraction of both isomers 34/66) and **14c** (enriched regioisomer 2 still containing 12% isomer 1). All three fractions were tested for binding in an FP-assay with **14a** having the highest affinity.

The meta hydroxy (**25**) and methoxy (**26**) substitutions were also tested in an FP-assay (**Table S1)** and displayed a slightly decreased affinity towards FKBP51 (0.37-0.39 µM) in comparison to its parent compound **13d** and no binding of FKBP52, 12 and 12.6.

Table S1. K_i values were determined by a competitive fluorescence polarization assay.

Cmpd.	R_6	FKBP51
		K_i [µM]
**13d**	No substituent	0.29
**25**	H	0.37
**26**	Me	0.39

4. FP-Assay

4.1 Binding curves of 13a, c and d

Figure S3. FP-Assay binding curves of compound 13a.

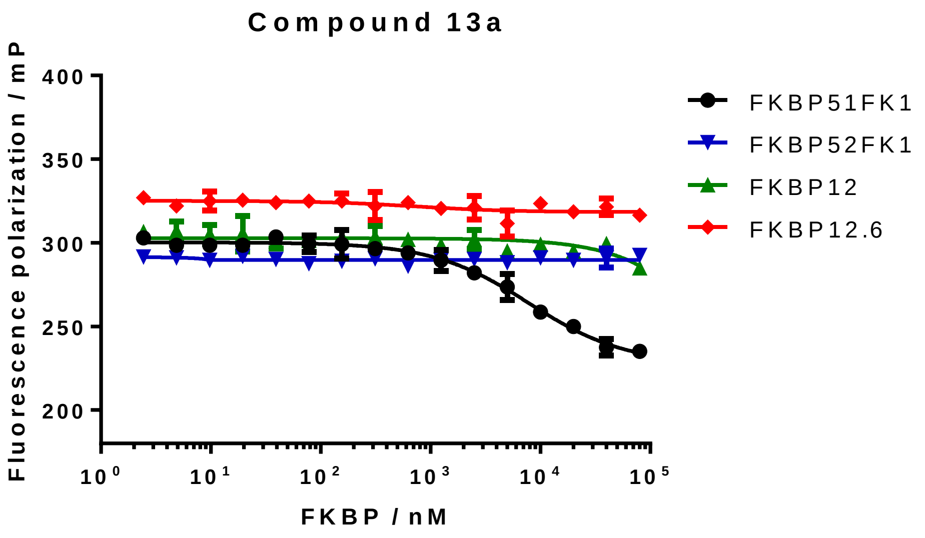

Figure S4. FP-Assay binding curves of compound 13c.

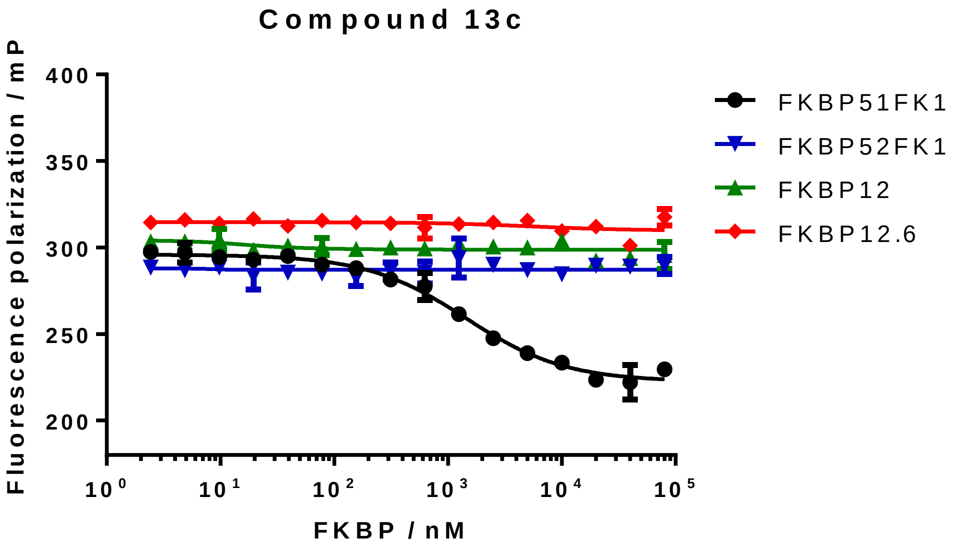

Figure S5. FP-Assay binding curves of compound 13d.

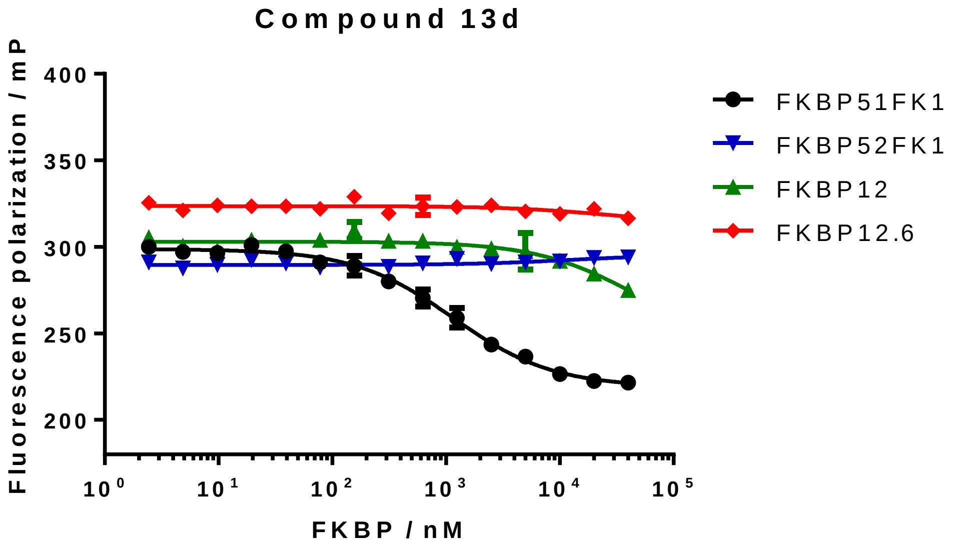

5. Cellular Assays

5.1 Immunostimulation/suppression assays in JURKAT cells

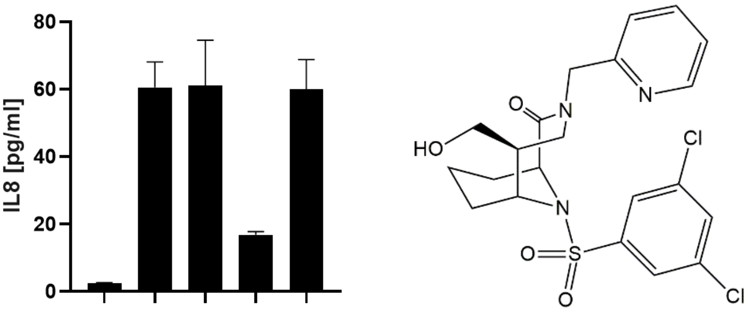

10 µM FK[431]-16h	-	-	+	-	+
PMA + Ionomycin	-	+	+	+	+
0.2 nM FK506	-	-	-	+	+

FK[431]-16h
(corresponds to compound 16h from Pomplun et al, J Med Chem 2018, 61, 3660-73)

Figure S6A. Cellular analysis of compound FK[431]-16h in Jurkat cells

150 000 Jurkat cells were seeded in a 96 well plate in 180 µl and treated for 30 minutes with **13d** or FK[431]-16h at the indicated dosages. 0.2 nM FK506 was added for an additional 30 minutes and 50 ng/ml PMA and 750 ng/ml Ionomycin for additional six hours. Cells were pelleted and supernatant collected and stored at -80°C. The IL8 content in the supernatant was determined using the human IL8/CXCL8 DuoSet Elisa kit (RndSystems) according to manufacturers instructions. The sample volume of supernatant was adapted to 150 µl. Optical density was assessed using a Tecan Spark.

5.2 IKKα inhibition in SIM-A9 cells

Cell culture and treatments

Murine microglia SIM-A9 cells were cultured at 37 °C, 5% CO_2 in Dulbecco's Modified Eagle Medium (DMEM) high glucose with GlutaMAX (Thermo Fisher Scientific, 10566016), supplemented with 1 mM sodium pyruvate (Thermo Fisher Scientific, 11360-070), 10% fetal bovine serum (PAN Biotech, P30-8500), and 1% antibiotic-antimycotic (Thermo Fisher Scientific, 15240-062).

Cell lysis

For the preparation of whole-cell lysates, confluent SIM-A9 cell cultures treated with the indicated compounds for 16 h in the above mentioned growth medium, were washed two times with ice-cold DPBS (Thermo Fisher Scientific, 14190-144) and lysed in an appropriate volume of RIPA buffer (Thermo Fisher Scientific, 89901), freshly supplemented with 1x cOmplete™ EDTA-free protease inhibitor cocktail (Roche, 4693132001) and 1x PhosSTOP™ phosphatase inhibitor cocktail (Roche, 4906837001) for 20 min on ice. Subsequently, samples were centrifuged for 10 min at 4°C at 13000 rpm. The resulting supernatants were transferred to new 1.5 ml reaction tubes and prepared for separation with an SDS-PAGE system and analyzed by immunoblotting.

SDS-PAGE and immunoblotting

Separation of proteins was performed with the Criterion™ Vertical Electrophoresis Cell System (Bio-Rad, 1656001) on 4–20% Criterion™ TGX™ Precast Midi Protein Gels (Bio-Rad, 5671095). Immunoblotting was performed with the Trans-Blot Turbo Transfer System (Bio-Rad, 1704150) on 0.2 µm PVDF Trans-Blot Turbo Transfer Membranes (Bio-Rad, 1704157). For immunodetection, membranes were placed in TBS, supplemented with 0.05% Tween (Sigma-Aldrich, P9416) and 5% non-fat milk (Carl Roth, T145.1) for 1 h at RT and then incubated with the primary antibody diluted in TBS-T overnight at 4°C. Following primary antibodies were used: Phospho-IKKα S176/phospho-IKKβ S177 (1:1000, Cell Signaling Technology, 2078), IKKα (1:1000, Cell Signaling Technology, 1682) and GAPDH (1:1000, Cell Signaling Techology, 5174).
Then, immunoblots were washed and probed with horseradish peroxidase- conjugated secondary antibody (1:10000, Cell Signaling Technology, 7074) for 1 h at RT. Immunoreactive protein bands were visualized using Clarity Western ECL Substrate (Bio-Rad, 1705061) or Clarity Max Western ECL Substrate (Bio-Rad, 1705062). Determination of the band intensities was performed with ChemiDoc MP (Bio-Rad).

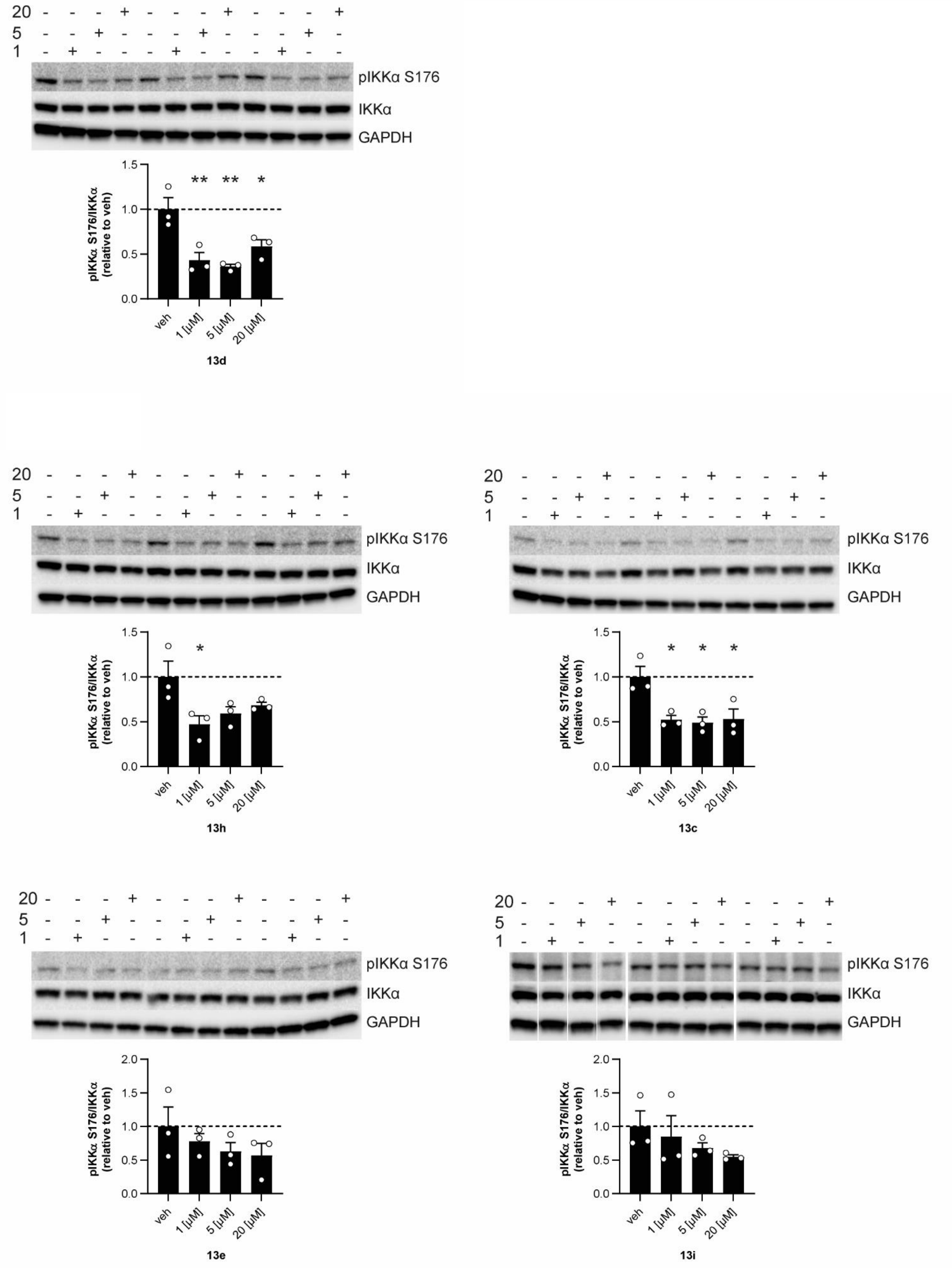

Fig. S6B: Inhibition of IKKα phosphorylation by compound 13c,e,h,I in murine microglia SIM-A9 cells. Bars below repreent means and standard errors of three independent cellular assays quantified by Western blots.

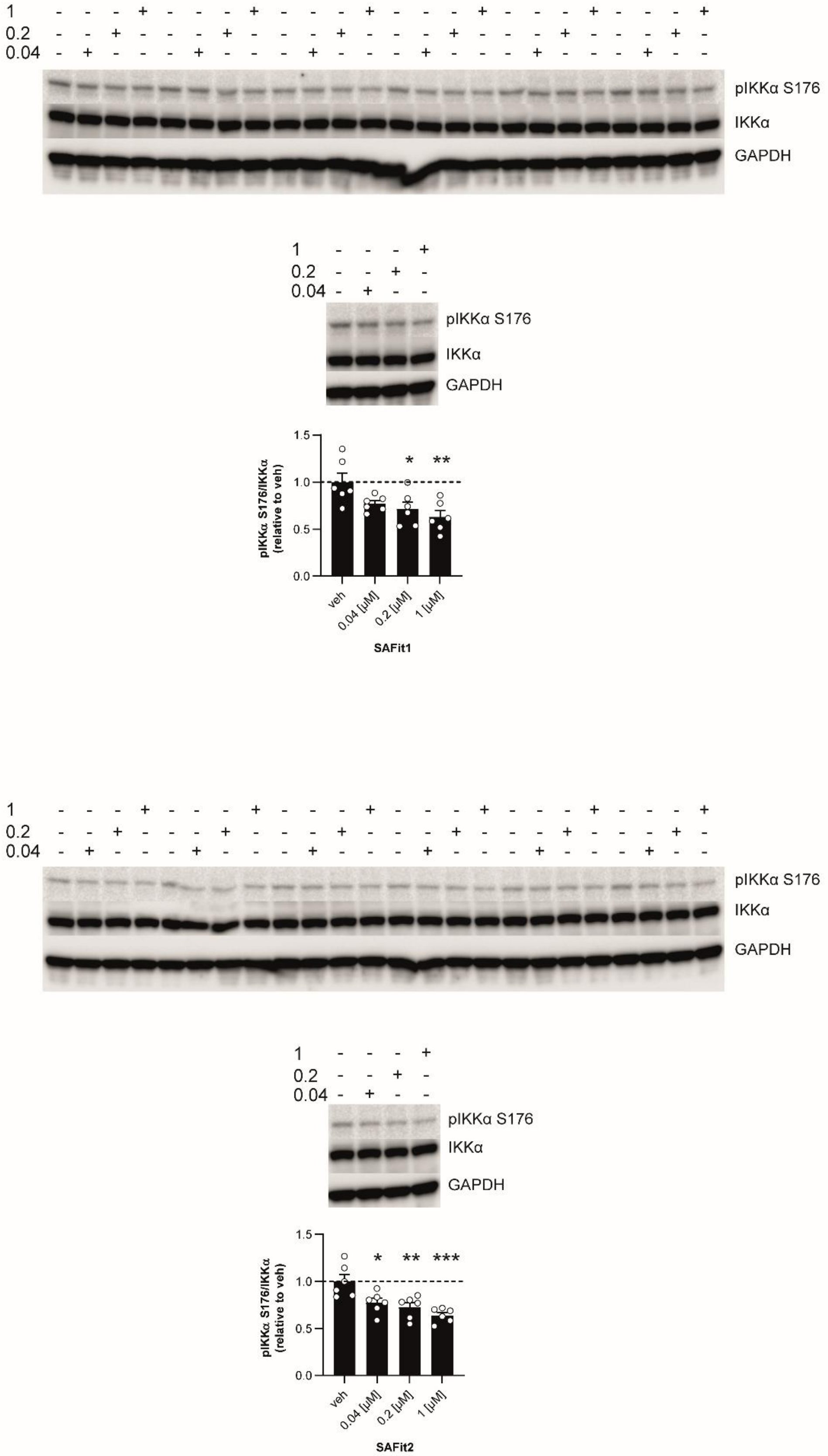

Fig. S6C: Inhibition of IKKα phosphorylation by SAFit1 and SAFit2 in murine microglia SIM-A9 cells. Bars below represent means and standard errors of six independent cellular assays quantified by Western blots.

5.3 Competitive NanoBRET

HEK239T cells were cultured at 37 °C, 5% CO2 in Dulbecco's Modified Eagle Medium (DMEM) (Gibco-Invitrogen, 41966-029) high supplemented with 10% fetal bovine serum (Gibco-Invitrogen, 10500-064) and 1% Pen Strep (Gibco-Invitrogen, 15140-122).

500000 HEK293T cells were seeded in a Poly-L-lysine coated 6 cm plate and incubated for 24 h. Next, the cells were transiently transfected with 500 ng pNLF1-FKBP51-Nluc according to the Lipofectamin 2000 (Invitrogen, 11668-019) protocol. Cells were used 24 h post transfection. The cells were detached form the dish, resuspended in Opti-MEM I Reduced Serum Media (Gibco-Invitrogen, 11058-021) and cell number was adjusted to 450000/mL. The cell tracer mixture was prepared mixing one part of the tracer solution 1,6 µM tracer Opti-MEM I Reduced Serum Media) with three parts cell suspension.

Test ligands were dissolved in DMSO (1000x concentration required for the final sample) and 1:2 serial diluted in DMSO. Following, each dilution was 1:500 diluted in Opti-MEM I Reduced Serum Media. Next, 20 µL/well compound solution and 20 µL/well cell-tracer mixture were added to a white non binding assay plate (No.: 3574; Corning Life Sciences B.V.) and incubated for 2 h at 37°C. Afterwards the plate was equilibrated at room temperature for 15 minutes. TE NanoGlo® Substrat/Inhibitor (No.:N2160; Promega) and a Tecan Spark used for BRET detection. The NanoBRET™ NanoGlo® Substrate and the extracellular NanoLuc® inhibitor were diluted 1:664 and 1:2000, respectively, in Opti-MEM I Reduced Serum Media. 20 µL/well was added and the plate was incubated for three minutes at room temperature. The donor emission was measured at 455-470 nm and the acceptor emission at 610-700 nm. The BRET ratio was calculated according to the following formula: $BRET\ ratio = \frac{I(acceptor)}{I(donor)}$

6. Procedure of co-crystallization

6.1. Crystallization

Complexes were prepared by mixing FKBP51 at 1.94 mM (**13a**), 2.5 mM (**13d**) and with a slight molar excess of 4.44 mM (**13a**) and 20 mM (**13d**) ligand dissolved in DMSO. Crystallization was performed at 20°C using the hanging drop vapor-diffusion method, equilibrating mixtures of 1 µl protein complex and 1 µl reservoir against 500 µl reservoir solution. Crystals were obtained after seeding with reservoir solutions containing 15-40% PEG-3350, 0.2 M NH_4-acetate and HEPES-NaOH pH 7.5 or 0.2 M NH_4-thiocyanate and 0.1 HEPES-NaOH pH 7.5.

6.2. Structure solution and refinement

Diffraction data were collected at the European Synchrotron Radiation Facility (ESRF) in Grenoble, France and Swiss Light Source (SLS) in Aargau, Swiss. Diffraction data were integrated with XDS[1] and further processed with the implemented programs of the CCP4i and CCP4i2 interface (Collaborative Computational Project).[2] The data reduction was conducted with Aimless.[2b, 3] Crystal structures were solved by molecular replacement employing the program Molrep.[4] Iterative model improvement and refinement were performed with Coot[5] and Refmac5.[6] The dictionaries for the compounds were generated with PRODRG implemented in CCP4i.[7] Residues facing solvent channels without detectable side-chain density were truncated at Cβ.

6.3. Refinement data

Table S1. Refinement data of compound **13a**, **13d** and **13h**

Lig. Name	13a	13d	13h
Lig. Structure	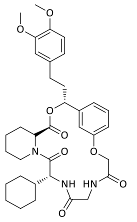	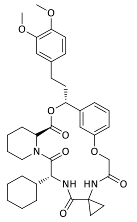	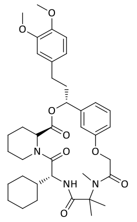
PDB-ID	7AOT	7AOU	7AWF
Protein	FKBP51fk1 A19T	FKBP51fk1 A19T	FKBP51fk1 A19T
Synchrotron	ESRF	ESRF	SLS
Beamline	ID30B	ID23-1	X06SA
Space group	$P2_12_12_1$	$P2_12_12_1$	$P2_12_12_1$
a (Å)	43.504	43.410	42.460
b (Å)	49.633	49.460	50.066
c (Å)	59.903	59.590	58.781
α (°)	90	90	90
β (°)	90	90	90
γ (°)	90	90	90
Integration software	XDS	*GrenADES/ EDNA*	XDS
Resolution limits (Å)	49.63 – 0.85	38.06 - 1.16	38.11 – 1.42
⟨$I/\sigma(I)$⟩	22.1 (1.4)	9.8 (2.1)	12.8 (0.7)
Multiplicity	5.7 (3.3)	1.6 (1.9)	3.7 (4.0)
Completeness (%)	97.1 (72.4)	99.8 (99.3)	99.8 (93.5)
Refinement program	Refmac5	Refmac5	Refmac5
Resolution range (Å)	30.00 – 0.85	29.80 - 1.16	38.00 – 1.40
$Reflections_{all}$	105556	45040	24745
$Reflections_{free}$	5557	2243	1242
R_{work}	0.122	0.143	0.194
R_{free}	0.138	0.175	0.243
No. of atoms (B-Factor)			
Protein	2109 (11.5)	2106 (17.3)	1914 (23.4)
Ligand	91 (8.0)	95 (13.7)	100 (19.5)
Water	223 (23.4)	194 (28.8)	113 (34.6)
R.m.s.d. bonds (Å)	0.030	0.015	0.017
R.m.s.d. angles (°)	2.522	1.971	1.946

7. Co-crystal structure of compound 13a and 13h

Figure S7. A) Crystal structure of the FK1 domain of FKBP51 in complex with **13a** (pink-colored sticks), key interactions with the residues I^{87}, Y^{113} and Y^{57} indicated as yellow broken line (distance in Å). The linker is highlighted in magenta. pdb-ID: 7AOT. **B)** Crystal structure of the FK1 domain of FKBP51 (pale cyan) in complex with **13a** (pink-colored sticks) superimposed to the structure of the SAFit-analog iFit4-FKBP51 complex (pale green, pdb-ID: 4tw7, iFit4 has been omitted for clarity). The β3b strand is highlighted in cyan and green, respectively, and key residues Y^{57}, D^{68} and H^{71} are shown as sticks. The key carbonyl of **13a** is displacing D^{68} and the new hydrogen bond between Y^{57} and D^{68} in the **13a** complex is indicated as a yellow broken line. **C)** Crystal structure of the FK1 domain of FKBP51 in complex with **13h** (pink-colored sticks), key interactions with the residues I^{87}, Y^{113} and Y^{57} indicated as yellow broken line (distance in Å). pdb-ID: 7AWF. **D)** Crystal structure of the FK1 domain of FKBP51 (pale cyan) in complex with **13h** (pink-colored sticks) superimposed to the structure of the SAFit-analog iFit4-FKBP51 complex (pale green, pdb-ID: 4tw7, iFit4 has been omitted for clarity). The β3b strand is highlighted in cyan and green, respectively, and key residues Y^{57}, D^{68} and H^{71} are shown as sticks. The key carbonyl of **13h** is displacing D^{68} and the new hydrogen bond between Y^{57} and D^{68} in the **13h** complex is indicated as a orange broken line.

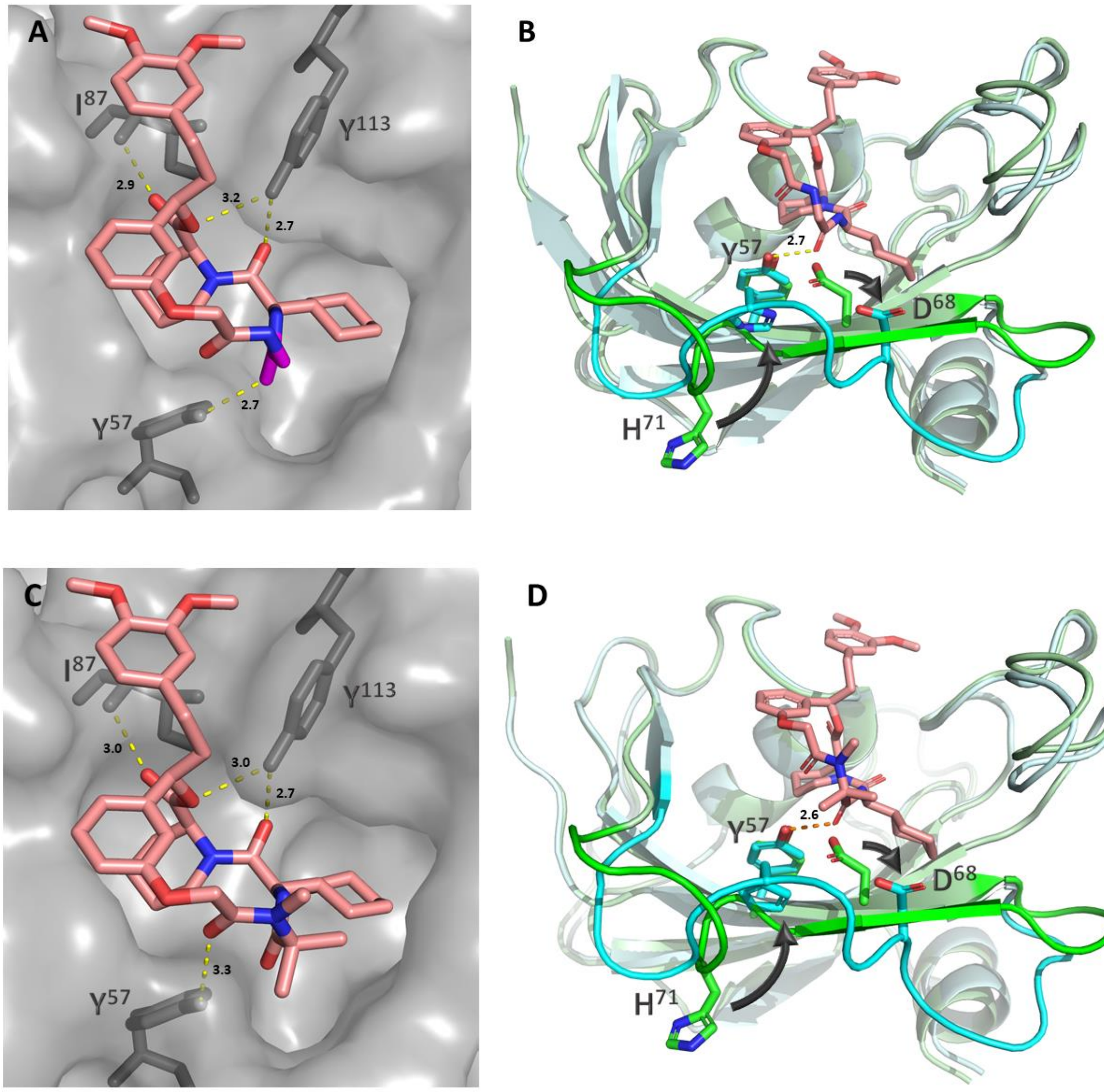

8. Fluorescence Life Time Analysis

Figure S8. Fluorescence life-time analysis of DBD-labeled FKBP51FK1^{K60C} (A) and FKBP51FK1^{K65C} (B) in the presence of the indicated ligands. The chemical structure of the Acyl-DBD dye use for labellling the monoCys constructs is shown in (C) and the structure of the conventional FKBP ligand is shown in (D).

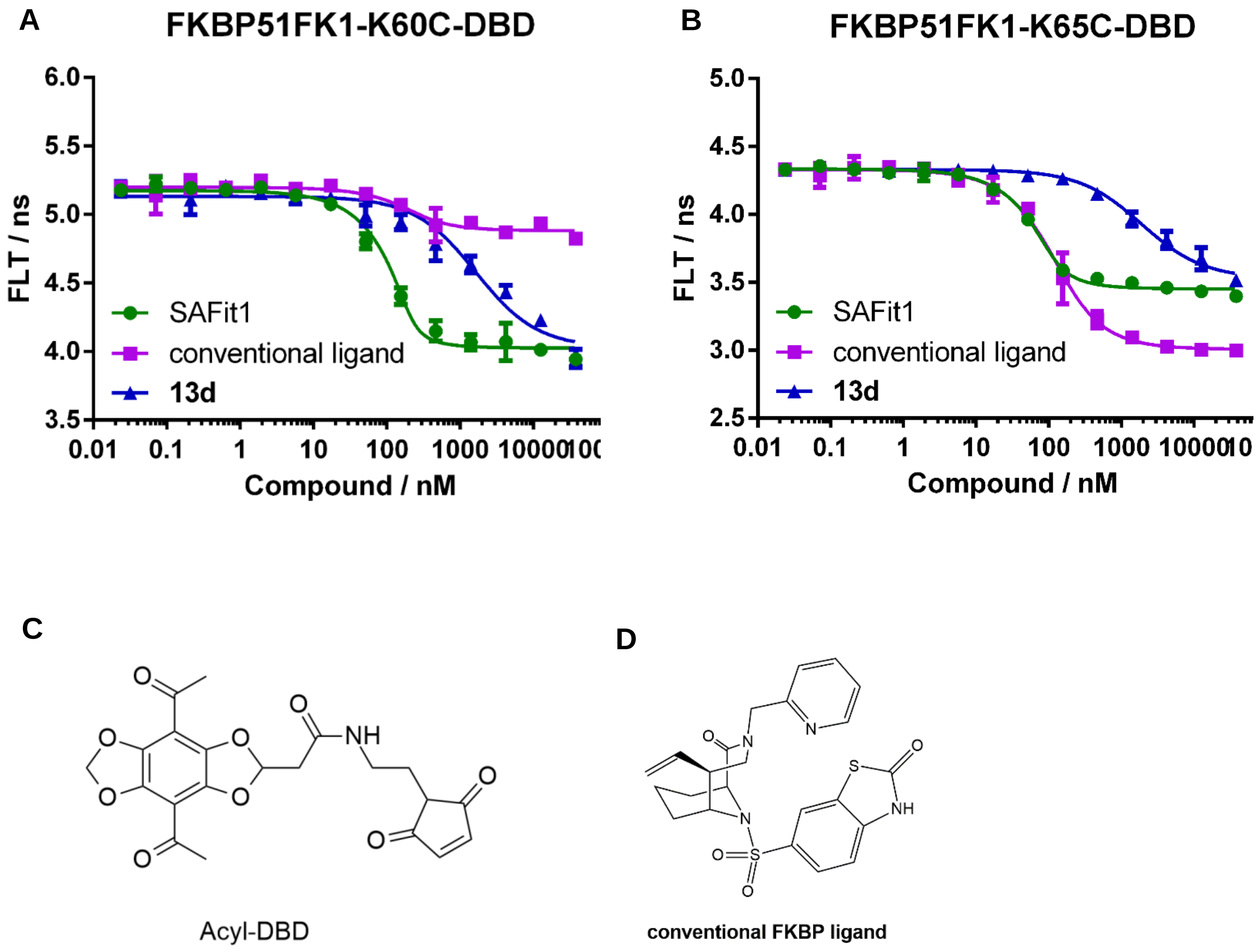

Conformation sensitive fluorescence lifetime assays

For the generation of a conformation sensitive fluorescence lifetime assays mono-cysteine variants of FKBP51FK1 (C103A, C107I, K58C or K60C or K65C) bearing a single cysteine at position 58 or 60 or 65 were labeled with a maleimide functionalized [1,3]dioxolo[4,5-*f*]-[1,3]benzodioxole (DBD)-dye (maleimide **32** from Wawrzinek et al.)[11]. [1,3]dioxolo[4,5-*f*]-[1,3]benzodioxole (DBD)-based dyes have been described as highly sensitive fluorescence lifetime probes which allow to study conformational changes in proteins. For the labeling, the purified protein was immobilized on Ni^{2+}-NTA resin, transferred into coupling buffer (50 mM TRIS-HCl pH 7.2, 20 mM NaCl) and supplemented with a 5-fold molar excess of the maleimido-DBD dye. After an incubation for 3 hours at 25 °C, the unreacted dye was removed by washing the column with 10 column volumes coupling buffer. The protein-dye conjugate was eluted using an imidazole containing buffer (20 mM HEPES-NaOH pH 8.0, 20 mM NaCl, 300 mM imidazole) and dialyzed into 20 mM HEPES-NaOH pH 8.0, 20 mM NaCl. For the conformation sensitive fluorescence lifetime assays a serial dilution of the respective compound in assay buffer (20 mM HEPES-NaOH pH 8.0, 20 mM NaCl, 0.5% DMSO) was placed in a microplate and supplemented with 50-100 nM of the protein-dye conjugate. After equilibration for 30 minutes at 25 °C, the fluorescence lifetime was determined on a LF502 nanoscan plate reader from Berthold Detection Systems GmbH (Pforzheim, Germany) behind a 630 nm band-pass filter after pulse excitation at 456 nm.

9. Compound synthesis

9.1 General information

Reactions

Air and water-sensitive reactions were performed under argon atmosphere and with commercially available dry solvents. All commercially available chemicals and solvents were used as received.

Purification

Chromatographic separations were performed by manual flash chromatography on silica (SiO_2, particle size 4 - 63 µm). Eluents are specified at the respective experiments. Reverse-phase purifications were performed with a Beckman System Gold 126 NMP Programmable Solvent Module fitted with a Phenomenex Jupiter 4µ Proteo 90Å column (250 x 10 mm) and a Beckman System Gold 166 Programmable Detector Module. (Eluent A = 0.1% TFA in H_2O, Eluent B = 0.1% TFA in MeCN).

Analytical HPLC

Analytical HPLC was performed on a Dionex P580 pump with Dionex ASI-100 automated sample injector utilizing a Phenomenex Kinetex 5 µm C18 100 Å column (250 x 4.6 mm) and Dionex UVD 340U Photodiode Array Detector (Eluent A = 0.1% TFA in H_2O, Eluent B = 0.1% TFA in MeCN) or Beckmann System Gold 125S Solvent Module coupled with a Beckman System Gold Diode Array Detector Module 168 and Beckmann Coulter System Gold 508 autosampler utilizing the same column and eluent conditions as described above.

Chiral HPLC

Chiral HPLC was performed on a Beckmann System Gold 125S Solvent Module coupled with a Beckman System Gold Diode Array Detector Module 168 and Beckmann Coulter System Gold 508 autosampler utilizing a normal phase Daicel Chiralcel OD-H column (250 x 4.6 mm, 5 µM) with n-hexane and isopropanol as eluents.

HR-MS

High-resolution mass spectra (HRMS) were obtained by the Mass Spectrometry Department of the Technical University of Darmstadt using a Bruker Daltonics Impact II mass spectrometer (Quadrupole time-of-flight).

LC-MS

Liquid chromatography-mass spectrometry (LC-MS) measurements were performed on a LC-MS system with a Beckmann Coulter System Gold 125 solvent module, Beckmann Coulter System Gold 508 autosampler, Beckman Coulter System 166 Detector connected to a Thermo Finnigan LCQ Deca XP Plus. (Eluent A = 0.1% FA in H_2O, Eluent B = 0.1% FA in MeCN).

NMR

^{1}H NMR spectra, ^{13}C NMR spectra, 2D HSQC, HMBC, COSY and NOESY were obtained from the NMR Department of the Technical University of Darmstadt, on a Bruker DRX 500 or AC/AR300 spectrometer at room temperature. Proton chemical shifts are expressed as parts per million (ppm, δ scale) and are referenced to residual solvent (^{1}H: $CDCl_3$: δ = 7.26, DMSO-d_6: δ = 2.50, THF-d_8: δ = 3.58, ^{13}C: CDCl3: δ = 77.16, DMSO-d_6: δ = 39.52, THF-d_8: δ = 67.57). Coupling constants (*J*) are given in Hertz (Hz).

9.2. Ring closure by metathesis and derivatization (1st macrocycle generation)

3-(3,4-dimethoxyphenyl)-1-(3-hydroxyphenyl)propan-1-one

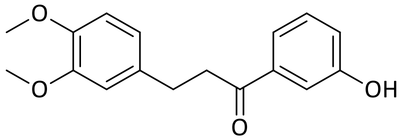

Chemical Formula: $C_{17}H_{18}O_4$

Exact Mass: 286,12

Molecular Weight: 286,32

1

Zn powder (115 g, 1.76 mol, 10.0 eq) and NH_4Cl (94 g, 1.76 mol, 10.0 eq) are added to a flask and suspended in 500 mL MeOH. (*E*)-3-(3,4-dimethoxyphenyl)-1-(3-hydroxyphenyl)prop-2-en-1-one[8] (50 g, 0.18 mol, 1.0 eq) is dissolved in 250 mL MeOH and 180 mL THF and added dropwise to the vigorously stirring suspension over 3 h. After complete addition, the mixture is filtered and washed with MeOH. 1.5 L H_2O is added slowly to the filtrate under stirring to precipitate the product. The mixture is stirred overnight to facilitate full precipitation, then filtered, washed with H_2O and dried on the rotavapor. The pure product **1** is obtained as a beige-white solid.

Yield 41 g (81%).

TLC (CH/EE, 2/1): R*f* (**1**) = 0.34.

^{1}H-NMR (300 MHz, DMSO-*d*$_6$): δ 9.73 (s, 1H), 7.48 – 7.38 (m, 1H), 7.38 – 7.26 (m, 2H), 7.01 (ddd, *J* = 1.1, 2.5, 8.1 Hz, 1H), 6.92 – 6.67 (m, 3H), 3.73 (s, 2H), 3.70 (s, 3H), 3.27 (dd, *J* = 7.0, 8.1 Hz, 2H), 2.86 (t, *J* = 7.5 Hz, 2H).

^{13}C-NMR (75 MHz, DMSO-*d*$_6$): δ 199.13, 157.53, 148.59, 147.02, 138.04, 133.66, 129.70, 120.09, 120.03, 118.85, 114.02, 112.41, 111.87, 55.50, 55.37, 29.22.

LC-MS (30-100% B, 19 min): t*R* (**1**) = 7.67 min, m/z : calculated = 287.12 [M+H]$^+$, found = 287.10 [M+H]$^+$.

RP-HPLC (30 – 100% B, 1.5 mL/min, 20 min, 254 nm): t*R* (**1**) = 7.34 min (99% Purity).

3-(3-(3,4-dimethoxyphenyl)-1-hydroxypropyl)phenol

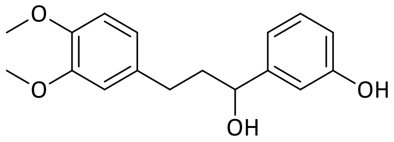

Chemical Formula: $C_{17}H_{20}O_4$
Exact Mass: 288,14
Molecular Weight: 288,34

2_rac

1 (100 mg, 0.35 mmol, 1.0 eq) is dissolved in 5 mL THF. $NaBH_4$ (13.56 mg, 0.35 mmol, 1.0 eq) is added and the reaction stirred at r.t. overnight. The mixture is quenched with 1 mL 3 M HCl. The THF is removed under reduced pressure and the aqueous phase diluted with H_2O, then extracted with DCM (3×10 mL) The crude product is purified by column chromatography (CH/EE, 3/2). The pure product **2_rac** is obtained as a beige-white solid.

Yield 86 mg (85%).

TLC (CH/EE, 3/2): R*f* (**2_rac**) = 0.26.

^{1}H-NMR (300 MHz, DMSO-*d*$_6$): δ 7.13 (td, *J* = 3.5, 7.8 Hz, 1H), 6.86 (t, *J* = 2.0 Hz, 1H), 6.79 (d, *J* = 7.6 Hz, 1H), 6.76 – 6.70 (m, 2H), 6.66 (dt, *J* = 2.3, 6.9 Hz, 2H), 4.58 (dd, *J* = 5.3, 7.7 Hz, 1H), 3.80 (s, 3H), 3.77 (s, 3H), 2.59 (qdd, *J* = 4.1, 6.4, 14.1 Hz, 2H), 2.14 – 1.86 (m, 2H).

^{13}C-NMR (75 MHz, DMSO-*d*$_6$): δ 156.34, 148.83, 147.22, 146.07, 134.41, 129.76, 120.38, 118.19, 114.91, 112.94, 112.06, 111.50, 73.98, 56.01, 55.91, 40.30, 31.63.

LC-MS (30-100% B, 19 min): t*R* (**2_rac**) = 9.05 min, m/z : calculated = 271.12 [M-OH]$^+$, found = 271.06 [M-OH]$^+$.

RP-HPLC (0 – 100% B, 1.5 mL/min, 20 min, 254 nm): t*R* (**2_rac**) = 10.94 min (92% Purity).

Chiral-HPLC (20% iPrOH in n-Hexane, isocratic, 1.0 mL/min, 220 nm): t*R* (**2_rac**) = 16.30 + 18.25 min (*er* = 50.50/49.50).

(R)-3-(3-(3,4-dimethoxyphenyl)-1-hydroxypropyl)phenol

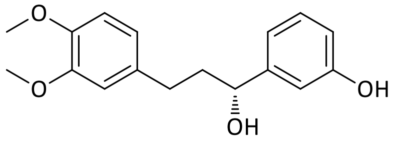

Chemical Formula: $C_{17}H_{20}O_4$
Exact Mass: 288,14
Molecular Weight: 288,34

2_ent

K_2CO_3 (19.30 g, 0.14 mol, 1.0 eq) is filled into an autoclave (Roth, Model II). **1** (40 g, 0.14 mol, 1.0 eq) is dissolved in 150 mL iPrOH and 50 mL THF, then added to the autoclave. The solution is sparged with argon for 10 min, then $RuCl_2$[(*S*)-(DM-SEGPHOS)][(*S*)-DAIPEN] (1.35 g, 1 mmol, 0.008 eq) is added and the autoclave closed, then flushed 3x with H_2, then 10 bar H_2 applied. After 2 d reaction time the mixture is transferred to a beaker and filtered through celite and washed with MeOH. The solvent is removed under

reduced pressure. The crude product is dissolved in EE and 2 g activated charcoal added and stirred for 10 min. Then the mixture is filtered through 500 g silica using a gradient of CH/EE 2/1 going up to CH/EE 1/1. The product fractions are combined, the solvent removed and the brown oily product then dissolved in a minimum amount DCM under heating to reflux. The product is precipitated by cooling the DCM under airstream. After filtering and washing with cooled DCM the pure product **2_ent** is obtained as white solid.

Yield 34.9 g (87%).
TLC (CH/EE, 1/1): R_f(**2_ent**) = 0.35.
^{1}H-NMR (500 MHz, $CDCl_3$): δ 7.15 (td, *J* = 1.5, 7.9 Hz, 1H), 6.86 (t, *J* = 2.0 Hz, 1H), 6.81 (d, *J* = 7.6 Hz, 1H), 6.77 – 6.72 (m, 2H), 6.67 (dt, *J* = 1.8, 10.2 Hz, 2H), 4.59 (t, *J* = 6.6 Hz, 1H), 3.81 (s, 3H), 3.79 (d, *J* = 1.5 Hz, 3H), 2.74 – 2.52 (m, 4H), 2.13 – 1.87 (m, 2H).
^{13}C-NMR (126 MHz, $CDCl_3$): δ 156.28, 148.89, 147.28, 146.16, 134.40, 129.83, 120.39, 118.28, 114.91, 112.94, 112.05, 111.51, 74.00, 56.05, 55.95, 40.38, 31.66.
LC-MS (30-100% B, 19 min): t_R (**2_ent**) = 5.97 min, m/z : calculated = 271.32 $[M-OH]^+$, found = 271.17 $[M-OH]^+$.
RP-HPLC (30 – 100% B, 1.5 mL/min, 20 min, 254 nm): t_R (**2_ent**) = 4.93 min (99% Purity).
Chiral-HPLC (20% iPrOH in n-Hexane, isocratic, 1.0 mL/min, 220 nm): t_R (**2_ent**) = 16.30 + 18.25 min (*er* = 99.33/0.67).

2-(allyloxy)ethyl 4-methylbenzenesulfonate

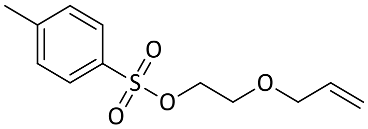

Chemical Formula: $C_{12}H_{16}O_4S$
Exact Mass: 256,08
Molecular Weight: 256,32
3

2-(Allyloxy)ethanol (1.91 g, 0.02 mol, 1 eq) and pyridine (2.96 g, 0.04 mol, 2.0 eq) are dissolved in 30 mL dry DCM and cooled to 0°C for 10 min. Then p-toluenesulfonyl chloride is added portion-wise. The reaction is stirred at slowly rising temperature to r.t. overnight. Complete conversion is checked by TLC and LCMS. The reaction mixture is diluted with 30 mL DCM, washed with 2 M HCl (2 × 30 mL), 4% $NaHCO_3$ (1 × 40 mL), then H_2O (1 × 40 mL) and brine (1 × 50 mL). The organic phase is dried with $MgSO_4$, filtered and the solvent removed under reduced pressure. The crude product is purified by manual column chromatography (CH/EE, 5/1). Product **3** is obtained as slightly yellow oil.

Yield: 2.72 g (57%).
TLC (CH/EE, 5/1, v/v): R_f (**3**) = 0.25.
^{1}H-NMR (500 MHz, $CDCl_3$): δ 7.82 – 7.77 (m, 2H), 7.36 – 7.31 (m, 2H), 5.81 (ddt, J = 5.5, 10.3, 17.2 Hz, 1H), 5.22 (dq, J = 1.6, 17.3 Hz, 1H), 5.16 (dq, J = 1.4, 10.4 Hz, 1H), 4.17 (ddd, J = 0.9, 3.7, 5.8 Hz, 2H), 3.94 (dt, J = 1.4, 5.5 Hz, 2H), 3.65 – 3.59 (m, 2H), 2.44 (s, 3H), 1.57 (d, J = 1.7 Hz, 0H).
^{13}C-NMR (126 MHz, $CDCl_3$): δ 144.90, 134.26, 133.27, 129.94, 128.12, 117.53, 72.31, 69.37, 67.59, 21.76.
LC-MS (0-100% B, 19 min): t_R (**3**) = 11.4 min, m/z : calculated = 257.08 $[M+H]^+$, found = 257.09 $[M+H]^+$.

(R)-1-(3-(allyloxy)phenyl)-3-(3,4-dimethoxyphenyl)propan-1-ol

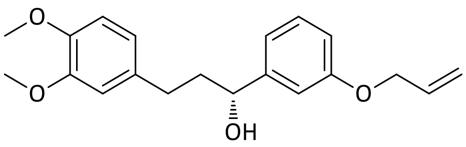

Chemical Formula: $C_{20}H_{24}O_4$
Exact Mass: 328,17
Molecular Weight: 328,40

4a

2_ent (2.16 g, 7.49 mmol, 1.0 eq), K_2CO_3 (1.14 g, 8.24 mmol, 1.1 eq) and allyl bromide (997 mg, 8.24 mmol, 1.1 eq) are dissolved in 70 mL MeCN. The reaction is stirred overnight at r.t. After complete conversion, the mixture is filtered over celite and washed with MeCN. The solvent is removed under reduced pressure and the crude product purified by silica filtration (CH/EE, 3/2). Compound **4a** is obtained as a white solid.

Yield 2.31 g (94%).
TLC (CH/EE, 3/2, v/v): R_f(**4a**) = 0.53.
^{1}H-NMR (300 MHz, CDCl$_3$): δ 7.24 (ddd, *J* = 1.0, 7.0, 8.2 Hz, 1H), 6.91 (dd, *J* = 1.4, 7.3 Hz, 2H), 6.86 – 6.78 (m, 1H), 6.77 (d, *J* = 0.8 Hz, 1H), 6.73 (d, *J* = 1.8 Hz, 1H), 6.71 (s, 1H), 6.13 – 5.98 (m, 1H), 5.41 (dq, *J* = 1.6, 17.3 Hz, 1H), 5.28 (dq, *J* = 1.4, 10.5 Hz, 1H), 4.64 (dd, *J* = 5.3, 7.7 Hz, 1H), 4.52 (dt, *J* = 1.5, 5.2 Hz, 2H), 3.84 (d, *J* = 1.5 Hz, 6H), 2.65 (qdd, *J* = 6.4, 9.2, 13.8 Hz, 2H), 2.20 – 1.91 (m, 2H).
^{13}C-NMR (75 MHz, CDCl$_3$): δ 158.81, 148.86, 147.21, 146.44, 134.45, 133.29, 129.53, 120.25, 118.46, 117.70, 113.76, 112.38, 111.83, 111.32, 73.77, 68.80, 55.96, 55.85, 40.63, 31.68.
LC-MS (0-100%, 19 min): t_R (**4a**) = 10.47 min, m/z : calculated = 311.17 $[M-OH]^+$, found = 311.03 $[M-OH]^+$.
RP-HPLC (0 – 100% B, 1.5 mL/min, 20 min, 254 nm): t_R (**4a**) = 14.75 min (97% Purity).

(R)-1-(3-(2-(allyloxy)ethoxy)phenyl)-3-(3,4-dimethoxyphenyl)propan-1-ol

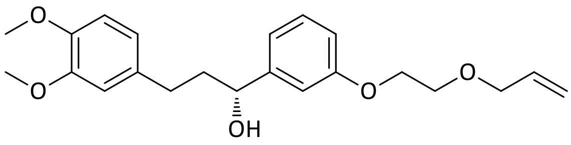

Chemical Formula: $C_{22}H_{28}O_5$
Exact Mass: 372,19
Molecular Weight: 372,45

4b

2_ent (969 mg, 3.36 mmol, 1.0 eq), K_2CO_3 (697 g, 5 mmol, 1.5 eq) and **3** (861 mg, 3.4 mmol, 1.1 eq) are dissolved in 20 mL MeCN. The reaction is stirred overnight after heating to reflux. After complete conversion, the mixture is filtered over celite and washed with acetone. The solvent is removed under reduced pressure and the crude product purified by column chromatography (CH/EE, 3/1). Compound **4b** is obtained as a beige oil.

Yield 1.18 g (94%).
TLC (CH/EE, 1/1, v/v): R_f(**4b**) = 0.46.
^{1}H-NMR (300 MHz, CDCl$_3$): δ 7.26 (td, *J* = 2.8, 7.9 Hz, 1H), 6.99 – 6.90 (m, 2H), 6.89 – 6.77 (m, 1H), 6.77 – 6.66 (m, 2H), 5.95 (ddt, *J* = 5.0, 10.1, 16.2 Hz, 1H), 5.32 (dd, *J* = 2.7, 17.2 Hz, 1H), 5.22 (d, *J* = 10.3 Hz, 1H), 4.72 – 4.60 (m, 1H), 4.19 – 4.03 (m, 5H), 3.86 (d, *J* = 2.9 Hz, 6H), 3.84 – 3.72 (m, 2H), 2.66 (qdd, *J* = 2.7, 8.4, 13.8, 16.1 Hz, 2H), 2.13 – 1.92 (m, 3H).
^{13}C-NMR (75 MHz, CDCl$_3$): δ 159.06, 148.91, 147.25, 146.45, 134.61, 134.50, 129.51, 120.26, 118.51, 117.37, 113.66, 112.33, 111.90, 111.40, 73.77, 72.41, 68.57, 67.47, 55.99, 55.88, 40.65, 31.67, 26.97.
LC-MS (30-100%, 19 min): t_R (**4b**) = 10.93 min, m/z : calculated = 354.17 $[M-OH]^+$, 390.22 $[M+NH_4]^+$, found = 355.10 $[M-OH]^+$, 390.04 $[M+NH_4]^+$.
RP-HPLC (0 – 100% B, 1.5 mL/min, 20 min, 220 nm): t_R (**4b**) = 14.54 min (93% Purity).

(S)-1-((9H-fluoren-9-yl)methyl) 2-((R)-1-(3-(allyloxy)phenyl)-3-(3,4-dimethoxyphenyl)propyl) piperidine-1,2-dicarboxylate

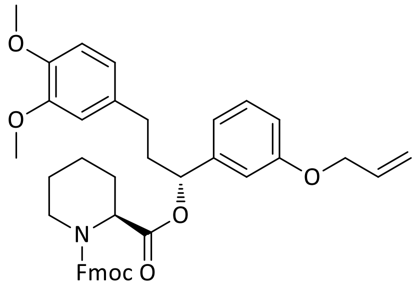

Chemical Formula: $C_{41}H_{43}NO_7$
Exact Mass: 661,30
Molecular Weight: 661,78

5a

4a (1.29 g, 3.47 mmol, 1.0 eq) and Fmoc-*S*-pipecolate (1.99 g, 5.66 mmol, 1.1 eq) are dissolved in 40 mL dry DCM and cooled to 0°C for 15 min. DMAP (70 mg, 0.57 mmol, 0.1 eq) is added and stirred until dissolved, then DCC (1.17 g, 5.66 mmol, 1.1 eq) is added. The mixture is stirred for 15 min under cooling. Finally, the ice bath is removed and the reaction stirred overnight at r.t. The reaction mixture is filtered, washed with DCM and the solvent removed under reduced pressure. The crude product is purified by silica column chromatography (CH/EE, 5/1) and pure product **5a** obtained as white foam.

Yield 3.16 g (93%).
TLC (CH/EE, 5/1, v/v): R_f (**5a**) = 0.22.
LC-MS (70-100% B, 19 min): t_R (**5a**) = 7.70 min, m/z : calculated = 679.30 $[M+H]^+$, found = 679.02 $[M+H]^+$.
RP-HPLC (70 – 100% B, 1.5 mL/min, 20 min, 220 nm): t_R (**5a**) = 11.12 min (99% Purity).

(S)-1-((9H-fluoren-9-yl)methyl) 2-((R)-1-(3-(2-(allyloxy)ethoxy)phenyl)-3-(3,4-dimethoxyphenyl)propyl) piperidine-1,2-dicarboxylate

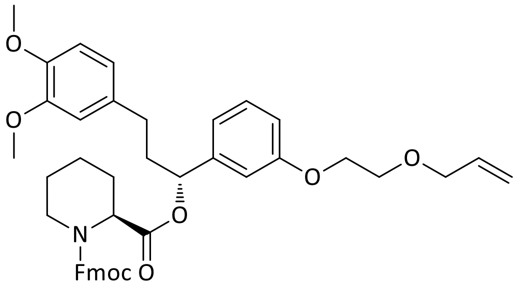

Chemical Formula: $C_{43}H_{47}NO_8$
Exact Mass: 705,33
Molecular Weight: 705,84

5b

4b (1.29 g, 3.47 mmol, 1.0 eq) and Fmoc-*S*-pipecolate (1.30 g, 3.81 mmol, 1.1 eq) are dissolved in 20 mL dry DCM and cooled to 0°C for 15 min. DMAP (50 mg, 0.38 mmol, 0.1 eq) is added and stirred until dissolved, then DCC (0.8 g, 3.81 mmol, 1.1 eq) is added. The mixture is stirred for 15 min under cooling. Finally, the ice bath is removed and the reaction stirred overnight at r.t. The reaction mixture is filtered, washed with DCM and the solvent removed under reduced pressure. The crude product is purified by silica column chromatography (CH/EE, 4/1) and pure product **5b** obtained as white foam.

Yield 2.17 g (89%).
TLC (CH/EE, 4/1, v/v): R_f (**5b**) = 0.22.
LC-MS (50-100% B, 19 min): t_R (**5b**) = 12.27 min, m/z : calculated = 723.36 $[M+NH_4]^+$, found = 723.07 $[M+NH_4]^+$.
RP-HPLC (50 – 100% B, 1.5 mL/min, 20 min, 220 nm): t_R (**5b**) = 16.82 min (99% Purity).

(S)-(R)-1-(3-(allyloxy)phenyl)-3-(3,4-dimethoxyphenyl)propyl piperidine-2-carboxylate

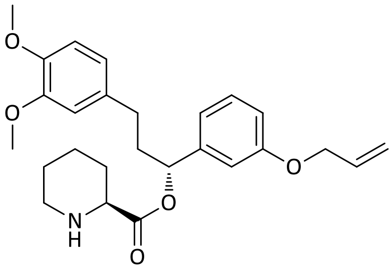

Chemical Formula: $C_{26}H_{33}NO_5$
Exact Mass: 439,24
Molecular Weight: 439,54

5.1a

5a (3.15 g, 4.76 mmol, 1.0 eq) is dissolved in 40 mL DCM and 10% 4-methylpiperidine (4.4 mL) is added. The reaction is stirred at r.t. for 2 h, then diluted with 50 mL DCM, washed with 1 M HCl (4x50 mL) and brine (1x50 mL). The organic phase is dried with $MgSO_4$, filtered and the solvent removed. The crude product is purified by silica column chromatography (CH/EE, 2/1 +1% TEA + 2% MeOH) and the pure product **5.1a** obtained as colorless oil.

Yield 1.85 g (89%).
TLC (CH/EE, 2/1 +1% TEA + 2% MeOH, v/v): R_f (**5.1a**) = 0.25.
^{1}H-NMR (300 MHz, Chloroform-*d*): δ 7.24 (t, J = 7.8 Hz, 1H), 6.96 – 6.85 (m, 2H), 6.83 (ddd, J = 1.0, 2.6, 8.3 Hz, 1H), 6.78 (d, J = 7.9 Hz, 1H), 6.67 (d, J = 7.9 Hz, 2H), 6.05 (ddt, J = 5.3, 10.5, 17.1 Hz, 1H), 5.77 (dd, J = 5.7, 7.9 Hz, 1H), 5.41 (dq, J = 1.6, 17.2 Hz, 1H), 5.28 (dq, J = 1.4, 10.5 Hz, 1H), 4.52 (dt, J = 1.5, 5.3 Hz, 2H), 3.85 (s, 3H), 3.84 (s, 3H), 3.37 (dd, J = 3.2, 9.7 Hz, 1H), 3.07 (dt, J = 3.4, 11.9 Hz, 1H), 2.72 – 2.45 (m, 3H), 2.34 – 2.15 (m, 1H), 2.15 – 1.96 (m, 3H), 1.87 – 1.74 (m, 1H), 1.70 – 1.37 (m, 4H).

^{13}C-NMR (75 MHz, Chloroform-*d*): δ 172.95, 158.83, 149.03, 147.47, 141.94, 133.77, 133.31, 129.65, 120.27, 119.15, 117.83, 114.07, 113.34, 111.84, 111.47, 75.64, 68.94, 58.87, 56.05, 55.98, 45.78, 38.07, 31.47, 29.41, 25.97, 24.27.

LC-MS (0-100% B, 19 min): t_R (**5.1a**) = 8.32 min, m/z : calculated = 440.24 $[M+H]^+$, found = 440.23 $[M+H]^+$.

RP-HPLC (0 – 100% B, 1.5 mL/min, 20 min, 220 nm): t_R (**5.1a**) = 12.95 min (93% Purity).

((S)-(R)-1-(3-(2-(allyloxy)ethoxy)phenyl)-3-(3,4-dimethoxyphenyl)propyl piperidine-2-carboxylate

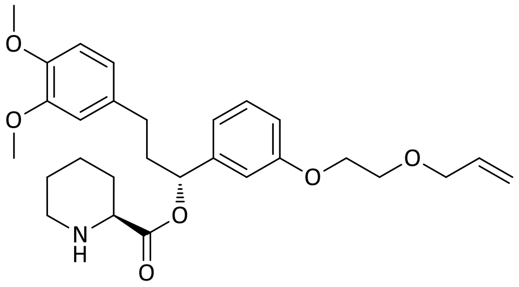

Chemical Formula: $C_{28}H_{37}NO_6$
Exact Mass: 483,26
Molecular Weight: 483,60

5.1b

5b (2.17 g, 3.08 mmol, 1.0 eq) is dissolved in 30 mL DCM and 10% 4-methylpiperidine (3.3 mL) is added. The reaction is stirred at r.t. for 2 h, then diluted with 50 mL DCM, washed with 1 M HCl (4x50 mL) and brine (1x50 mL). The organic phase is dried with $MgSO_4$, filtered and the solvent removed. The crude product is purified by silica column chromatography (CH/EE, 2/1 +1% TEA + 2% MeOH) and the pure product **5.1b** obtained as colorless oil.

Yield 1.23 g (83%).

TLC (CH/EE, 2/1 +1% TEA + 2% MeOH, v/v): R_f (**5.1b**) = 0.25.

^{1}H-NMR (300 MHz, Chloroform-*d*): δ 7.23 (t, J = 8.0 Hz, 1H), 6.90 (dt, J = 1.2, 5.6 Hz, 2H), 6.83 (ddd, J = 1.0, 2.6, 8.2 Hz, 1H), 6.77 (d, J = 7.9 Hz, 1H), 6.66 (d, J = 7.8 Hz, 2H), 5.93 (ddt, J = 5.7, 10.4, 17.3 Hz, 1H), 5.76 (dd, J = 5.7, 7.9 Hz, 1H), 5.37 – 5.25 (m, 1H), 5.20 (dq, J = 1.4, 10.4 Hz, 1H), 4.16 – 4.05 (m, 4H), 3.85 (s, 3H), 3.84 (s, 3H), 3.82 – 3.74 (m, 2H), 3.36 (dd, J = 3.2, 9.7 Hz, 1H), 3.11 – 3.00 (m, 1H), 2.70 – 2.44 (m, 3H), 2.31 – 2.16 (m, 1H), 2.12 – 1.96 (m, 4H), 1.80 (dt, J = 3.8, 7.8 Hz, 1H), 1.69 – 1.36 (m, 3H).

^{13}C-NMR (75 MHz, Chloroform-*d*): δ 172.81, 158.92, 148.89, 147.33, 141.78, 134.53, 133.65, 129.49, 120.14, 119.08, 117.33, 113.77, 113.19, 111.71, 111.34, 75.51, 72.37, 68.49, 67.42, 58.73, 55.92, 55.86, 45.66, 37.94, 31.33, 29.28, 25.84, 24.15.

LC-MS (0-100% B, 19 min): t_R (**5.1b**) = 8.65 min, m/z : calculated = 484.26 $[M+H]^+$, found = 484.18 $[M+H]^+$.

RP-HPLC (0 – 100% B, 1.5 mL/min, 20 min, 220 nm): t_R (**5.1b**) = 12.97 min (98% Purity).

(S)-(R)-1-(3-(allyloxy)phenyl)-3-(3,4-dimethoxyphenyl)propyl 1-((R)-2-cyclohexylpent-4-enoyl)piperidine-2-carboxylate

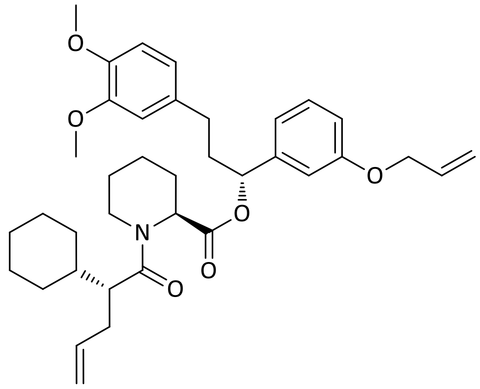

Chemical Formula: $C_{37}H_{49}NO_6$
Exact Mass: 603,36
Molecular Weight: 603,79

7a

(*R*)-2-cyclohexylpent-4-enoic acid (**6**)[9] (456 mg, 2.50 mmol, 1.1 eq) is dissolved in 5 mL DMF, HATU (951 mg, 2.50 mmol, 1.1 eq) and DIPEA (1160 µL, 6.83 mmol, 3.0 eq) are added and the mixture stirred for 5 min. Then a solution of **5.1a** (1.0 g, 2.28 mmol, 1.0 eq) in 15 mL DMF is added. The reaction is stirred at r.t overnight. The solvent is removed under reduced pressure. The crude product is purified by silica column chromatography (CH/EE, 7/1) to obtain pure product **7a** as a sticky resin.

Yield 900 mg (68%).
TLC (CH/EE, 7/1, v/v): R_f (**7a**) = 0.21.
^{1}H-NMR (300 MHz, Chloroform-*d*): δ 7.27 – 7.19 (m, 1H), 6.93 – 6.81 (m, 3H), 6.80 – 6.73 (m, 1H), 6.71 – 6.62 (m, 2H), 6.11 – 5.98 (m, 1H), 5.93 – 5.81 (m, 1H), 5.81 – 5.72 (m, 1H), 5.64 – 5.53 (m, 1H), 5.46 – 5.36 (m, 1H), 5.32 – 5.24 (m, 2H), 5.10 – 4.96 (m, 1H), 4.96 – 4.88 (m, 1H), 4.56 – 4.49 (m, 2H), 3.98 – 3.87 (m, 1H), 3.84 (d, J = 2.2 Hz, 6H), 3.15 – 3.01 (m, 1H), 2.66 – 2.39 (m, 3H), 2.38 – 2.17 (m, 3H), 2.13 – 1.94 (m, 1H), 1.87 (d, J = 13.2 Hz, 1H), 1.80 – 1.48 (m, 9H), 1.46 – 1.04 (m, 4H), 1.03 – 0.70 (m, 2H).
^{13}C-NMR (75 MHz, Chloroform-*d*): δ 174.76, 170.86, 170.43, 158.77, 148.96, 148.89, 147.33, 141.71, 141.34, 136.81, 136.40, 133.68, 133.37, 133.26, 133.13, 129.74, 129.63, 120.24, 120.16, 119.30, 119.15, 117.84, 117.73, 116.75, 116.07, 114.37, 114.26, 113.38, 113.27, 111.76, 111.67, 111.33, 76.21, 68.85, 56.33, 55.98, 55.88, 51.97, 47.47, 46.88, 43.84, 40.38, 40.13, 38.09, 37.77, 34.80, 34.53, 32.02, 31.74, 31.50, 31.35, 30.46, 30.20, 27.10, 26.54, 26.47, 26.44, 26.21, 25.72, 21.25, 21.16
LC-MS (70-100% B, 19 min): t_R (**7a**) = 8.48 min, m/z : calculated = 604.36 $[M+H]^+$, found = 604.13 $[M+H]^+$.
RP-HPLC (0 – 100% B, 1.5 mL/min, 20 min, 254 nm): t_R (**7a**) = 17.15 min (98% Purity).

(S)-(R)-1-(3-(2-(allyloxy)ethoxy)phenyl)-3-(3,4-dimethoxyphenyl)propyl 1-((R)-2-cyclohexylpent-4-enoyl)piperidine-2-carboxylate

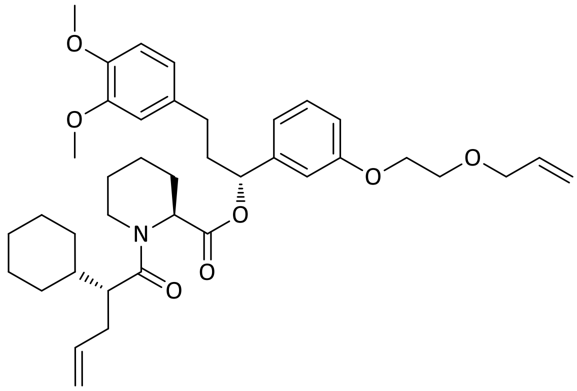

Chemical Formula: $C_{39}H_{53}NO_7$
Exact Mass: 647,38
Molecular Weight: 647,84

7b

(*R*)-2-cyclohexylpent-4-enoic acid (**6**)[9] (415 mg, 2.27 mmol, 1.1 eq) is dissolved in 5 mL DMF, HATU (865 mg, 2.27 mmol, 1.1 eq) and DIPEA (1055 µL, 6.20 mmol, 3.0 eq) are added and the mixture stirred for 5 min. Then a solution of **5.1b** (1.0 g, 2.07 mmol, 1.0 eq) in 10 mL DMF is added. The reaction is stirred at r.t overnight. The solvent is removed under reduced pressure. The crude product is purified by silica column chromatography (CH/EE, 7/1) to obtain pure product **7b** as a sticky resin.

Yield 1.20 g (90%).
TLC (CH/EE, 7/1, v/v): R_f (**7b**) = 0.23.
^{1}H-NMR (300 MHz, Chloroform-*d*): δ 7.23 (t, J = 7.4, 8.3 Hz, 1H), 6.93 – 6.87 (m, 2H), 6.86 – 6.80 (m, 1H), 6.80 – 6.73 (m, 1H), 6.69 – 6.61 (m, 2H), 6.01 – 5.86 (m, 1H), 5.85 – 5.71 (m, 2H), 5.59 (d, J = 5.7 Hz, 1H), 5.35 – 5.24 (m, 1H), 5.24 – 5.15 (m, 1H), 5.09 – 4.95 (m, 1H), 4.95 – 4.87 (m, 1H), 4.15 – 4.03 (m, 6H), 3.95 – 3.88 (m, 1H), 3.86 – 3.81 (m, 6H), 3.81 – 3.72 (m, 2H), 3.14 – 3.01 (m, 1H), 2.74 – 2.13 (m, 7H), 1.93 – 1.79 (m, 1H), 1.79 – 1.50 (m, 9H), 1.42 – 1.01 (m, 4H), 1.01 – 0.66 (m, 2H).
^{13}C-NMR (75 MHz, Chloroform-*d*): δ 174.75, 174.46, 170.83, 170.40, 158.97, 148.95, 148.88, 147.45, 147.31, 141.64, 141.28, 136.80, 136.39, 134.62, 134.57, 133.67, 133.37, 129.71, 129.60, 120.22, 120.14, 119.34, 119.18, 117.47, 117.41, 116.74, 116.06, 114.18, 114.13, 113.35, 113.22, 111.75, 111.65, 111.31, 76.22, 72.44, 68.56, 68.52, 67.49, 60.45, 56.33, 55.97, 55.88, 51.96, 47.44, 46.86, 43.83, 40.37, 40.12, 39.23, 38.06, 37.77, 34.79, 34.52, 32.01, 31.73, 31.47, 31.33, 30.45, 30.19, 29.78, 28.29, 27.10, 26.53, 26.47, 26.43, 26.32, 26.21, 25.71, 25.12, 21.24, 21.13, 14.28.
LC-MS (70-100% B, 19 min): t_R (**7b**) = 8.42 min, m/z : calculated = 648.38 $[M+H]^+$, found = 648.06 $[M+H]^+$.
RP-HPLC (70 – 100% B, 1.5 mL/min, 20 min, 254 nm): t_R (**7b**) = 9.99 min (99% Purity).

(2R,5S,12R,14Z)-12-cyclohexyl-2-[2-(3,4-dimethoxyphenyl)ethyl]-3,17-dioxa-10-azatricyclo[16.3.1.0^{5,10}]docosa-1(22),14,18,20-tetraene-4,11-dione

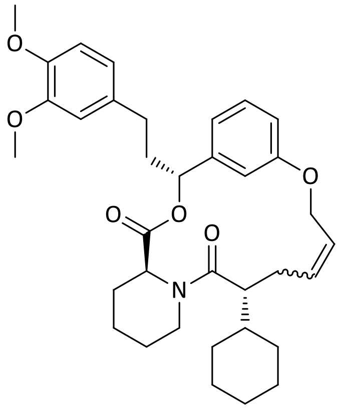

Chemical Formula: $C_{35}H_{45}NO_6$
Exact Mass: 575,32
Molecular Weight: 575,73

8aE (E-isomer) and **Z** (Z-isomer)

7a (536 mg, 0.89 mmol, 1.0 eq) is dissolved in 1.8 L dry DCM (0.5 mM) in a dried flask with condenser. The solution is sparged continuously with argon and heated to 30°C. The system is equilibrated for 45 min, then Grubbs 2nd generation catalyst (75 mg, 0.089, 0.1 eq) is added. After 2.5 h the solution is filtered through a silica plug and the product eluted with 300 mL EE. The solvent is removed under reduced pressure and the crude mixture purified by manual silica column chromatography (CH/EE, 4/1) to obtain pure products **8aE** (E-isomer) and **Z** (Z-isomer).

Yield (8a) 441 mg (86%), (**8aE**) 44 mg (9%).

TLC (CH/EE, 4/1, v/v): R_f(**8aE**) = 0.25, R_f(**8aZ**) = 0.35.

^{1}H-NMR (500 MHz, THF-d$_8$, 8aE): δ 7.10 (t, J = 7.3 Hz, 1H), 7.05 – 7.01 (m, 1H), 6.80 – 6.73 (m, 1H), 6.75 (d, J = 8.1 Hz, 1H), 6.72 (d, J = 2.0 Hz, 1H), 6.72 – 6.66 (m, 1H), 6.62 (dd, J = 2.1, 8.1 Hz, 1H), 5.91 (dt, J = 6.6, 8.0, 15.5 Hz, 1H), 5.64 (dd, J = 4.9, 8.3 Hz, 1H), 5.64 – 5.58 (m, 1H), 5.56 (ddd, J = 4.7, 7.4, 15.7 Hz, 1H), 4.64 – 4.49 (m, 2H), 3.73 (d, J = 13.1 Hz, 6H), 3.68 – 3.60 (m, 1H), 2.57 – 2.30 (m, 6H), 2.27 – 2.19 (m, 1H), 2.12 – 2.03 (m, 1H), 2.04 – 1.93 (m, 1H), 1.80 – 1.66 (m, 4H), 1.67 – 1.54 (m, 4H), 1.52 – 1.45 (m, 1H), 1.44 – 1.33 (m, 1H), 1.32 – 1.24 (m, 1H), 1.24 – 1.09 (m, 3H), 1.07 – 0.96 (m, 1H), 0.92 – 0.83 (m, 1H).

^{13}C-NMR (126 MHz, THF-d$_8$, 8aE): δ 172.35, 171.44, 160.69, 150.91, 149.26, 142.09, 135.88, 134.76, 129.51, 127.89, 121.12, 120.34, 118.78, 113.71, 113.41, 111.32, 78.16, 69.15, 56.46, 56.29, 52.35, 48.51, 44.24, 42.47, 38.51, 32.61, 31.86, 31.26, 29.76, 28.06, 27.87, 27.49, 26.88, 26.84, 21.75.

^{1}H-NMR (500 MHz, THF-d$_8$, 8aZ): δ 7.11 (t, J = 7.9 Hz, 1H), 6.79 – 6.75 (m, 2H), 6.74 (d, J = 2.0 Hz, 1H), 6.70 (d, 1H), 6.69 – 6.65 (m, 2H), 5.73 (dd, J = 3.7, 8.5 Hz, 1H), 5.49 – 5.44 (m, 1H), 5.36 – 5.28 (m, 1H), 5.23 (dt, J = 2.1, 4.0, 11.3 Hz, 1H), 5.10 (dt, J = 2.6, 4.3, 11.0 Hz, 1H), 4.63 (dq, J = 2.8, 16.0 Hz, 1H), 3.98 – 3.91 (m, 1H), 3.75 (s, 3H), 3.72 (s, 3H), 3.50 (td, J = 3.0, 13.0 Hz, 1H), 2.76 – 2.68 (m, 1H), 2.66 – 2.60 (m, 2H), 2.56 – 2.45 (m, 1H), 2.29 – 2.17 (m, 1H), 2.17 – 2.10 (m, 1H), 2.10 – 2.00 (m, 2H), 1.90 – 1.82 (m, 1H), 1.80 – 1.62 (m, 10H), 1.62 – 1.41 (m, 1H), 1.34 – 0.95 (m, 4H).

^{13}C-NMR (126 MHz, THF-d$_8$, 8aZ): δ 176.07, 172.84, 159.27, 150.98, 149.33, 142.82, 135.20, 131.01, 129.76, 129.14, 121.20, 118.07, 117.87, 113.91, 113.53, 110.81, 75.45, 65.21, 56.47, 56.34, 52.22, 47.35, 44.10, 42.28, 39.03, 32.42, 32.05, 31.25, 30.06, 27.86, 27.66, 27.57, 27.54, 26.58, 20.76.

LC-MS (50-100% B, 19 min): t_R (**8aE**) = 11.75 min, m/z : calculated = 576.32 [M+H]$^+$, found = 576.05 [M+H]$^+$, t_R (**8aZ**) = 11.94 min, m/z : calculated = 576.32 [M+H]$^+$, found = 576.35 [M+H]$^+$.

HRMS (ESI, 8aE): calculated = 576.33196 [M+H]$^+$, found = 576.33201 [M+H]$^+$, err [ppm] = 0.08.

HRMS (ESI, 8aZ): calculated = 576.33196 [M+H]$^+$, found = 576.33236 [M+H]$^+$, err [ppm] = 0.69.

RP-HPLC (50 – 100% B, 1.5 mL/min, 20 min, 254 nm): t_R (**8aE**) = 15.78 min (97% Purity).

RP-HPLC (50 – 100% B, 1.5 mL/min, 20 min, 254 nm): t_R (**8aZ**) = 16.48 min (98% Purity).

(2R,5S,12R,14E)-12-cyclohexyl-2-[2-(3,4-dimethoxyphenyl)ethyl]-3,17,20-trioxa-10-azatricyclo[19.3.1.0^{5,10}]pentacosa-1(25),14,21,23-tetraene-4,11-dione

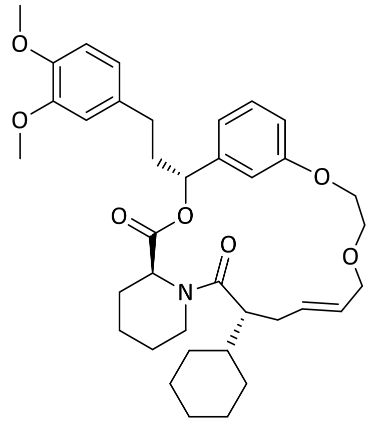

Chemical Formula: $C_{36}H_{49}NO_7$
Exact Mass: 607,35
Molecular Weight: 607,78

8b

7b (592 mg, 0.91 mmol, 1.0 eq) is dissolved in 1.8 L dry DCM (0.5 mM) in a dried flask with condenser. The solution is sparged continuously with argon and heated to 30°C. The system is equilibrated for 45 min, then Grubbs 2nd generation catalyst (78 mg, 0.091, 0.1 eq) is added. After 2.5 h the solution is filtered through a silica plug and the product eluted with 200 mL EE. The solvent is removed under reduced pressure and the crude mixture purified by manual silica column chromatography (CH/EE, 4/1) to obtain pure product **8b**.

Yield 519 mg (92%).
TLC (CH/EE, 3/1, v/v): R_f (**8b**) = 0.25.
^{1}H-NMR (500 MHz, CD_2Cl_2): δ 7.28 – 7.19 (m, 1H), 7.11 (t, J = 2.1 Hz, 1H), 6.94 – 6.90 (m, 1H), 6.85 (ddd, J = 0.9, 2.5, 8.2 Hz, 1H), 6.82 – 6.77 (m, 1H), 6.75 – 6.68 (m, 2H), 5.69 (dd, J = 5.1, 8.8 Hz, 1H), 5.64 (ddd, J = 1.3, 5.8, 15.0 Hz, 1H), 5.59 (d, J = 5.9 Hz, 0H), 5.52 (dt, J = 5.7, 15.4 Hz, 1H), 5.47 (d, 1H), 5.44 – 5.36 (m, 0H), 4.23 – 4.18 (m, 2H), 3.90 (d, 2H), 3.81 (d, J = 5.3 Hz, 6H), 3.72 – 3.57 (m, 2H), 3.18 (td, J = 3.1, 12.8 Hz, 1H), 2.71 – 2.63 (m, 1H), 2.63 – 2.52 (m, 2H), 2.47 – 2.40 (m, 1H), 2.31 – 2.21 (m, 3H), 2.14 – 2.03 (m, 1H), 1.87 – 1.81 (m, 1H), 1.79 – 1.53 (m, 8H), 1.48 – 1.35 (m, 2H), 1.31 – 1.20 (m, 3H), 1.20 – 1.08 (m, 1H), 1.04 – 0.91 (m, 2H).
^{13}C-NMR (126 MHz, CD_2Cl_2): δ 175.05, 171.89, 159.50, 149.76, 148.15, 142.51, 134.50, 132.19, 129.94, 128.32, 120.82, 120.12, 116.69, 115.33, 112.77, 112.32, 76.58, 71.79, 69.78, 68.93, 56.42, 56.34, 56.30, 52.49, 47.49, 44.22, 40.84, 38.32, 32.93, 32.30, 31.89, 30.55, 27.41, 27.10, 27.02, 26.14, 21.43.
LC-MS (50-100% B, 19 min): t_R (**8b**) = 10.67 min, m/z : calculated = 620.35 $[M+H]^+$, found = 620.39 $[M+H]^+$.
HRMS (ESI): calculated = 620.35818 $[M+H]^+$, found = 620.35870 $[M+H]^+$, err [ppm] = 0.84.
RP-HPLC (50 – 100% B, 1.5 mL/min, 20 min, 254 nm): t_R (**8b**) = 14.72 min (98% Purity).

(2R,5S,12R)-12-cyclohexyl-2-[2-(3,4-dimethoxyphenyl)ethyl]-3,17-dioxa-10-azatricyclo[16.3.1.0^{5,10}]docosa-1(22),18,20-triene-4,11-dione

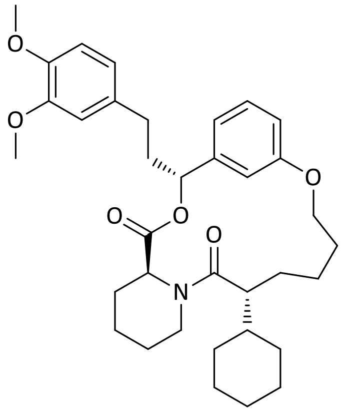

Chemical Formula: $C_{35}H_{47}NO_6$
Exact Mass: 577,34
Molecular Weight: 577,75

9a

8aE (48 mg, 0.083 mmol, 1.0 eq) is dissolved in 3 mL dry MeOH and the solvent sparged with argon for 5 min, Then Pt/C (2 mg, 0.0008 mmol, 0.01 eq) is added and the slurry sparged with H_2 for 10 min. Then the reaction is stirred at 1 bar H_2 for 2 h. The mixture is filtered over SiO_2 and eluted with MeOH. The solvent is removed and the crude product purified by semi-preparative HPLC.

prep-HPLC (70 – 100% B, 10 mL/min, 10 min, 254 nm): t_R (**9a**) = 6.80 min.
Yield 16 mg (33%).
^{1}H-NMR (500 MHz, THF-d$_8$): δ 7.12 (d, J = 7.4 Hz, 1H), 7.04 (dd, J = 1.5, 2.6 Hz, 1H), 6.77 (dd, J = 2.6, 7.7 Hz, 2H), 6.74 (d, J = 2.1 Hz, 1H), 6.72 (dt, J = 1.2, 7.5 Hz, 1H), 6.64 (dd, J = 2.1, 8.1 Hz, 1H), 5.70 (dd, J = 6.2, 7.4 Hz, 1H), 5.66 (d, J = 5.1 Hz, 1H), 4.28 – 4.16 (m, 1H), 4.17 – 4.01 (m, 1H), 3.78 (d, J = 15.2 Hz, 4H), 3.75 (s, 3H), 3.73 (s, 3H), 2.72 – 2.59 (m, 1H), 2.57 – 2.50 (m, 1H), 2.53 – 2.44 (m, 2H), 2.39 – 2.28 (m, 1H), 2.21 – 2.13 (m, 1H), 2.09 – 1.98 (m, 1H), 1.79 (ddd, J = 7.0, 10.3, 14.4 Hz, 2H), 1.70 (d, J = 13.8 Hz, 3H), 1.68 – 1.56 (m, 6H), 1.56 – 1.44 (m, 2H), 1.46 – 1.34 (m, 2H), 1.35 – 1.24 (m, 1H), 1.26 – 1.18 (m, 1H), 1.21 – 1.07 (m, 1H), 1.09 – 0.88 (m, 2H).
^{13}C-NMR (126 MHz, THF-d$_8$): δ 173.73, 171.66, 160.77, 150.95, 149.30, 142.46, 134.83, 129.71, 121.14, 120.22, 118.47, 113.76, 113.45, 112.38, 77.96, 68.69, 56.46, 56.29, 52.27, 46.09, 44.27, 42.34, 38.56, 33.11, 32.01, 30.87, 30.72, 30.32, 27.79, 27.69, 27.61, 27.32, 26.98, 25.98, 21.56.
LC-MS (70-100% B, 19 min): t_R (**9a**) = 7.60 min, m/z : calculated = 578.34 $[M+H]^+$, found = 578.42 $[M+H]^+$.
HRMS (ESI): calculated = 578.34761 $[M+H]^+$, found = 578.34766 $[M+H]^+$, err [ppm] = 0.08.
RP-HPLC (50 – 100% B, 1.5 mL/min, 20 min, 254 nm): t_R (**9a**) = 15.89 min (95% Purity)

(2R,5S,12R)-12-cyclohexyl-2-[2-(3,4-dimethoxyphenyl)ethyl]-3,17-dioxa-10-azatricyclo[16.3.1.0^{5,10}]docosa-1(22),18,20-triene-4,11,14-trione

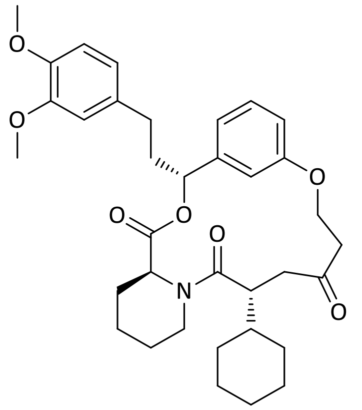

Chemical Formula: $C_{35}H_{45}NO_7$
Exact Mass: 591,32
Molecular Weight: 591,73

9b

8aE (34 mg, 0.059 mmol, 1.0 eq) is dissolved in 0.7 mL THF and 0.1 mL H_2O (7/1, ratio) added. *p*-benzoquinone (7 mg, 0.06 mmol, 1.1 eq), then $PdCl_2$ (2 mg, 0.01 mmol, 0.2 eq) are added. The reaction is stirred over night at r.t. The solvent is removed under reduced pressure and the crude product is purified by silica column chromatography (CH/EE, 3/1) and pure **9b** is obtained.

Yield 27 mg (77%).

^{1}H-NMR (500 MHz, $CDCl_3$): δ 7.19 (t, J = 7.8 Hz, 1H), 6.87 (t, J = 2.1 Hz, 1H), 6.81 – 6.75 (m, 3H), 6.71 – 6.65 (m, 2H), 5.65 (dd, J = 5.3, 7.9 Hz, 1H), 5.39 – 5.33 (m, 1H), 4.46 – 4.33 (m, 2H), 3.91 – 3.87 (m, 1H), 3.86 (s, 3H), 3.85 (s, 3H), 3.36 (td, J = 2.8, 13.3 Hz, 1H), 3.15 – 3.08 (m, 1H), 3.08 – 3.02 (m, 2H), 2.92 (ddd, J = 5.8, 8.2, 16.7 Hz, 1H), 2.67 – 2.58 (m, 2H), 2.54 (dt, J = 4.9, 16.7 Hz, 1H), 2.49 – 2.43 (m, 1H), 2.33 – 2.21 (m, 1H), 2.15 – 2.05 (m, 2H), 1.80 – 1.60 (m, 5H), 1.59 – 1.47 (m, 1H), 1.33 – 1.16 (m, 2H), 1.16 – 1.06 (m, 1H), 1.06 – 0.85 (m, 2H).

^{13}C-NMR (126 MHz, $CDCl_3$): δ 207.80, 174.87, 171.62, 159.77, 149.06, 147.46, 141.64, 133.91, 129.33, 120.31, 119.60, 117.04, 114.99, 111.89, 111.53, 76.04, 65.66, 56.09, 56.00, 51.59, 43.64, 43.58, 42.51, 42.38, 40.36, 37.20, 31.59, 31.37, 30.00, 27.06, 26.67, 26.57, 26.39, 25.57, 19.84.

LC-MS (50-100% B, 19 min): t_R (**9b**) = 10.43 min, m/z : calculated = 592.32 $[M+H]^+$, found = 592.34 $[M+H]^+$.

HRMS (ESI): calculated = 592.32688 $[M+H]^+$, found = 592.32709 $[M+H]^+$, err [ppm] = 0.35.

RP-HPLC (50 – 100% B, 1.5 mL/min, 20 min, 254 nm): t_R (**9b**) = 12.76 min (99% Purity)

(2R,5S,12R)-12-cyclohexyl-2-[2-(3,4-dimethoxyphenyl)ethyl]-14,15-dihydroxy-3,17-dioxa-10-azatricyclo[16.3.1.0⁵,¹⁰]docosa-1(22),18,20-triene-4,11-dione

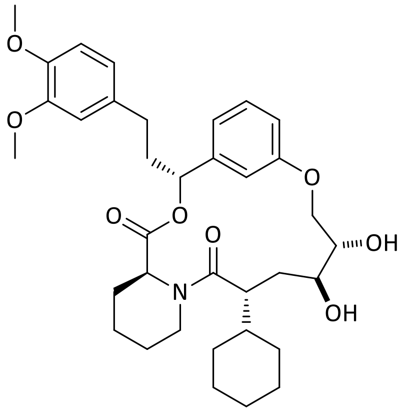

Chemical Formula: $C_{35}H_{47}NO_8$
Exact Mass: 609,33
Molecular Weight: 609,75

9c and **d**

8aE (53 mg, 0.092 mmol, 1.0 eq) is dissolved in 2 mL acetone and 220 µL H_2O (9/1, ratio) added. NMO (16 mg, 0.14 mmol, 1.5 eq), then OsO_4 (23 µL, 0.002 mmol, 0.02 eq) of a 2.5 w% solution in tBuOH are added. The reaction is stirred overnight at r.t. The reaction is quenched with 1 mL sat. $Na_2S_2O_{3(aq)}$ solution and stirred for 15 min. The mixture is diluted with H_2O and extracted with DCM (3x15 mL). The organic phase is washed with 15 mL sat. $CuSO_{4(aq)}$ solution, dried over $MgSO_4$, filtered and the solvent removed under reduced pressure. The crude product is purified by semi-preparative HPLC and pure diastereomers **9c** and **d** obtained.

prep-HPLC (60 – 80% B, 10 mL/min, 20 min, 254 nm): t_R (**9c, d**) = 6.80-7.00 min.
Yield (9c) 15 mg (26%), (**9d**) 13 mg (23%).
TLC (CH/EE, 1/1, v/v): R_f(**9c, d**) = 0.35.
^{1}H-NMR (500 MHz, CDCl$_3$, 9c): δ 7.24 – 7.22 (m, 1H), 7.17 (dd, J = 7.4, 8.3 Hz, 1H), 6.89 – 6.83 (m, 1H), 6.77 (d, J = 7.9 Hz, 1H), 6.75 – 6.71 (m, 1H), 6.69 – 6.64 (m, 2H), 5.67 (dd, J = 5.6, 8.3 Hz, 1H), 5.63 (d, J = 4.6 Hz, 1H), 4.44 (dd, J = 6.1, 12.8 Hz, 1H), 4.10 (dd, J = 5.7, 12.8 Hz, 1H), 3.85 (s, 3H), 3.83 (s, 3H), 3.76 – 3.72 (m, 1H), 3.72 – 3.63 (m, 2H), 3.27 (s, 3H), 2.66 – 2.56 (m, 2H), 2.53 – 2.44 (m, 2H), 2.44 – 2.35 (m, 1H), 2.35 – 2.23 (m, 1H), 2.14 – 2.01 (m, 2H), 1.90 – 1.81 (m, 1H), 1.79 – 1.52 (m, 7H), 1.54 – 1.31 (m, 2H), 1.28 – 0.89 (m, 4H).
^{13}C-NMR (126 MHz, CDCl$_3$, 9c): 174.59, 170.21, 158.97, 148.99, 147.44, 140.67, 133.46, 129.39, 120.27, 120.20, 117.98, 111.87, 111.50, 109.88, 78.00, 72.13, 69.95, 69.83, 56.07, 55.98, 52.17, 44.46, 44.14, 41.66, 36.98, 32.98, 31.80, 31.08, 29.35, 26.82, 26.65, 26.33, 25.67, 20.38, 1.96.
^{1}H-NMR (500 MHz, CDCl$_3$, 9d): δ 7.24 (t, J = 7.8 Hz, 1H), 7.09 – 7.07 (m, 1H), 6.93 – 6.89 (m, 1H), 6.88 – 6.85 (m, 1H), 6.79 (d, J = 7.9 Hz, 1H), 6.70 – 6.65 (m, 2H), 5.65 (t, J = 7.1 Hz, 1H), 5.54 – 5.49 (m, 1H), 4.36 (dd, J = 2.8, 12.6 Hz, 1H), 4.24 (dd, J = 6.1, 12.6 Hz, 1H), 3.90 – 3.88 (m, 1H), 3.86 (s, 3H), 3.85 (s, 3H), 3.68 – 3.63 (m, 1H), 3.61 – 3.56 (m, 1H), 2.96 (td, J = 2.7, 13.4 Hz, 1H), 2.76 – 2.68 (m, 1H), 2.61 – 2.52 (m, 2H), 2.46 – 2.35 (m, 1H), 2.20 – 2.14 (m, 1H), 2.14 – 2.06 (m, 1H), 2.03 – 1.93 (m, 1H), 1.83 (d, J = 12.9 Hz, 1H), 1.77 – 1.55 (m, 4H), 1.55 – 1.36 (m, 1H), 1.29 – 1.04 (m, 3H), 1.04 – 0.83 (m, 2H).
^{13}C-NMR (126 MHz, CDCl$_3$, 9d): δ 176.39, 171.95, 159.08, 149.07, 147.55, 140.97, 133.35, 130.22, 120.56, 120.36, 118.73, 112.53, 111.87, 111.56, 77.79, 72.74, 70.52, 68.89, 56.10, 56.00, 52.39, 44.35, 43.38, 41.28, 36.14, 34.10, 32.12, 31.37, 30.36, 26.57, 26.53, 26.44, 25.55, 20.13.
LC-MS (50-100% B, 19 min): t_R (**9c**) = 6.38 min, m/z : calculated = 610.33 $[M+H]^+$, found = 610.40 $[M+H]^+$, t_R (**9d**) = 7.12 min, m/z : calculated = 610.33 $[M+H]^+$, found = 610.44 $[M+H]^+$.
HRMS (ESI, 9c): calculated = 610.33744 $[M+H]^+$, found = 610.33760 $[M+H]^+$, err [ppm] = 0.26.
HRMS (ESI, 9d): calculated = 610.33744 $[M+H]^+$, found = 610.33762 $[M+H]^+$, err [ppm] = 0.28.
RP-HPLC (50 – 100% B, 1.5 mL/min, 15 min, 254 nm): t_R (**9c**) = 6.49 min (94% Purity).

RP-HPLC (50 – 100% B, 1.5 mL/min, 15 min, 254 nm): t_R (**9d**) = 7.46 min (95% Purity).

(2R,5S,12R)-12-cyclohexyl-2-[2-(3,4-dimethoxyphenyl)ethyl]-3,17,20-trioxa-10-azatricyclo[19.3.1.0^{5,10}]pentacosa-1(25),21,23-triene-4,11-dione

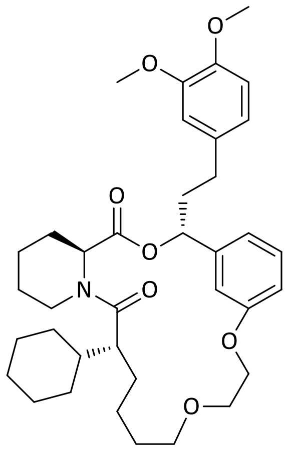

Chemical Formula: $C_{37}H_{51}NO_7$
Exact Mass: 621,37
Molecular Weight: 621,80

9e

8b (30 mg, 0.048 mmol, 1.0 eq), $RuCl(PPh_3)_3$ (22 mg, 0.024 mmol, 0.5 eq) is dissolved in 3 mL toluene. The solution is sparged with H_2 for 10 min, then reacted under 1 bar H_2 atmosphere over night at r.t. The solvent is removed and the crude product purified by silica column chromatography (CH/EE, 3/1) to obtain pure product **9e**.

Yield 28 mg (93%).
TLC (CH/EE, 3/1, v/v): R_f (**9e**) = 0.25.
^{1}H-NMR (500 MHz, CD_2Cl_2): δ 7.23 (t, J = 7.9 Hz, 1H), 7.20 (t, J = 2.1 Hz, 1H), 6.92 – 6.89 (m, 1H), 6.89 – 6.86 (m, 1H), 6.77 (d, J = 8.6 Hz, 1H), 6.71 – 6.65 (m, 2H), 5.81 (dd, J = 5.6, 8.3 Hz, 1H), 5.66 – 5.61 (m, 1H), 4.26 – 4.11 (m, 2H), 3.97 – 3.91 (m, 1H), 3.86 (s, 3H), 3.85 (s, 3H), 3.67 (ddd, J = 2.7, 4.9, 11.5 Hz, 1H), 3.59 – 3.48 (m, 2H), 3.39 (ddd, J = 4.8, 7.2, 9.3 Hz, 1H), 3.02 – 2.94 (m, 1H), 2.68 – 2.57 (m, 1H), 2.57 – 2.47 (m, 2H), 2.32 – 2.26 (m, 1H), 2.26 – 2.18 (m, 1H), 2.09 – 1.99 (m, 1H), 1.91 – 1.83 (m, 1H), 1.82 – 1.48 (m, 11H), 1.46 – 1.33 (m, 5H), 1.30 – 1.17 (m, 2H), 1.17 – 1.06 (m, 1H), 1.01 – 0.84 (m, 2H).
^{13}C-NMR (126 MHz, CD_2Cl_2): δ 175.49, 171.39, 159.24, 149.04, 147.44, 141.78, 133.93, 129.52, 121.03, 120.38, 117.24, 116.19, 112.00, 111.52, 76.08, 71.45, 70.44, 69.15, 56.09, 55.99, 51.99, 46.95, 44.07, 41.19, 38.22, 30.68, 30.60, 30.55, 27.07, 26.94, 26.64, 26.59, 25.76, 25.27, 21.02.
LC-MS (70-100% B, 19 min): t_R (**9e**) = 8.41 min, m/z : calculated = 622.37 $[M+H]^+$, found = 622.50 $[M+H]^+$.
HRMS (ESI): calculated = 622.37383 $[M+H]^+$, found = 622.37352 $[M+H]^+$, err [ppm] = 0.50.
RP-HPLC (30 – 100% B, 1.5 mL/min, 20 min, 220 nm): t_R (**9e**) = 18.54 min (96% Purity).

(2R,5S,12R)-12-cyclohexyl-2-[2-(3,4-dimethoxyphenyl)ethyl]-3,17,20-trioxa-10-azatricyclo[19.3.1.0^{5,10}]pentacosa-1(25),21,23-triene-4,11,14-trione

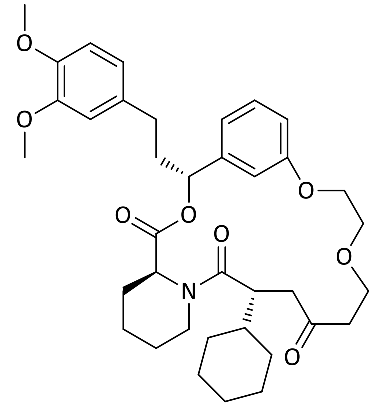

Chemical Formula: $C_{37}H_{49}NO_8$
Exact Mass: 635,35
Molecular Weight: 635,79

9f

8b (50 mg, 0.081 mmol, 1.0 eq) is dissolved in 0.7 mL THF and 0.1 mL H_2O (7/1 ratio) added. *p*-benzoquinone (10 mg, 0.09 mmol, 1.1 eq), then $PdCl_2$ (4 mg, 0.016 mmol, 0.4 eq) are added. The reaction is stirred overnight at r.t. The solvent is removed under reduced pressure and the crude product is purified by semi-preparative HPLC and pure **9f** obtained.

prep-HPLC (60 – 100% B, 10 mL/min, 10 min, 254 nm): t_R (**9f**) = 7.93 min.
Yield 23 mg (45%).
^{1}H-NMR (500 MHz, $CDCl_3$): δ 7.20 (t, J = 7.9 Hz, 1H), 7.05 (t, J = 2.0 Hz, 1H), 6.83 (d, J = 1.2, 7.8 Hz, 1H), 6.82 – 6.76 (m, 2H), 6.71 – 6.66 (m, 2H), 5.73 (dd, J = 5.4, 8.1 Hz, 1H), 5.45 (d, 1H), 4.16 – 4.09 (m, 2H), 3.93 (d, J = 13.3 Hz, 1H), 3.90 – 3.81 (m, 6H), 3.76 – 3.70 (m, 2H), 3.69 – 3.61 (m, 1H), 3.25 – 3.18 (m, 1H), 3.16 – 3.08 (m, 1H), 2.83 (dd, J = 6.0, 17.9 Hz, 1H), 2.76 (ddd, J = 4.9, 8.3, 16.4 Hz, 1H), 2.66 (dd, J = 6.1, 18.1 Hz, 1H), 2.63 – 2.50 (m, 2H), 2.43 (dt, J = 5.0, 16.4 Hz, 1H), 2.31 – 2.16 (m, 2H), 2.13 – 2.01 (m, 1H), 1.91 – 1.82 (m, 1H), 1.77 – 1.57 (m, 7H), 1.57 – 1.50 (m, 1H), 1.49 – 1.35 (m, 2H), 1.31 – 1.18 (m, 2H), 1.19 – 1.05 (m, 1H), 1.03 – 0.79 (m, 2H).
^{13}C-NMR (126 MHz, $CDCl_3$): δ 207.55, 174.71, 171.04, 159.35, 149.05, 147.48, 141.88, 133.88, 129.41, 120.37, 119.60, 114.89, 114.43, 111.98, 111.53, 76.07, 69.79, 68.59, 66.15, 56.09, 56.02, 52.09, 44.00, 43.86, 43.04, 41.14, 40.57, 37.99, 31.74, 31.28, 30.25, 26.91, 26.62, 26.51, 26.43, 25.64, 20.87.
LC-MS (50-100% B, 19 min): t_R (**9f**) = 9.88 min, m/z : calculated = 636.35 $[M+H]^+$, found = 636.44 $[M+H]^+$.
HRMS (ESI): calculated = 636.35309 $[M+H]^+$, found = 636.35343 $[M+H]^+$, err [ppm] = 0.52.
RP-HPLC (50 – 100% B, 1.5 mL/min, 15 min, 254 nm): t_R (**9f**) = 10.93 min (99% Purity)

(2R,5S,12R)-12-cyclohexyl-2-[2-(3,4-dimethoxyphenyl)ethyl]-14,15-dihydroxy-3,17,20-trioxa-10-azatricyclo[19.3.1.0⁵,¹⁰]pentacosa-1(25),21,23-triene-4,11-dione

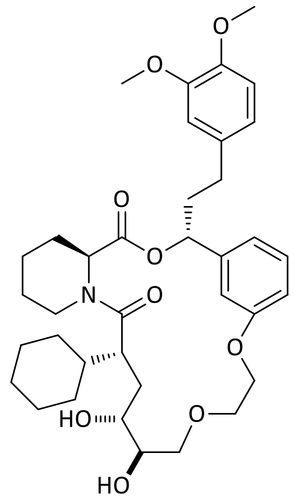

Chemical Formula: $C_{37}H_{51}NO_9$
Exact Mass: 653,36
Molecular Weight: 653,80

9g

8b (53 mg, 0.086 mmol, 1.0 eq) is dissolved in 2 mL acetone and 220 µL H_2O (9/1, ratio) added. NMO (12 mg, 0.1 mmol, 1.2 eq), then OsO_4 (21 µL, 0.002 mmol, 0.02 eq) of a 2.5 w% solution in tBuOH are added. The reaction is stirred overnight at r.t. The reaction is quenched with 1 mL sat. $Na_2S_2O_{3(aq)}$ solution and stirred for 15 min. The mixture is diluted with H_2O and extracted with DCM (3x15 mL). The organic phase is washed with 15 mL sat. $CuSO_{4(aq)}$ solution, dried over $MgSO_4$, filtered and the solvent removed under reduced pressure. The crude product is purified by semi-preparative HPLC and pure **9g** obtained. (Diastereomers could not be separated on either prep. HPLC or RP-HPLC)

prep-HPLC (55 – 65% B, 10 mL/min, 10 min, 254 nm): t_R (**9g**) = 9.25 min.
Yield 19 mg (34%).
^{1}H-NMR (500 MHz, CDCl$_3$): δ 7.29 – 7.26 (m, 1H), 7.25 – 7.21 (m, 1H), 7.01 (t, J = 2.0 Hz, 1H), 6.93 – 6.85 (m, 2H), 6.81 – 6.74 (m, 1H), 6.71 – 6.65 (m, 2H), 5.76 (dd, J = 5.9, 8.1 Hz, 0.57H), 5.70 (dd, J = 5.2, 8.3 Hz, 0.43H), 5.58 (d, J = 1.9, 6.2 Hz, 0.50H), 5.55 (d, 0.64H), 4.35 – 4.15 (m, 2H), 4.05 (d, J = 13.6 Hz, 0.42H), 3.91 – 3.81 (m, 6H), 3.79 – 3.42 (m, 6H), 3.40 – 3.29 (m, 0.53H), 3.24 – 3.09 (m, 0.51H), 3.03 – 2.85 (m, 1H), 2.82 – 2.46 (m, 2H), 2.40 – 2.20 (m, 2H), 2.17 – 1.95 (m, 1H), 1.95 – 1.80 (m, 1H), 1.80 – 1.34 (m, 8H), 1.30 – 0.87 (m, 4H).
^{13}C-NMR (126 MHz, CDCl$_3$): δ 176.10, 175.88, 172.13, 171.46, 159.37, 158.55, 149.14, 149.06, 147.60, 147.49, 141.68, 141.42, 133.75, 133.66, 129.85, 129.75, 129.55, 120.54, 120.33, 119.94, 117.36, 116.91, 116.16, 116.01, 111.95, 111.58, 111.52, 76.46, 74.79, 73.53, 73.16, 72.15, 70.67, 70.64, 70.46, 69.86, 69.63, 69.34, 56.10, 56.04, 56.00, 52.19, 52.16, 44.25, 44.07, 44.03, 42.82, 41.62, 41.22, 37.94, 37.37, 34.09, 33.41, 31.93, 31.67, 31.53, 31.49, 30.45, 29.80, 27.11, 26.83, 26.75, 26.70, 26.68, 26.56, 26.54, 26.46, 25.70, 25.62, 21.04, 20.89.
LC-MS (50-100% B, 19 min): t_R (**9g**) = 7.68 min, m/z : calculated = 654.36 $[M+H]^+$, found = 654.51 $[M+H]^+$.
HRMS (ESI): calculated = 654.36366 $[M+H]^+$, found = 654.36352 $[M+H]^+$, err [ppm] = 0.21.
RP-HPLC (50 – 100% B, 1.5 mL/min, 15 min, 254 nm): t_R (**9g**) = 7.95 min (99% Purity, 1/1 dr determined by NMR).

9.3. Solid phase peptide synthesis (SPPS, 2nd macrocycle generation)

9.3.1 General procedures

Test cleavage

After washing the resin with DCM (3×) a few resin beads are taken and transferred into an eppendorf tube. 0.5 mL of a 20% HFIP in DCM solution is added and the product cleaved off the resin for 15 min. Then the solvent is evaporated under air stream and the solid leftover is suspended in acetonitrile + 0.1% HCOOH (HPLC grade), filtered and a LC-MS spectrum taken.

Chloranil test

After washing the resin with DCM (3×) a few resin beads are taken from the filter syringe and transferred into an eppendorf tube. Then 1-2 drops of a 2% acetaldehyde in DMF solution and 1-2 drops of a 2% chloranil in DMF solution are added to the resin. Deprotected secondary amines are confirmed by a quickly blue coloring of the beads, primary amines can also be confirmed but the reaction takes several minutes (5-10 min).

Kaiser test

After washing the resin with DCM (3×) a few resin beads are taken from the filter syringe and transferred into an eppendorf tube. Then 1-2 drops of a 0.5 g ninhydrine in 10 mL EtOH solution, 1-2 drops of a 2 g Phenol in 0.5 mL EtOH solution and 1-2 drops of a mixture of 0.2 mL 0.001 M KCN in H_2O mixed with 9.8 mL pyridine are added to the resin. The tube is carefully heated to 90°C by heating block for 1-2 minutes. Deprotected primary amines are confirmed by a purple coloring of the beads.

SPPS and macrocyclization[10]

a) Resin loading: 2-Chlorotrityl chloride resin (2.0 eq) is placed into a dried and heated flask and swelled for 30 min in dry DCM (30 mL/g resin) at r.t. under argon protection. The compound being loaded (1.0 eq) is dissolved in a minimum amount of dry DCM, mixed with DIPEA (4 eq) and the resulting mixture is then added to the resin and stirred at r.t. overnight. After complete loading (TLC check of the solution) the resin is capped for 30 min by addition of 100 µL/g resin dry MeOH and 1 eq DIPEA. The resin is then filtered, washed with DMF (3×), DCM (3×) and dried in the desiccator overnight. The loading *l* is determined as follows:[10]

$$\frac{(m_{total} - m_{resin}) \cdot 10^3}{(MW - 36.46) \cdot m_{total}} = l \ {}^{mmol}/_{g}$$

b) 1st deprotection: The needed amount of resin calculated by the resin loading is transferred to a syringe with a fritted-glas filter. The resin is swelled for 20 min in DCM, then washed with DMF 3x. The pipecolate is Fmoc-deprotected by shaking in 20% 4-methyl piperidine in DMF (3x10 min). The completion of the reaction is monitored on LCMS by test cleavage as well as by Chloranil-test.

c) 1st AA coupling: Fmoc-AA-OH, (3.0 eq respectively to the resin loading), HATU (3.0 eq) and HOAt (3.0 eq) are dissolved in a minimum amount of DMF by sonication. Then DIPEA (6.0 eq) is added and the mixture is drawn up into the filter syringe filled with loaded resin. The syringe is mixed by shaking for 2 h or with especially hindered substrates overnight. Finally, the solvent is removed and the resin is washed with DMF (3×), THF (3×), DCM (3×). The coupling is confirmed via test cleavage and/or by Chloranil-test.

d) 2nd deprotection: The Fmoc protecting group is removed by the addition of 5% 4-methyl piperidine in DMF solution pre-cooled in an ice bath to 4 °C to the filter syringes. After 5 min the deprotection solution is removed and the resin is washed with DMF (1×). The completion of the reaction is confirmed via test cleavage and/or by Kaiser-test. The deprotection procedure is repeated two times if needed. After the final step the resin is washed with DMF (3×), then DCM (3×).

e) 2nd AA coupling: Repeat entry **c)**. in case of an *N*-methylation Fmoc-sarcosin is coupled or steps **g)** to **i)** applied.

f) 3rd deprotection: Repeat entry **d)**; in case of the meta OH B ring derivative **23** an additional deprotection for the allyl is done by the addition of $Pd(OAc)_2$ (0.1 eq), PPh_3 (1.0 eq) and morpholine (2.0 eq) in THF. After addition to the syringe the resin is mixed for 20 min. The completion of the reaction is confirmed via test cleavage.

g) Nosyl protection (for optional *N*-methylation): *o*-Nitrobenzene sulfonychloride (4 eq) is dissolved in NMP and 2, 4, 6-collidine (10.0 eq) is added. The mixture is drawn up into the syringe with resin and reacted for 15 min. This procedure is repeated 2x. After the final step the resin is washed with DMF (3×), then DCM (3×). The completion of the reaction is confirmed via test cleavage and/or by Kaiser-test.

h) *N*-methylation: The resin is washed with dry THF (3x) and a solution of PPh_3 (5.0 eq) and dry MeOH (10.0 eq) in dry THF are added. Then DIAD (5.0 eq) diluted in dry THF is added portion wise (Caution! Exothermic reaction!). After 10 min the reaction mixture is discarded, the resin washed with dry THF and the procedure repeated 2x. After the final step the resin is washed with DMF (3×), then DCM (3×). The completion of the reaction is confirmed via test cleavage.

i) Nosyl deprotection (after optional *N*-methylation): DBU (5.0 eq) and beta-mercaptoethanol (10.0 eq) are dissolved in NMP. The mixture is drawn up into the syringe with resin and reacted for 10 min. This procedure is repeated if needed. After the final step the resin is washed with DMF (3×), then DCM (3×). The completion of the reaction is confirmed via test cleavage and/or by Chloranil-test.

j) Cleavage from resin: The resin is transferred to a round bottom flask and stirred in 20 mL/g resin 20% HFIP in DCM for 2 h. The resin is filtered off and washed with DCM. The solvent is removed and the crude linear product identified by LCMS.

k) Macrocyclization: The crude linear product is dissolved in DMF (1.0 mM calculated as if pure compound) and HATU (3 eq) and DIPEA (5.0 eq) added. In case of the meta OH B ring derivative **25** another method is used: HATU (1.0 eq), DIPEA (3.0 eq) pentafluoro phenol (10.0 eq) in NMP (1 mM). The reaction is stirred at r.t. overnight and the solvent removed under reduced pressure. The crude product is purified by silica column chromatography and/or semi-preparative HPLC.

9.3.2 Macrocycle synthesis

(2R,5S,12R)-12-cyclohexyl-2-[2-(3,4-dimethoxyphenyl)ethyl]-3,19-dioxa-10,13,16-triazatricyclo[18.3.1.0^{5,10}]tetracosa-1(24),20,22-triene-4,11,14,17-tetrone

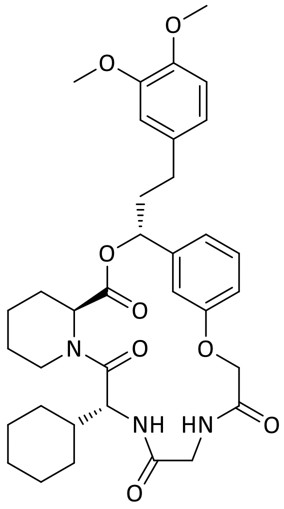

Chemical Formula: $C_{35}H_{45}N_3O_8$
Exact Mass: 635,32
Molecular Weight: 635,75

13a

Starting materials: Resin: (0.17 mmol), 1st AA: Fmoc-D-Chg-OH, 2nd AA: Fmoc-Gly-OH, Procedure as described at p. 32.

prep-HPLC (80 – 100% B, 10 mL/min, 10 min, 254 nm): t_R (**13a**) = 9.50 min.

Yield 11 mg (10%).

^{1}H-NMR (500 MHz, CDCl$_3$): δ 7.29 – 7.21 (m, 1H), 7.14 (d, J = 9.1 Hz, 1H), 6.96 – 6.87 (m, 3H), 6.88 – 6.82 (m, 1H), 6.77 (d, J = 7.6 Hz, 1H), 6.69 – 6.61 (m, 2H), 5.67 – 5.59 (m, 1H), 5.26 – 5.18 (m, 1H), 4.82 – 4.74 (m, 1H), 4.69 – 4.57 (m, 2H), 4.57 – 4.43 (m, 1H), 3.86 (s, 3H), 3.84 (s, 3H), 3.80 – 3.73 (m, 1H), 3.37 (dd, J = 4.3, 14.8 Hz, 1H), 3.16 (td, J = 2.9, 13.2 Hz, 1H), 2.66 – 2.39 (m, 1H), 2.37 – 2.22 (m, 1H), 2.21 – 2.11 (m, 1H), 2.10 – 1.98 (m, 1H), 1.94 – 1.79 (m, 2H), 1.80 – 1.67 (m, 2H), 1.69 – 1.53 (m, 8H), 1.54 – 1.34 (m, 1H), 1.30 – 1.10 (m, 2H), 1.09 – 0.93 (m, 2H).

^{13}C-NMR (126 MHz, CDCl$_3$): δ 172.01, 171.50, 170.31, 169.37, 157.84, 149.02, 147.48, 141.76, 133.42, 129.96, 121.32, 120.27, 116.67, 111.78, 111.49, 111.44, 109.57, 76.65, 66.89, 56.06, 55.98, 53.59, 52.56, 44.00, 43.88, 41.12, 37.32, 31.35, 30.12, 28.14, 26.41, 26.11, 26.03, 25.22, 19.99.

LC-MS (80-100% B, 19 min): t_R (**13a**) = 2.30 min, m/z : calculated = 636.33 [M+H]$^+$, found = 636.07 [M+H]$^+$.

HRMS (ESI): calculated = 636.32794 [M+H]$^+$, found = 636.32770 [M+H]$^+$, err [ppm] = 0.37.

RP-HPLC (0 – 100% B, 1.5 mL/min, 30 min, 220 nm): t_R (**13a**) = 19.97 min (95% Purity)

(2R,5S,12R,15R)-12-cyclohexyl-2-[2-(3,4-dimethoxyphenyl)ethyl]-15-methyl-3,19-dioxa-10,13,16-triazatricyclo[18.3.1.0^{5,10}]tetracosa-1(24),20,22-triene-4,11,14,17-tetrone

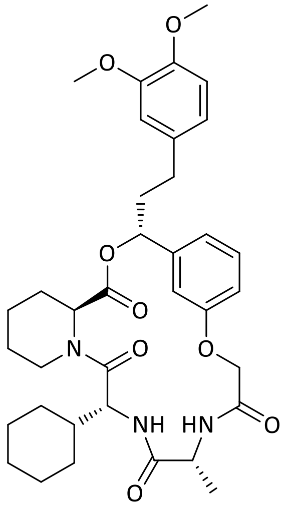

Chemical Formula: $C_{36}H_{47}N_3O_8$
Exact Mass: 649,34
Molecular Weight: 649,77

13b

Starting materials: Resin: (0.14 mmol), 1st AA: Fmoc-D-Chg-OH, 2nd AA: Fmoc-D-Ala-OH, Procedure as described at p. 32.

Silica column chromatography: DCM/MeOH, 8/2, v/v.

prep-HPLC (80 – 100% B, 10 mL/min, 10 min, 254 nm): t_R (**13b**) = 11.80 min.

Yield 17 mg (19%).

^{1}H-NMR (800 MHz, DMSO-d_6 + CD_2Cl_2): δ 8.31 (d, J = 7.2 Hz, 1H), 7.75 (d, J = 9.0 Hz, 1H), 7.20 (t, J = 7.8 Hz, 1H), 6.90 – 6.86 (m, 1H), 6.87 – 6.82 (m, 3H), 6.78 (d, J = 2.0 Hz, 1H), 6.67 (dd, J = 2.0, 8.1 Hz, 1H), 5.62 (dd, J = 5.0, 8.6 Hz, 1H), 5.08 – 5.04 (m, 1H), 4.73 (d, J = 15.8 Hz, 1H), 4.61 (d, J = 15.8 Hz, 1H), 4.48 (dd, J = 7.0, 9.1 Hz, 1H), 4.08 (p, J = 7.3 Hz, 1H), 3.73 (s, 3H), 3.71 (s, 3H), 3.70 – 3.65 (m, 1H), 3.00 – 2.93 (m, 1H), 2.59 – 2.52 (m, 1H), 2.49 – 2.43 (m, 1H), 2.17 – 2.10 (m, 1H), 2.06 – 1.99 (m, 2H), 1.79 – 1.72 (m, 1H), 1.72 – 1.68 (m, 1H), 1.67 – 1.58 (m, 6H), 1.58 – 1.54 (m, 1H), 1.40 – 1.33 (m, 2H), 1.31 (d, J = 7.3 Hz, 3H), 1.22 – 1.11 (m, 2H), 1.09 – 1.03 (m, 1H), 0.98 – 0.89 (m, 2H).

^{13}C-NMR (201 MHz, DMSO-d_6 + CD_2Cl_2): δ 172.16, 171.15, 170.56, 167.97, 158.76, 148.66, 147.06, 141.43, 133.34, 128.95, 120.00, 119.50, 116.36, 112.26, 111.89, 110.08, 75.41, 65.71, 55.51, 55.37, 52.48, 52.10, 50.28, 42.48, 40.25, 37.37, 30.71, 30.11, 27.16, 26.02, 25.87, 25.67, 25.61, 24.46, 19.53, 18.04.

LC-MS (0-100% B, 19 min): t_R (**13b**) = 12.00 min, m/z : calculated = 650.34 $[M+H]^+$, found = 650.09 $[M+H]^+$.

HRMS (ESI): calculated = 650.34359 $[M+H]^+$, found = 650.34361 $[M+H]^+$, err [ppm] = 0.03.

RP-HPLC (0 – 100% B, 1.5 mL/min, 20 min, 220 nm): t_R (**13b**) = 20.35 min (97% Purity)

(2R,5S,12R)-12-cyclohexyl-2-[2-(3,4-dimethoxyphenyl)ethyl]-15,15-dimethyl-3,19-dioxa-10,13,16-triazatricyclo[18.3.1.0^{5,10}]tetracosa-1(24),20,22-triene-4,11,14,17-tetrone

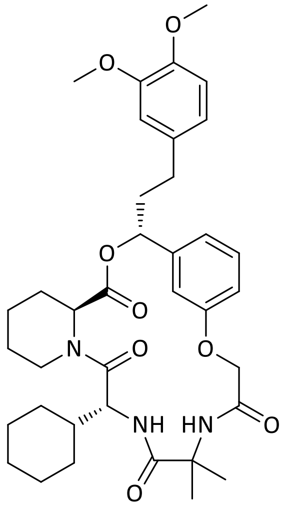

Chemical Formula: $C_{37}H_{49}N_3O_8$
Exact Mass: 663,35
Molecular Weight: 663,80

13c

Starting materials: Resin: (0.47 mmol), 1st AA: Fmoc-D-Chg-OH, 2nd AA: Fmoc-Aib-OH, Procedure as described at p. 32.

Silica column chromatography: DCM/Ac, 10/1, v/v.

TLC (DCM/Ac, 10/1): R_f (**13c**) = 0.35.

Yield 257 mg (83%).

^{1}H-NMR (500 MHz, CDCl$_3$): δ 7.73 (d, J = 9.0 Hz, 1H), 7.25 – 7.19 (m, 1H), 6.99 – 6.96 (m, 1H), 6.85 – 6.80 (m, 2H), 6.77 (d, J = 8.0 Hz, 1H), 6.68 – 6.62 (m, 2H), 6.48 (s, 1H), 5.65 (dd, J = 5.7, 7.6 Hz, 1H), 5.25 – 5.20 (m, 1H), 4.69 (dd, J = 6.1, 9.1 Hz, 1H), 4.64 (d, J = 16.1 Hz, 1H), 4.54 (d, J = 16.1 Hz, 1H), 3.86 (s, 3H), 3.84 (s, 3H), 3.81 – 3.75 (m, 1H), 3.28 (td, J = 3.1, 13.1 Hz, 1H), 2.62 – 2.44 (m, 2H), 2.31 – 2.20 (m, 1H), 2.16 – 2.10 (m, 1H), 2.08 – 2.00 (m, 1H), 1.99 (s, 1H), 1.82 (s, 1H), 1.78 (s, 3H), 1.77 – 1.60 (m, 10H), 1.41 (s, 3H), 1.30 – 1.17 (m, 2H), 1.16 – 0.97 (m, 2H).

^{13}C-NMR (126 MHz, CDCl$_3$): δ 174.04, 172.32, 171.69, 169.57, 157.60, 149.07, 147.53, 142.13, 133.55, 129.78, 120.64, 120.27, 115.19, 111.84, 111.54, 110.27, 76.29, 66.95, 58.76, 56.08, 56.00, 53.63, 52.52, 51.94, 43.83, 41.40, 37.83, 31.25, 30.34, 26.45, 26.20, 26.12, 25.47, 25.22, 24.81, 19.89.

LC-MS (50-100% B, 19 min): t_R (**13c**) = 8.25 min, m/z : calculated = 664.35 [M+H]$^+$, found = 664.32 [M+H]$^+$.

HRMS (ESI): calculated = 664.35924 [M+H]$^+$, found = 664.36019 [M+H]$^+$, err [ppm] = 1.42.

RP-HPLC (0 – 100% B, 1.5 mL/min, 20 min, 220 nm): t_R (**13c**) = 19.92 min (96% Purity)

(2'R,5'S,12'R)-12'-cyclohexyl-2'-[2-(3,4-dimethoxyphenyl)ethyl]-3',19'-dioxa-10',13',16'-triazaspiro[cyclopropane-1,15'-tricyclo[18.3.1.0^{5,10}]tetracosane]-1'(24'),20',22'-triene-4',11',14',17'-tetrone

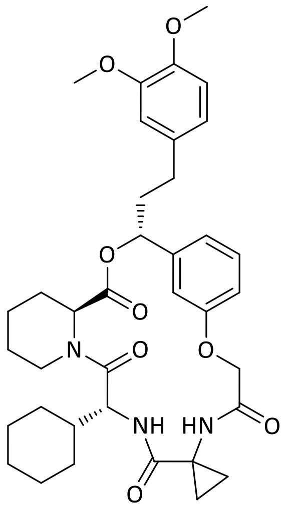

Chemical Formula: $C_{37}H_{47}N_3O_8$
Exact Mass: 661,34
Molecular Weight: 661,78

13d

Starting materials: Resin (0.05 mmol), 1st AA: Fmoc-D-Chg-OH, 2nd AA: Fmoc-1-amino-1-cyclopropanecarboxylic acid, Procedure as described at p. 32.

Silica column chromatography: DCM/MeOH, 95/5, v/v.

prep-HPLC (80 – 100% B, 10 mL/min, 20 min, 254 nm): t_R (**13d**) = 10.40 min.

Yield 27 mg (77%).

^{1}H-NMR (599 MHz, DMSO-d_6 + CD_2Cl_2): δ 8.12 (s, 1H), 7.46 (d, J = 9.0 Hz, 1H), 6.77 (t, J = 7.8 Hz, 1H), 6.51 (d, J = 2.4 Hz, 1H), 6.43 – 6.38 (m, 3H), 6.35 – 6.31 (m, 1H), 6.24 (dd, J = 2.1, 8.0 Hz, 1H), 5.22 (dd, J = 5.3, 8.3 Hz, 1H), 4.61 (dd, J = 2.7, 6.3 Hz, 1H), 4.34 (d, J = 16.0 Hz, 1H), 4.10 (d, J = 15.9 Hz, 1H), 4.04 (t, J = 8.2 Hz, 1H), 3.30 (s, 3H), 3.27 (s, 4H), 3.17 (d, J = 13.7 Hz, 1H), 2.50 (t, J = 12.5 Hz, 1H), 2.05 – 1.96 (m, 1H), 1.75 – 1.67 (m, 1H), 1.66 – 1.54 (m, 3H), 1.40 – 1.28 (m, 1H), 1.30 – 1.13 (m, 6H), 1.13 – 1.03 (m, 2H), 0.98 – 0.86 (m, 2H), 0.85 – 0.66 (m, 2H), 0.66 – 0.55 (m, 1H), 0.55 – 0.41 (m, 3H), 0.39 – 0.29 (m, 1H).

^{13}C-NMR (126 MHz, DMSO-d_6 + CD_2Cl_2): δ 185.63, 185.35, 184.66, 183.74, 173.34, 163.11, 161.51, 155.74, 147.84, 143.26, 134.47, 133.90, 131.29, 126.72, 126.36, 124.21, 90.10, 79.77, 69.99, 69.84, 67.92, 66.88, 56.94, 54.96, 52.06, 48.52, 45.21, 44.85, 41.57, 40.47, 40.38, 40.27, 40.14, 38.96, 34.12, 30.32, 28.75.

LC-MS (0-100% B, 19 min): t_R (**13d**) = 12.24 min, m/z : calculated = 662.34 $[M+H]^+$, found = 662.32 $[M+H]^+$.

HRMS (ESI): calculated = 662.34359 $[M+H]^+$, found = 662.34412 $[M+H]^+$, err [ppm] = 0.79.

RP-HPLC (0 – 100% B, 1.5 mL/min, 20 min, 220 nm): t_R (**13d**) = 20.70 min (99% Purity)

(2'R,5'S,12'R)-12'-cyclohexyl-2'-[2-(3,4-dimethoxyphenyl)ethyl]-3',19'-dioxa-10',13',16'-triazaspiro[cyclobutane-1,15'-tricyclo[18.3.1.0^{5,10}]tetracosane]-1'(24'),20',22'-triene-4',11',14',17'-tetrone

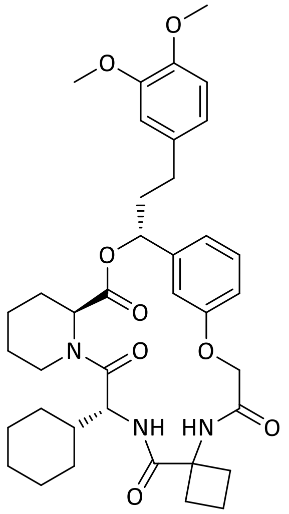

Chemical Formula: $C_{38}H_{49}N_3O_8$
Exact Mass: 675,35
Molecular Weight: 675,81

13e

Starting materials: Resin: (0.19 mmol), 1st AA: Fmoc-D-Chg-OH, 2nd AA: Fmoc-1-amino-1-cyclobutanecarboxylic acid, Procedure as described at p. 32.

Silica column chromatography: DCM/acetone, 10/1, v/v.
TLC (DCM/acetone, 10/1): R_f (**13e**) = 0.55.
prep-HPLC (50 – 100% B, 10 mL/min, 10 min, 254 nm): t_R (**13e**) = 8.00 min.
Yield 38 mg (29%).
^{1}H-NMR (500 MHz, $CDCl_3$): δ 7.87 (d, J = 9.5 Hz, 1H), 7.23 – 7.17 (m, 1H), 6.89 – 6.83 (m, 2H), 6.81 – 6.72 (m, 2H), 6.64 – 6.61 (m, 2H), 6.57 (s, 1H), 5.60 (t, J = 6.6 Hz, 1H), 5.26 (q, J = 2.1 Hz, 1H), 4.75 (d, J = 16.5 Hz, 1H), 4.71 (dd, J = 6.4, 9.5 Hz, 1H), 4.58 (d, J = 16.6 Hz, 1H), 3.84 (s, 3H), 3.82 (s, 3H), 3.81 – 3.77 (m, 1H), 3.22 – 3.14 (m, 1H), 2.94 – 2.85 (m, 1H), 2.78 – 2.69 (m, 1H), 2.59 – 2.52 (m, 1H), 2.52 – 2.40 (m, 1H), 2.29 – 2.17 (m, 1H), 2.14 – 2.06 (m, 1H), 2.03 – 1.81 (m, 4H), 1.80 – 1.59 (m, 11H), 1.51 – 1.38 (m, 1H), 1.31 – 1.19 (m, 2H), 1.19 – 1.09 (m, 1H), 1.08 – 1.00 (m, 1H).
^{13}C-NMR (126 MHz, $CDCl_3$): δ 173.13, 171.95, 171.45, 169.89, 157.76, 149.01, 147.45, 142.16, 133.47, 129.75, 120.72, 120.20, 116.94, 111.76, 111.47, 107.68, 76.51, 66.58, 59.87, 56.03, 55.95, 53.44, 52.32, 43.81, 41.27, 38.36, 32.25, 31.12, 30.36, 29.36, 28.06, 26.49, 26.20, 26.14, 26.08, 25.31, 19.94, 15.71.
LC-MS (50-100% B, 19 min): t_R (**13e**) = 9.47 min, m/z : calculated = 676.36 $[M+H]^+$, found = 676.48 $[M+H]^+$.
HRMS (ESI): calculated = 676.35924 $[M+H]^+$, found = 676.35936 $[M+H]^+$, err [ppm] = 0.17.
RP-HPLC (50 – 100% B, 1.5 mL/min, 20 min, 220 nm): t_R (**13e**) = 14.61 min (98% Purity)

(2'R,5'S,12'R)-12'-cyclohexyl-2'-[2-(3,4-dimethoxyphenyl)ethyl]-3',19'-dioxa-10',13',16'-triazaspiro[cyclopentane-1,15'-tricyclo[18.3.1.0⁵,¹⁰]tetracosane]-1'(24'),20',22'-triene-4',11',14',17'-tetrone

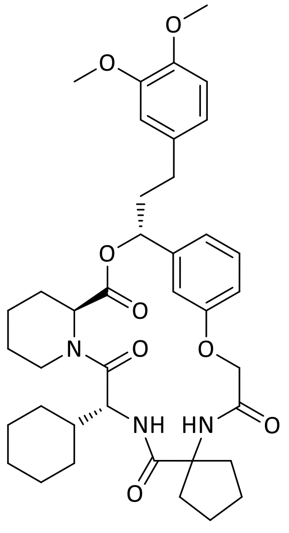

Chemical Formula: $C_{39}H_{51}N_3O_8$
Exact Mass: 689,37
Molecular Weight: 689,84

13f

Starting materials: Resin: (0.14 mmol), 1st AA: Fmoc-D-Chg-OH, 2nd AA: Fmoc-1-amino-1-cyclopentanecarboxylic acid, Procedure as described at p. 32.

prep-HPLC (90 – 100% B, 10 mL/min, 10 min, 254 nm): t_R (**13f**) = 5.01 min.

Silica column chromatography: DCM/MeOH, 95/5, v/v.

Yield 4 mg (4%).

LC-MS (50-100% B, 19 min): t_R (**13f**) = 13.04 min, m/z : calculated = 690.38 $[M+H]^+$, found = 690.36 $[M+H]^+$.

HRMS (ESI): calculated = 690.37487 $[M+H]^+$, found = 690.37549 $[M+H]^+$, err [ppm] = 0.90.

RP-HPLC (0 – 100% B, 1.5 mL/min, 20 min, 220 nm): t_R (**13f**) = 21.33 min (98% Purity)

(2R,5S,12R)-12-cyclohexyl-2-[2-(3,4-dimethoxyphenyl)ethyl]-16-methyl-3,19-dioxa-10,13,16-triazatricyclo[18.3.1.0^{5,10}]tetracosa-1(24),20,22-triene-4,11,14,17-tetrone

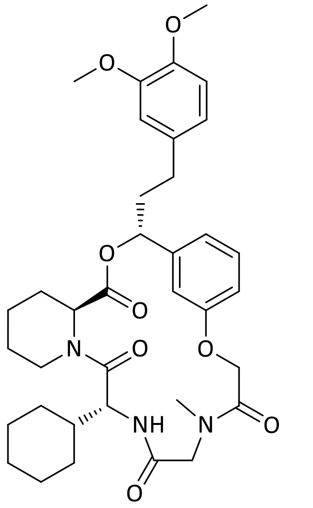

Chemical Formula: $C_{36}H_{47}N_3O_8$
Exact Mass: 649,34
Molecular Weight: 649,77

13g

Starting materials: Resin: (0.24 mmol), 1st AA: Fmoc-D-Chg-OH, 2nd AA: Fmoc-Gly-OH, Procedure as described at p. 32.

prep-HPLC (60 – 100% B, 10 mL/min, 15 min, 254 nm): t_R (**13g**) = 4.60 min.

Silica column chromatography: DCM + 2% MeOH, v/v.

Yield 30 mg (19%).

^{1}H-NMR (500 MHz, CDCl$_3$): δ 7.23 (t, J = 7.9 Hz, 1H), 6.96 (s, 1H), 6.80 – 6.73 (m, 3H), 6.69 – 6.61 (m, 2H), 5.73 – 5.67 (m, 1H), 5.41 – 5.35 (m, 1H), 4.89 (t, J = 7.8 Hz, 1H), 4.72 (d, J = 12.6 Hz, 1H), 4.49 (d, J = 12.6 Hz, 1H), 4.06 (q, J = 17.3 Hz, 2H), 3.92 – 3.85 (m, 1H), 3.85 (s, 3H), 3.84 (s, 3H), 3.28 – 3.18 (m, 1H), 3.07 (s, 3H), 2.62 – 2.51 (m, 2H), 2.25 – 2.02 (m, 2H), 1.83 – 1.62 (m, 12H), 1.54 – 1.38 (m, 1H), 1.30 – 0.98 (m, 5H).

^{13}C-NMR (126 MHz, CDCl$_3$): δ 171.69, 170.83, 168.72, 167.77, 157.99, 149.01, 147.46, 142.02, 133.74, 129.52, 120.27, 119.53, 114.01, 111.90, 111.72, 111.47, 75.87, 68.30, 56.07, 56.01, 53.72, 53.55, 53.08, 52.11, 43.94, 42.00, 37.99, 35.78, 30.84, 30.15, 28.60, 26.19, 26.14, 26.07, 25.29, 19.77.

LC-MS (50-100% B, 19 min): t_R (**13g**) = 7.07 min, m/z : calculated = 650.34 [M+H]$^+$, found = 650.51 [M+H]$^+$.

HRMS (ESI): calculated = 650.34359 [M+H]$^+$, found = 650.34345 [M+H]$^+$, err [ppm] = 0.22.

RP-HPLC (50 – 100% B, 1.5 mL/min, 15 min, 220 nm): t_R (**13g**) = 6.77 min (99% Purity)

((2R,5S,12R)-12-cyclohexyl-2-[2-(3,4-dimethoxyphenyl)ethyl]-15,15,16-trimethyl-3,19-dioxa-10,13,16-triazatricyclo[18.3.1.$0^{5,10}$]tetracosa-1(24),20,22-triene-4,11,14,17-tetrone

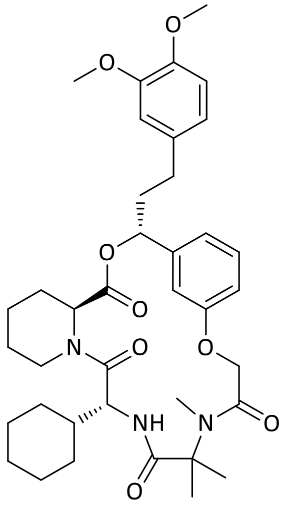

Chemical Formula: $C_{38}H_{51}N_3O_8$

Exact Mass: 677,37

Molecular Weight: 677,83

13h

Starting materials: Resin: (0.15 mmol), 1st AA: Fmoc-D-Chg-OH, 2nd AA: Fmoc-AIB-OH, Procedure as described at p. 32.

prep-HPLC (70 – 90% B, 10 mL/min, 25 min, 254 nm): t_R (**13h**) = 16.25 min.

Yield 15 mg (21%).

LC-MS (50-100% B, 19 min): t_R (**13h**) = 7.64 min, m/z: calculated = 678.37 $[M+H]^+$, found = 678.41 $[M+H]^+$.

RP-HPLC (50 – 100% B, 1.5 mL/min, 20 min, 254 nm): t_R (**13h**) = 9.34 min (89% Purity)

(9R,12R,19S,22R)-12-cyclohexyl-22-[2-(3,4-dimethoxyphenyl)ethyl]-2,21-dioxa-5,11,14-triazatetracyclo[21.3.1.0^{5,9}.0^{14,19}]heptacosa-1(26),23(27),24-triene-4,10,13,20-tetrone

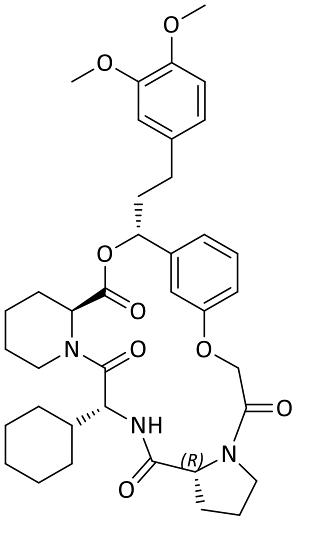

Chemical Formula: $C_{38}H_{49}N_3O_8$

Exact Mass: 675,35

Molecular Weight: 675,81

13i

Starting materials: Resin: (0.15 mmol), 1st AA: Fmoc-D-Chg-OH, 2nd AA: Fmoc-D-Pro-OH, Procedure as described at p. 32.

Silica column chromatography: DCM/MeOH, 9/1, v/v.

prep-HPLC (65 – 68% B, 10 mL/min, 14 min, 254 nm): t_R (**13i**) = 11.80 min.

Yield 7 mg (7%).

LC-MS (0-100% B, 19 min): t_R (**13i**) = 12.38 min, m/z : calculated = 676.36 [M+H]$^+$, found = 676.18 [M+H]$^+$.

HRMS (ESI): calculated = 676.35924 [M+H]$^+$, found = 676.35889 [M+H]$^+$, err [ppm] = 0.52.

RP-HPLC (0 – 100% B, 1.5 mL/min, 20 min, 220 nm): t_R (**13i**) = 20.25 min (99% Purity)

(2R,5S,12R,15S)-12-cyclohexyl-2-[2-(3,4-dimethoxyphenyl)ethyl]-15-methyl-3,19-dioxa-10,13,16-triazatricyclo[18.3.1.0⁵,¹⁰]tetracosa-1(24),20,22-triene-4,11,14,17-tetrone

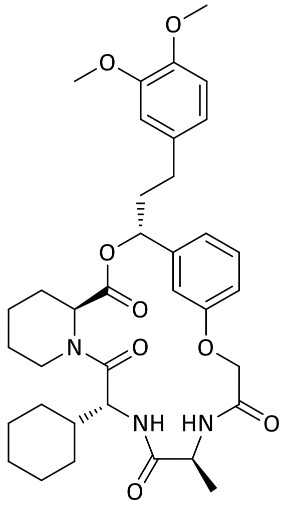

Chemical Formula: $C_{36}H_{47}N_3O_8$
Exact Mass: 649,34
Molecular Weight: 649,77

13j

Starting materials: Resin: (0.15 mmol), 1st AA: Fmoc-D-Chg-OH, 2nd AA: Fmoc-L-Ala-OH, Procedure as described at p. 32.

Silica column chromatography: DCM/MeOH, 8/2, v/v.
prep-HPLC (80 – 100% B, 10 mL/min, 10 min, 254 nm): t_R (**13j**) = 11.80 min.
Yield 27 mg (28%).
^{1}H-NMR (800 MHz, DMSO-d_6 + CD_2Cl_2): δ 8.30 (d, J = 9.0 Hz, 1H), 7.68 (d, J = 8.5 Hz, 1H), 7.29 – 7.25 (m, 1H), 6.96 (ddd, J = 1.0, 2.6, 8.2 Hz, 1H), 6.93 – 6.89 (m, 1H), 6.83 (d, J = 8.1 Hz, 1H), 6.75 (d, J = 2.0 Hz, 1H), 6.74 – 6.71 (m, 1H), 6.65 (dd, J = 2.0, 8.1 Hz, 1H), 5.73 (t, J = 7.1 Hz, 1H), 5.23 – 5.20 (m, 1H), 4.68 (d, J = 16.1 Hz, 1H), 4.64 – 4.58 (m, 1H), 4.46 (t, J = 9.0 Hz, 1H), 4.42 (d, J = 16.1 Hz, 1H), 3.99 – 3.92 (m, 1H), 3.73 (s, 3H), 3.71 (s, 3H), 2.64 – 2.56 (m, 1H), 2.48 – 2.37 (m, 2H), 2.15 – 2.07 (m, 2H), 2.06 – 1.99 (m, 1H), 1.74 – 1.60 (m, 5H), 1.60 – 1.54 (m, 2H), 1.52 – 1.42 (m, 2H), 1.40 – 1.21 (m, 2H), 1.16 (d, J = 6.8 Hz, 3H), 1.14 – 1.07 (m, 2H), 0.97 – 0.80 (m, 2H).
^{13}C-NMR (201 MHz, DMSO-d_6 + CD_2Cl_2): δ 171.15, 170.52, 170.43, 167.86, 158.41, 148.63, 147.05, 140.21, 133.18, 129.53, 121.86, 119.97, 117.71, 112.25, 111.87, 110.35, 75.05, 68.11, 55.51, 55.34, 54.90, 51.85, 51.66, 46.78, 43.37, 35.15, 30.72, 29.31, 27.98, 26.17, 25.77, 25.41, 25.36, 25.10, 20.19, 18.21.
LC-MS (0-100% B, 19 min): t_R (**13j**) = 12.34 min, m/z : calculated = 650.34 $[M+H]^+$, found = 650.07 $[M+H]^+$.
HRMS (ESI): calculated = 650.34359 $[M+H]^+$, found = 650.34358 $[M+H]^+$, err [ppm] = 0.01.
RP-HPLC (0 – 100% B, 1.5 mL/min, 20 min, 220 nm): t_R (**13j**) = 20.72 min (98% Purity)

(9S,12R,19S,22R)-12-cyclohexyl-22-[2-(3,4-dimethoxyphenyl)ethyl]-2,21-dioxa-5,11,14-triazatetracyclo[21.3.1.0^{5,9}.0^{14,19}]heptacosa-1(26),23(27),24-triene-4,10,13,20-tetrone

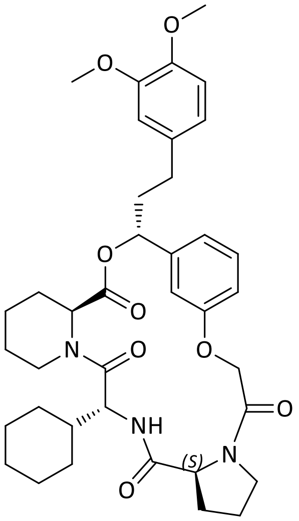

Chemical Formula: $C_{38}H_{49}N_3O_8$
Exact Mass: 675,35
Molecular Weight: 675,81

13k

Starting materials: Resin: (0.15 mmol), 1st AA: Fmoc-D-Chg-OH, 2nd AA: Fmoc-L-Pro-OH, Procedure as described at p. 32.

Silica column chromatography: DCM/MeOH, 9/1, v/v.

prep-HPLC (65 – 68% B, 10 mL/min, 14 min, 254 nm): t_R (**13k**) = 11.80 min.

Yield 4 mg (4%).

LC-MS (0-100% B, 19 min): t_R (**13k**) = 12.03 min, m/z : calculated = 676.36 [M+H]$^+$, found = 676.18 [M+H]$^+$.

HRMS (ESI): calculated = 676.35924 [M+H]$^+$, found = 676.35941 [M+H]$^+$, err [ppm] = 0.24.

RP-HPLC (0 – 100% B, 1.5 mL/min, 20 min, 220 nm): t_R (**13k**) = 19.82 min (99% Purity)

(2R,5S,12R)-12-cyclohexyl-2-[2-(3,4-dimethoxyphenyl)ethyl]-3,20-dioxa-10,13,17-triazatricyclo[19.3.1.0⁵,¹⁰]pentacosa-1(25),21,23-triene-4,11,14,18-tetrone

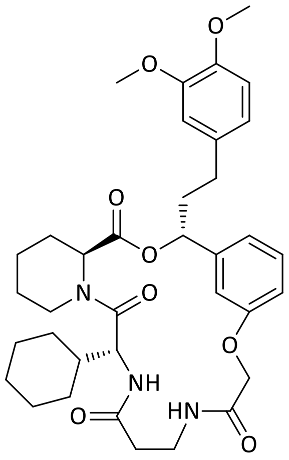

Chemical Formula: $C_{36}H_{47}N_3O_8$
Exact Mass: 649,34
Molecular Weight: 649,77

13l

Starting materials: Resin: (0.13 mmol), 1st AA: Fmoc-D-Chg-OH, 2nd AA: Fmoc-β-Ala-OH, Procedure as described at p. 32.

Silica column chromatography: DCM/MeOH, 9/1, v/v.

prep-HPLC (80 – 100% B, 10 mL/min, 10 min, 254 nm): t_R (**13l**) = 9.60 min.

Yield 14 mg (16%).

^{1}H-NMR (800 MHz, DMSO-d_6 + CD_2Cl_2): δ 7.93 (d, J = 9.1 Hz, 1H), 7.82 – 7.78 (m, 1H), 7.24 (t, J = 7.9 Hz, 1H), 6.89 – 6.86 (m, 1H), 6.86 – 6.82 (m, 2H), 6.77 (d, J = 2.0 Hz, 1H), 6.74 – 6.71 (m, 1H), 6.69 (dd, J = 2.0, 8.1 Hz, 1H), 5.58 (dd, J = 5.3, 8.2 Hz, 1H), 5.17 – 5.13 (m, 1H), 4.71 – 4.67 (m, 1H), 4.46 (d, J = 15.4 Hz, 1H), 4.40 (d, J = 15.4 Hz, 1H), 4.03 – 3.97 (m, 1H), 3.74 (s, 3H), 3.71 (s, 3H), 3.59 – 3.53 (m, 1H), 3.18 – 3.13 (m, 1H), 3.13 – 3.06 (m, 1H), 2.58 – 2.52 (m, 1H), 2.48 – 2.40 (m, 2H), 2.32 – 2.27 (m, 1H), 2.10 – 2.05 (m, 1H), 2.05 – 1.98 (m, 2H), 1.77 – 1.69 (m, 3H), 1.69 – 1.64 (m, 1H), 1.64 – 1.56 (m, 5H), 1.42 – 1.32 (m, 1H), 1.28 – 1.06 (m, 5H), 1.05 – 0.92 (m, 2H).

^{13}C-NMR (201 MHz, DMSO-d_6 + CD_2Cl_2): δ 170.89, 170.83, 170.40, 167.51, 158.36, 148.65, 147.05, 141.88, 133.26, 129.34, 120.09, 119.68, 114.92, 112.28, 111.89, 111.34, 74.62, 67.48, 55.49, 55.36, 54.90, 52.76, 51.86, 43.31, 37.31, 34.31, 34.24, 30.39, 29.65, 27.58, 26.38, 25.81, 25.65, 25.59, 24.76, 20.19.

LC-MS (0-100% B, 19 min): t_R (**13l**) = 11.97 min, m/z : calculated = 650.35 $[M+H]^+$, found = 650.04 $[M+H]^+$.

HRMS (ESI): calculated = 650.34359 $[M+H]^+$, found = 650.34363 $[M+H]^+$, err [ppm] = 0.05.

RP-HPLC (0 – 100% B, 1.5 mL/min, 20 min, 220 nm): t_R (**13l**) = 19.73 min (99% Purity)

(2R,5S,12R,15R,19R)-12-cyclohexyl-2-[2-(3,4-dimethoxyphenyl)ethyl]-3,23-dioxa-10,13,20-triazatetracyclo[22.3.1.0⁵,¹⁰.0¹⁵,¹⁹]octacosa-1(28),24,26-triene-4,11,14,21-tetrone

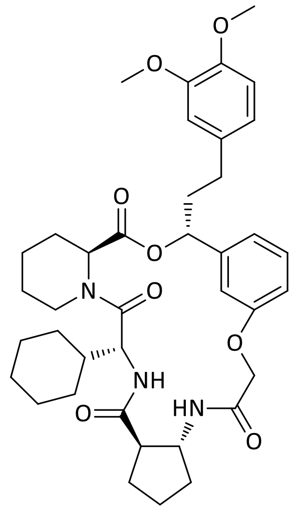

Chemical Formula: $C_{39}H_{51}N_3O_8$
Exact Mass: 689,37
Molecular Weight: 689,84

13m

Starting materials: Resin: (0.14 mmol), 1st AA: Fmoc-D-Chg-OH, 2nd AA: (1R,2R)-Fmoc-2-amino-1-cyclopentanecarboxylic acid, Procedure as described at p. 32.

prep-HPLC (90 – 96% B, 10 mL/min, 10 min, 254 nm): t_R (**13m**) = 5.10 min.
Yield 3 mg (3%).
LC-MS (0-100% B, 19 min): t_R (**13m**) = 12.31 min, m/z : calculated = 690.37 [M+H]+, found = 690.32 [M+H]+.
HRMS (ESI): calculated = 690.37489 [M+H]+, found = 690.37473 [M+H]+, err [ppm] = 0.23.
RP-HPLC (0 – 100% B, 1.5 mL/min, 20 min, 220 nm): t_R (**13m**) = 20.33 min (99% Purity)

(2R,5S,12R)-12-cyclohexyl-2-[2-(3,4-dimethoxyphenyl)ethyl]-3,21-dioxa-10,13,18-triazatricyclo[20.3.1.0⁵,¹⁰]hexacosa-1(26),22,24-triene-4,11,14,19-tetrone

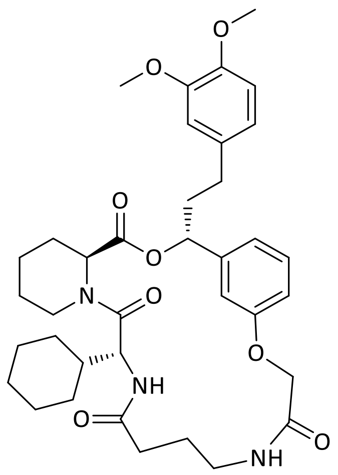

Chemical Formula: $C_{37}H_{49}N_3O_8$
Exact Mass: 663,35
Molecular Weight: 663,80

13n

Starting materials: Resin: (0.24 mmol), 1st AA: Fmoc-D-Chg-OH, 2nd AA: Fmoc-GABA-OH, Procedure as described at p. 32.

Silica column chromatography: DCM/MeOH, 95/5, v/v.

prep-HPLC (80 – 100% B, 10 mL/min, 20 min, 254 nm): t_R (**13n**) = 10.20 min.

Yield 8 mg (5%).

LC-MS (0-100% B, 19 min): t_R (**13n**) = 11.72 min, m/z : calculated = 664.35 $[M+H]^+$, found = 664.31 $[M+H]^+$.

HRMS (ESI): calculated = 664.35924 $[M+H]^+$, found = 664.35929 $[M+H]^+$, err [ppm] = 0.07.

RP-HPLC (0 – 100% B, 1.5 mL/min, 20 min, 220 nm): t_R (**13n**) = 19.23 min (99% Purity)

(2R,5S,12R,15S,19R)-12-cyclohexyl-2-[2-(3,4-dimethoxyphenyl)ethyl]-3,23-dioxa-10,13,20-triazatetracyclo[22.3.1.0^{5,10}.0^{15,19}]octacosa-1(28),24,26-triene-4,11,14,21-tetrone

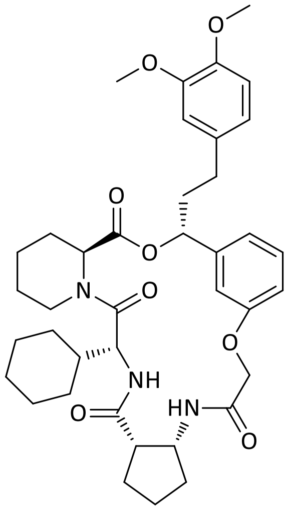

Chemical Formula: $C_{39}H_{51}N_3O_8$
Exact Mass: 689,37
Molecular Weight: 689,84

13o

Starting materials: Resin: (0.07 mmol), 1st AA: Fmoc-D-Chg-OH, 2nd AA: (1S,2R)-Fmoc-2-amino-1-cyclopentanecarboxylic acid, Procedure as described at p. 32.

prep-HPLC (90 – 96% B, 10 mL/min, 10 min, 254 nm): t_R (**13o**) = 5.10 min.

Yield 2 mg (4%).

LC-MS (0-100% B, 19 min): t_R (**13o**) = 12.69 min, m/z : calculated = 690.37 $[M+H]^+$, found = 690.28 $[M+H]^+$.

HRMS (ESI): calculated = 690.37489 $[M+H]^+$, found = 690.37455 $[M+H]^+$, err [ppm] = 0.50.

RP-HPLC (0 – 100% B, 1.5 mL/min, 20 min, 220 nm): t_R (**13o**) = 21.03 min (99% Purity)

9.4. Tracer synthesis

(E)-1-(3,5-dihydroxyphenyl)-3-(3,4-dimethoxyphenyl)prop-2-en-1-one

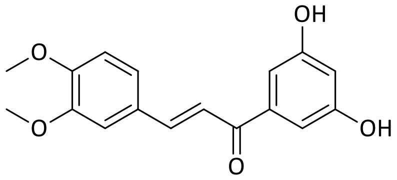

Chemical Formula: $C_{17}H_{16}O_5$
Exact Mass: 300,10
Molecular Weight: 300,31

15

3,5-Dihydroxyacetophenon (6.00 g, 39 mmol, 1.0 eq) and 3,4-dimethoxybenzaldehyde (6.55 g, 39 mol, 1.0 eq) are dissolved in 120 mL EtOH and sparged with argon for 20 min. The solution is cooled to 0°C and cooled NaOH (10.40 g, 350 mmol, 9.0 eq) dissolved in 120 mL H_2O is slowly added in 10 min. The reaction is stirred under argon at slowly rising temperature to r.t. overnight. The mixture is acidified with conc. HCl and extracted with EE (3×150 mL). The combined organic layers are dried with $MgSO_4$, filtered and the solvent removed under reduced pressure. The crude product is purified by silica filtration (EE). The pure product **20** is obtained as yellow foam.

Yield 8.75 g (74%).
TLC (CH/EE, 1/1): R_f (**15**) = 0.22.
^{1}H-NMR (500 MHz, DMSO-*d*$_6$): δ 9.61 (s, 2H), 7.67 – 7.60 (m, 2H), 7.50 (d, *J* = 2.0 Hz, 1H), 7.34 (dd, *J* = 2.0, 8.4 Hz, 1H), 7.03 – 6.93 (m, 3H), 6.80 (d, *J* = 2.2 Hz, 1H), 6.51 (t, *J* = 2.2 Hz, 1H), 3.85 (s, 3H), 3.81 (s, 3H).
^{13}C-NMR (126 MHz, DMSO-*d*$_6$): δ 197.74, 189.25, 158.69, 151.26, 149.10, 144.22, 140.08, 138.89, 127.59, 123.83, 120.08, 111.60, 110.72, 107.20, 106.99, 106.57, 106.24, 59.81, 55.76, 55.61, 26.76, 20.76, 14.10.
LC-MS (0-100% B, 19 min): t_R (**15**) = 9.08 min, m/z : calculated = 301.10 $[M+H]^+$, found = 301.22 $[M+H]^+$.
RP-HPLC (0 – 100% B, 1.5 mL/min, 20 min, 254 nm): t_R (**15**) = 10.95 min (83% Purity).

1-(3,5-dihydroxyphenyl)-3-(3,4-dimethoxyphenyl)propan-1-one

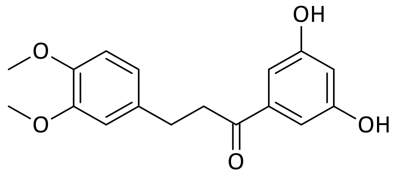

Chemical Formula: $C_{17}H_{18}O_5$
Exact Mass: 302,12
Molecular Weight: 302,32

16

Zn powder (1.4 g, 21 mmol, 5.0 eq) and NH_4Cl (5.5 g, 126 mmol, 30.0 eq) are added to a flask and suspended in 50 mL MeOH. **15** (1.22 g, 4 mmol, 1.0 eq) is dissolved in 30 mL MeOH and added dropwise to the vigorously stirring suspension in 1.5 h. After complete addition, the mixture is filtered and washed with MeOH. The solvent is removed under reduced pressure, then the solid dissolved in 100 mL H_2O and extracted with EE (3×100 mL). The combined organic layers are dried with $MgSO_4$, filtered and the solvent removed under reduced pressure. The crude product is purified by column chromatography (CH/EE, 1/1). The pure product **16** is obtained as beige-white solid.

Yield 350 mg (28%).
TLC (CH/EE, 1/1): R_f (**16**) = 0.25.
^{1}H-NMR (500 MHz, DMSO-*d*$_6$) δ 9.54 (s, 2H), 6.86 (d, *J* = 2.0 Hz, 1H), 6.83 (d, *J* = 8.2 Hz, 1H), 6.80 (d, *J* = 2.2 Hz, 2H), 6.74 (dd, *J* = 2.0, 8.2 Hz, 1H), 6.44 (t, *J* = 2.2 Hz, 1H), 3.73 (s, 3H), 3.70 (s, 3H), 3.18 (dd, *J* = 7.0, 8.0 Hz, 2H), 2.83 (t, *J* = 7.5 Hz, 2H).

^{13}C-NMR (126 MHz, DMSO) δ 199.02, 158.55, 148.61, 147.04, 138.65, 133.71, 120.02, 112.44, 111.93, 107.01, 105.87, 55.54, 55.41, 29.27.

LC-MS (0-100% B, 19 min): t_R (**16**) = 9.23 min, m/z : calculated = 303.12 $[M+H]^+$, found = 302.92 $[M+H]^+$.

RP-HPLC (0 – 100% B, 1.5 mL/min, 20 min, 254 nm): t_R (**16**) = 10.61 min (99% Purity).

(R)-5-(3-(3,4-dimethoxyphenyl)-1-hydroxypropyl)benzene-1,3-diol

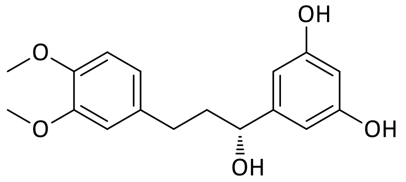

Chemical Formula: $C_{17}H_{20}O_5$
Exact Mass: 304,13
Molecular Weight: 304,34

17

16 (2.10 g, 6.95 mmol, 1.0 eq) is dissolved in 50 mL THF and added to an autoclave (Roth, model II). The solution is sparged with argon for 10 min, then 100 mL iPrOH is added and the solution further sparged with argon for 5 min. $RuCl_2$[(*S*)-(DM-SEGPHOS)][(*S*)-DAIPEN] (84 mg, 0.07 mmol, 0.01 eq) and 1 M KOtBu in tBuOH (7 mL, 7 mmol, 1.0 eq) is added and the autoclave closed, then flushed 3x with H_2 and finally 10 bar H_2 applied. After reaction overnight, the mixture is transferred to a flask and the solvent is removed under reduced pressure. The crude product is dissolved in 200 mL EE and washed with 100 mL sat. NH_4Cl solution. The aqueous phase is extracted with EE (3×100 ml). The combined organic layers are dried with $MgSO_4$, filtered and the solvent removed under reduced pressure. The crude product is purified by column chromatography (CH/EE, 1/2). The pure product **17** is obtained as a white solid.

Yield 1.37 g (65%).

TLC (CH/EE, 1/1): R_f (**17**) = 0.14.

^{1}H-NMR (500 MHz, DMSO-*d*$_6$) δ 9.03 (s, 2H), 6.83 (d, *J* = 8.2 Hz, 1H), 6.76 (d, *J* = 2.0 Hz, 1H), 6.68 (dd, *J* = 2.0, 8.1 Hz, 1H), 6.20 (d, *J* = 2.2 Hz, 2H), 6.06 (t, *J* = 2.2 Hz, 1H), 5.03 (d, *J* = 4.3 Hz, 1H), 4.32 (dt, *J* = 4.8, 7.4 Hz, 1H), 3.73 (s, 3H), 3.70 (s, 3H), 2.62 – 2.51 (m, 2H), 2.47 (s, 0H), 1.79 (ddt, *J* = 5.6, 9.0, 11.9 Hz, 2H).

^{13}C-NMR (126 MHz, DMSO) δ 158.04, 148.64, 148.48, 146.85, 134.70, 119.91, 112.25, 111.97, 103.90, 100.83, 71.71, 55.54, 55.39, 40.98, 30.64.

LC-MS (0-100% B, 19 min): t_R (**17**) = 8.53 min, m/z : calculated = 287.11 $[M-OH]^+$, found = 287.12 $[M-OH]^+$.

RP-HPLC (0 – 100% B, 1.5 mL/min, 20 min, 254 nm): t_R (**17**) = 9.06 min (98% Purity).

(R)-3-(3-(3,4-dimethoxyphenyl)-1-hydroxypropyl)-5-(trityloxy)phenol

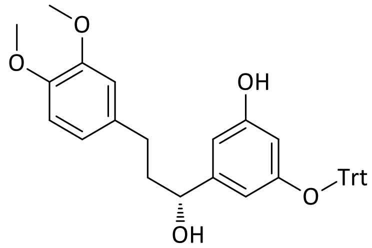

Chemical Formula: $C_{36}H_{34}O_5$
Exact Mass: 546,24
Molecular Weight: 546,65

18

17 (680 mg, 2.23 mmol, 1.0 eq) is dissolved in 50 mL dry MeCN and added to a dried flask under argon atmosphere. Then K_2CO_3 (308 mg, 2.23 mmol, 1.0 eq) and trityl chloride (466 mg, 1.67 mmol, 0.75 eq) are added. The reaction is stirred at r.t. over night. The

mixture is filtered and the solvent removed. The crude product is purified by column chromatography (CH/EE, 2/1 gradient to EE). The pure product **18** is obtained as yellow solid.

Yield 431 mg (47%).
TLC (CH/EE, 2/1): R_f (**18**) = 0.20.
^{1}H-NMR ^{1}H NMR (500 MHz, DMSO-*d*$_6$) δ 9.01 (s, 1H), 7.41 – 7.38 (m, 6H), 7.30 (dd, *J* = 7.0, 8.5 Hz, 7H), 7.24 – 7.19 (m, 3H), 6.83 (d, *J* = 8.1 Hz, 1H), 6.68 (d, *J* = 2.0 Hz, 1H), 6.58 (dd, *J* = 2.0, 8.2 Hz, 1H), 6.22 (t, *J* = 1.7 Hz, 1H), 6.06 (d, *J* = 2.0 Hz, 1H), 5.98 (t, *J* = 2.2 Hz, 1H), 4.97 (d, *J* = 4.4 Hz, 1H), 4.14 (dt, *J* = 4.7, 7.6 Hz, 1H), 3.72 (d, *J* = 5.2 Hz, 6H), 2.32 (dddd, *J* = 6.2, 9.7, 13.9, 23.5 Hz, 2H), 1.64 – 1.47 (m, 2H).
^{13}C-NMR (126 MHz, DMSO-*d*$_6$): δ 156.99, 156.22, 148.55, 147.35, 146.80, 143.90, 134.58, 128.33, 127.69, 127.04, 119.84, 112.22, 111.92, 109.06, 106.07, 105.93, 89.16, 71.36, 59.70, 55.53, 55.37, 40.80, 30.79, 20.71, 14.04.
LC-MS (50-100% B, 19 min): t_R (**18**) = 8.78 min, m/z : calculated = 569.23 $[M+Na]^+$, found = 596.11 $[M+Na]^+$.

allyl 2-bromoacetate

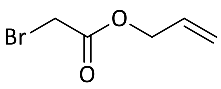

Chemical Formula: $C_5H_7BrO_2$
Exact Mass: 177,96
Molecular Weight: 179,01

19

Allyl alcohol (1.00 mL, 15 mmol, 1.0 eq) is dissolved in dry DCM in a dried flask under argon atmosphere. Pyridine (1.18 mL, 15 mmol, 1.0 eq) is added and the flask cooled to 0°C for 15 min. Then bromoacetyl bromide (1.40 mL, 16 mmol, 1.1 eq) is added dropwise in 20 min. After further 20 min stirring at 0°C the ice bath is removed and the reaction stirred at r.t. for 30 min. Finally, the reaction is quenched by addition of 30 mL H_2O, then extracted with DCM (3×50 mL), the combined organic layers washed with brine and dried with $MgSO_4$, filtered and concentrated under reduced pressure. The crude product **19** is used without further purification.

Yield 2.61 g (quant.).
TLC (CH/EE, 4/1, v/v): R_f (**19**) = 0.66.
^{1}H-NMR (300 MHz, $CDCl_3$): δ 6.06 – 5.82 (m, 1H), 5.44 – 5.23 (m, 2H), 4.67 (dd, *J* = 2.3, 5.8 Hz, 2H), 3.92 – 3.81 (m, 2H).
^{13}C-NMR (75 MHz, $CDCl_3$): δ 167.05, 131.35, 119.33, 66.88, 25.82.
EI-MS (303 K): m/z : calculated = 123/121 $[M\text{-}Oallyl]^+$, 99 $[M\text{-}HBr]^+$, 95/93 $[BrCH_2]^+$, 85 $[M\text{-}BrCH_2]^+$, found = 123/121 $[M\text{-}Oallyl]^+$, 99 $[M\text{-}HBr]^+$, 95/93 $[BrCH_2]^+$, 85 $[M\text{-}BrCH_2]^+$.

(R)-allyl 2-(3-(3-(3,4-dimethoxyphenyl)-1-hydroxypropyl)-5-(trityloxy)phenoxy)acetate

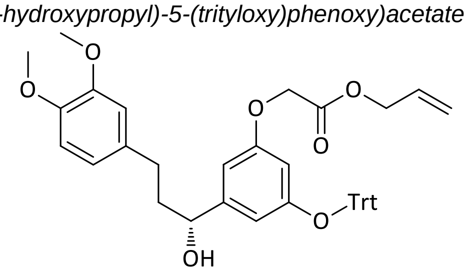

Chemical Formula: $C_{41}H_{40}O_7$
Exact Mass: 644,28
Molecular Weight: 644,75

20

18 (1000 mg, 1.83 mmol, 1.0 eq) is dissolved in 50 mL MeCN. K_2CO_3 (1000 mg, 7.32 mmol, 4.0 eq) and **19** (570 mg, 3.20 mmol, 1.75 eq) is added. The reaction is stirred at r.t. night. After complete conversion, the suspension is filtered, washed with MeCN and the

solvent removed under reduced pressure. The crude product is purified by column chromatography (CH/EE, 3/1 then 2/1). The pure product **20** is obtained as yellow solid.

Yield 910 mg (77%).

TLC (CH/EE, 1/1): R_f(**20**) = 0.57.

^{1}H-NMR (500 MHz, DMSO-*d*$_6$) δ 7.41 – 7.37 (m, 6H), 7.30 (dd, *J* = 7.0, 8.5 Hz, 6H), 7.25 – 7.20 (m, 3H), 6.83 (d, *J* = 8.2 Hz, 1H), 6.68 (d, *J* = 2.0 Hz, 1H), 6.58 (dd, *J* = 2.0, 8.2 Hz, 1H), 6.36 (dd, *J* = 1.2, 2.5 Hz, 1H), 6.24 (t, *J* = 1.7 Hz, 1H), 6.07 (t, *J* = 2.3 Hz, 1H), 5.86 (ddt, *J* = 5.4, 10.7, 17.3 Hz, 1H), 5.30 – 5.17 (m, 2H), 5.06 (d, *J* = 4.5 Hz, 1H), 4.57 – 4.53 (m, 4H), 4.21 (dt, *J* = 4.9, 7.6 Hz, 1H), 3.73 (s, 3H), 3.71 (s, 3H), 2.32 (dddd, *J* = 6.1, 9.7, 13.9, 23.6 Hz, 2H), 1.64 – 1.48 (m, 1H).

^{13}C-NMR (126 MHz, DMSO-*d*$_6$): δ 168.21, 157.39, 156.29, 148.55, 147.73, 146.81, 143.63, 134.49, 132.09, 128.29, 127.78, 127.16, 119.84, 117.94, 113.41, 112.22, 111.91, 111.10, 105.12, 104.90, 89.52, 71.27, 64.74, 64.55, 59.69, 55.53, 55.38, 40.76, 30.75.

LC-MS (70-100% B, 19 min): t_R(**20**) = 6.33 min, m/z : calculated = 667.27 $[M+Na]^+$, found = 667.16 $[M+Na]^+$.

(S)-1-((9H-fluoren-9-yl)methyl) 2-((R)-1-(3-(2-(allyloxy)-2-oxoethoxy)-5-hydroxyphenyl)-3-(3,4-dimethoxyphenyl)propyl) piperidine-1,2-dicarboxylate

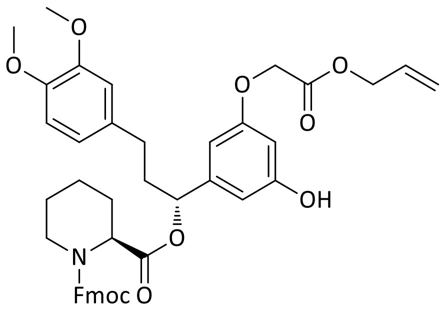

Chemical Formula: $C_{43}H_{45}NO_{10}$
Exact Mass: 735,30
Molecular Weight: 735,82

21

20 (910 mg, 1.41 mmol, 1.0 eq) and Fmoc-S-pipecolate (546 mg, 1.55 mmol, 1.1 eq) are dissolved in 50 mL dry DCM and cooled to 0°C for 15 min. DMAP (57 mg, 0.47 mmol, 0.3 eq) is added and stirred until dissolved, then DCC (320 mg, 1.55 mmol, 1.1 eq) is added. The mixture is stirred for 15 min under cooling. Finally, the ice bath is removed and the reaction stirred overnight at r.t. The reaction mixture is filtered, washed with DCM and the solvent removed under reduced pressure. The crude product is dissolved in 30 mL DCM + 1% TFA and stirred for 5 min. The solvent is removed under reduced pressure and the crude product purified by silica column chromatography (CH/EE, 2/1) and the pure product **21** is obtained as colorless oil.

Yield 1.05 g (92%).

TLC (CH/EE, 2/1, v/v): R_f(**21**) = 0.30.

^{1}H-NMR (500 MHz, Chloroform-*d*): δ 7.79 – 7.72 (m, 1H), 7.71 (d, J = 7.6 Hz, 1H), 7.57 (dd, J = 7.6, 11.5 Hz, 1H), 7.46 (dd, J = 7.5, 26.6 Hz, 1H), 7.34 (ddt, J = 7.6, 16.1, 33.4 Hz, 3H), 7.20 (t, J = 7.5 Hz, 1H), 6.77 – 6.70 (m, 1H), 6.68 – 6.53 (m, 2H), 6.45 – 6.41 (m, 2H), 6.30 (d, J = 17.0 Hz, 1H), 5.94 – 5.82 (m, 1H), 5.74 – 5.62 (m, 1H), 5.33 – 5.27 (m, 1H), 5.23 (d, J = 10.5 Hz, 1H), 5.04 – 4.86 (m, 1H), 4.68 – 4.63 (m, 2H), 4.57 (s, 1H), 4.50 (s, 1H), 4.48 – 4.41 (m, 1H), 4.38 – 4.22 (m, 2H), 4.06 (d, J = 16.4 Hz, 1H), 3.87 – 3.74 (m, 6H), 3.22 – 3.13 (m, 1H), 2.90 (t, J = 13.1 Hz, 0H), 2.62 – 2.38 (m, 2H), 2.34 – 2.25 (m, 1H), 2.21 – 1.90 (m, 1H), 1.72 (t, J = 14.4 Hz, 4H), 1.52 – 1.39 (m, 1H), 1.31 (s, 1H).

^{13}C-NMR (126 MHz, Chloroform-*d*): δ 159.25, 149.07, 147.52, 143.94, 141.41, 131.53, 127.86, 127.22, 125.19, 120.27, 120.12, 119.25, 111.93, 111.84, 111.55, 107.01, 105.72, 105.27, 101.92, 76.16, 68.03, 66.00, 65.42, 56.09, 55.99, 55.09, 54.67, 47.37, 42.16, 38.08, 31.28, 31.21, 26.96, 24.88, 24.65, 20.80, 14.34.

LC-MS (50-100% B, 19 min): t_R(**21**) = 11.32 min, m/z : calculated = 753.33 $[M+NH_4]^+$, found = 753.73 $[M+NH_4]^+$.

(2R,5S,12R)-12-cyclohexyl-2-[2-(3,4-dimethoxyphenyl)ethyl]-22-hydroxy-15,15-dimethyl-3,19-dioxa-10,13,16-triazatricyclo[18.3.1.$0^{5,10}$]tetracosa-1(24),20,22-triene-4,11,14,17-tetrone

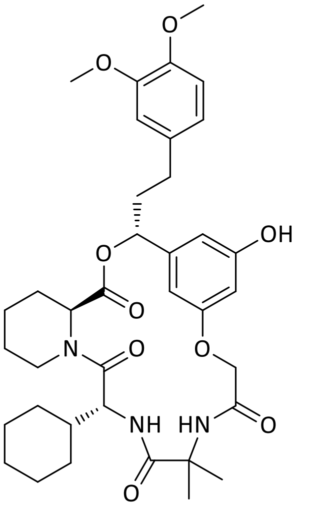

Chemical Formula: $C_{37}H_{49}N_3O_9$
Exact Mass: 679,35
Molecular Weight: 679,80

25

Starting materials: Resin: (0.66 mmol), 1st AA: Fmoc-D-Chg-OH, 2nd AA: Fmoc-Aib-OH, Procedure as described at p. 32.

Silica column chromatography: DCM/MeOH, 30/1, v/v.

TLC (DCM/MeOH, 30/1): R_f (**25**) = 0.25.

Yield 90 mg (20%).

^{1}H-NMR (500 MHz, $CDCl_3$): δ 7.78 (d, J = 8.8 Hz, 1H), 6.78 (d, J = 8.0 Hz, 1H), 6.68 (d, J = 2.0 Hz, 1H), 6.67 – 6.62 (m, 1H), 6.61 – 6.52 (m, 2H), 6.35 – 6.32 (m, 2H), 5.57 – 5.51 (m, 1H), 5.25 – 5.21 (m, 1H), 4.70 (s, 1H), 4.64 (d, J = 16.4 Hz, 1H), 4.53 (d, J = 16.3 Hz, 1H), 3.86 (s, 3H), 3.85 (s, 3H), 3.85 – 3.81 (m, 1H), 3.31 (t, J = 13.1 Hz, 1H), 2.57 – 2.48 (m, 2H), 2.29 – 2.19 (m, 1H), 2.17 – 2.10 (m, 1H), 2.08 – 1.99 (m, 1H), 1.77 (s, 3H), 1.77 – 1.61 (m, 8H), 1.53 – 1.44 (m, 1H), 1.43 (s, 3H), 1.31 – 1.16 (m, 3H), 1.18 – 1.09 (m, 1H), 1.08 – 0.93 (m, 2H).

^{13}C-NMR (126 MHz, $CDCl_3$): δ 174.09, 172.12, 171.65, 169.90, 158.71, 158.10, 149.04, 147.47, 142.80, 133.71, 120.26, 111.89, 111.54, 108.63, 102.77, 101.46, 76.57, 66.99, 58.74, 56.09, 56.02, 53.60, 52.49, 43.89, 41.38, 37.73, 31.22, 30.36, 28.17, 26.48, 26.24, 26.19, 26.11, 25.66, 25.31, 24.84, 19.97.

LC-MS (30-100% B, 19 min): t_R (**25**) = 10.92 min, m/z : calculated = 680.35 [M+H]$^+$, found = 680.49 [M+H]$^+$.

HRMS (ESI): calculated = 680.35416 [M+H]$^+$, found = 680.35433 [M+H]$^+$, err [ppm] = 0.25.

RP-HPLC (30 – 100% B, 1.5 mL/min, 20 min, 254 nm): t_R (**25**) = 12.05 min (99% Purity)

(2R,5S,12R)-12-cyclohexyl-2-[2-(3,4-dimethoxyphenyl)ethyl]-22-methoxy-15,15-dimethyl-3,19-dioxa-10,13,16-triazatricyclo[18.3.1.0^{5,10}]tetracosa-1(24),20,22-triene-4,11,14,17-tetrone

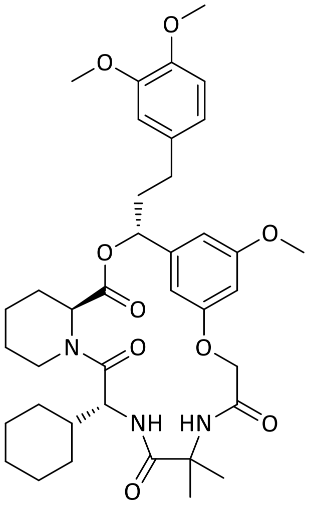

Chemical Formula: $C_{38}H_{51}N_3O_9$
Exact Mass: 693,36
Molecular Weight: 693,83

26

25 (5 mg, 0.01 mmol, 1.0 eq) is dissolved in 1 mL dry MeCN and K_2CO_3 (10 mg, 0.1 mmol, 10.0 eq) is added. Then MeI (5 µL, 0.1 mmol, 10.0 eq) is added and the mixture stirred at r.t. over night. The mixture is diluted with DCM and extracted 1x with 1 M $NaOH_{aq}$. The organic solvent is removed and the crude product purified by semi preparative HPLC.

prep-HPLC (70 – 100% B, 10 mL/min, 10 min, 254 nm): t_R (**26**) = 3.83 min.
Yield 1 mg (19%).
LC-MS (50-100% B, 19 min): t_R (**26**) = 9.07 min, m/z : calculated = 694.36 $[M+H]^+$, found = 694.47 $[M+H]^+$.
HRMS (ESI): calculated = 694.36981 $[M+H]^+$, found = 694.36967 $[M+H]^+$, err [ppm] = 0.20.

tert-butyl N-(2-{[(2R,5S,12R)-12-cyclohexyl-2-[2-(3,4-dimethoxyphenyl)ethyl]-15,15-dimethyl-4,11,14,17-tetraoxo-3,19-dioxa-10,13,16-triazatricyclo[18.3.1.0^{5,10}]tetracosa-1(24),20,22-trien-22-yl]oxy}ethyl)carbamate

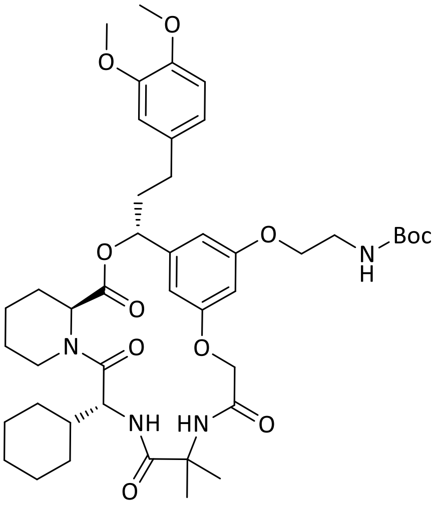

Chemical Formula: $C_{44}H_{62}N_4O_{11}$
Exact Mass: 822,44
Molecular Weight: 822,98

27

25 (33 mg, 0.048 mmol, 1.0 eq) is dissolved in 1 mL dry MeCN and Ag_2CO_3 (28 mg, 0.1 mmol, 2.0 eq) is added. Then *tert*-butyl (2-bromoethyl)carbamate (40 mg, 0.16 mmol, 3.0 eq) is added and the mixture stirred at 30°C over 3 d. The mixture is filtered and washed with MeCN. The organic solvent is removed, the crude product purified by semi preparative HPLC and pure **27** obtained.

prep-HPLC (50 – 100% B, 10 mL/min, 10 min, 254 nm): t_R (**27**) = 6.46 min.
Yield 18 mg (45%).
LC-MS (50-100% B, 19 min): t_R (**27**) = 13.68 min, m/z : calculated = 823.44 $[M+H]^+$, found = 823.32 $[M+H]^+$.

9-{2-carboxy-4(5)-[(2-{[(2R,5S,12R)-12-cyclohexyl-2-[2-(3,4-dimethoxyphenyl)ethyl]-15,15-dimethyl-4,11,14,17-tetraoxo-3,19-dioxa-10,13,16-triazatricyclo[18.3.1.0^{5,10}]tetracosa-1(24),20,22-trien-22-yl]oxy}ethyl)carbamoyl]phenyl}-6-(dimethylamino)-N,N-dimethyl-3H-xanthen-3-iminium

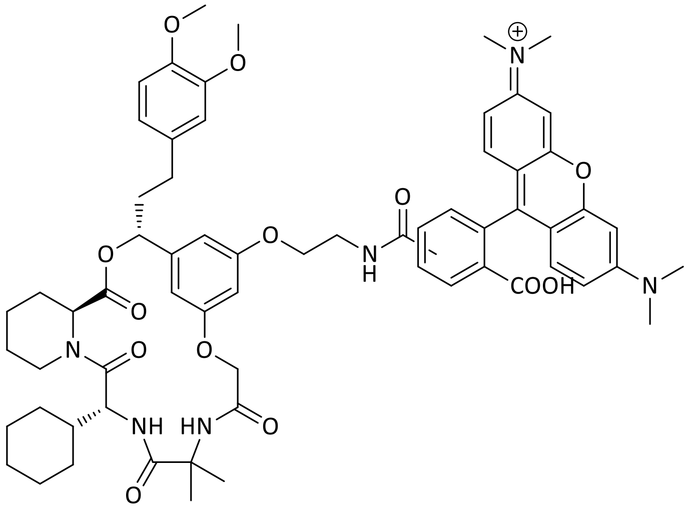

Chemical Formula: $C_{64}H_{75}N_6O_{13}^+$
Exact Mass: 1135,54
Molecular Weight: 1136,31

14a, b and **c**

27 (18 mg, 0.022 mmol, 1.0 eq) is dissolved in 2 mL dry DCM and 0.5 mL TFA added. After 30 min the solvent is removed under reduced pressure and the TFA co-evaporated with DCM and toluene. The crude product is dissolved in 1.5 mL DMF and 30 µL TEA, then 5 (6)-TAMRA NHS ester (12 mg, 0.022 mmol, 1.0 eq) added. The flask is protected from light by an alumina foil wrapping. After stirring at r.t. overnight the solvent is removed under reduced pressure and the crude product purified by silica column chromatography (DCM + 15 MeOH). The regioisomers could be separated and three fractions were obtained. **14a** (pure regioisomer 1), **14b** (mixed fraction of both isomers), **14c** (regioisomer 2 still containing 12% isomer 1). All fractions were tested in an FP-Assay, with **14a** having the highest affinity.

TLC (DCM + 15 MeOH, 2/1): R_f (**14**) = 0.22.
Yield 14a, 10 mg (40%), **14b**, 7 mg (28%), **14c**, 8 mg (32%).
^{1}H-NMR (500 MHz, DMSO-*d*$_6$, 14a): δ 9.04 (t, J = 5.4 Hz, 1H), 8.47 – 8.43 (m, 1H), 8.26 – 8.19 (m, 1H), 8.17 (s, 1H), 7.55 (t, J = 10.1 Hz, 1H), 7.34 – 7.23 (m, 1H), 6.87 – 6.79 (m, 1H), 6.79 – 6.74 (m, 1H), 6.70 – 6.62 (m, 1H), 6.54 – 6.44 (m, 6H), 6.45 – 6.39 (m, 1H), 5.77 – 5.73 (m, 1H), 5.59 – 5.50 (m, 1H), 5.13 – 5.06 (m, 1H), 4.69 (d, J = 16.1 Hz, 1H), 4.53 (d, J = 15.5 Hz, 1H), 4.51 – 4.46 (m, 1H), 4.16 – 4.04 (m, 2H), 3.76 – 3.67 (m, 6H), 3.69 – 3.63 (m, 2H), 3.32 (s, 12H), 3.20 – 3.06 (m, 1H), 2.94 (s, 6H), 2.09 – 1.89 (m, 4H), 1.72 – 1.55 (m, 9H), 1.53 – 1.41 (m, 1H), 1.36 (s, 3H), 1.34 (s, 3H), 1.28 – 1.16 (m, 2H), 1.18 – 1.08 (m, 1H), 1.08 – 0.81 (m, 3H).
^{13}C-NMR (126 MHz, DMSO-*d*$_6$, 14a): δ 173.47, 172.88, 170.99, 167.64, 166.71, 160.09, 157.58, 152.11, 151.95, 148.63, 147.03, 143.17, 142.62, 135.82, 134.46, 133.27, 128.39, 126.87, 124.16, 123.20, 119.94, 112.21, 111.88, 108.97, 105.54, 102.14, 97.96, 88.51, 75.12, 65.91, 61.76, 60.96, 56.16, 55.47, 55.36, 54.88, 52.66, 51.68, 43.52, 40.71, 37.58, 33.69, 31.25, 30.62, 30.18, 29.91, 28.64, 27.08, 26.92, 25.78, 25.65, 25.53, 24.99, 24.57, 24.49, 23.16, 23.08.

HRMS (ESI): 14a calculated = 1135.53866 $[M]^+$, found = 1135.54008 $[M]^+$, err [ppm] = 1.25.

HRMS (ESI): 14b calculated = 1135.53866 $[M]^+$, found = 1135.54043 $[M]^+$, err [ppm] = 1.56.

HRMS (ESI): 14c calculated = 1135.53866 $[M]^+$, found = 1135.53983 $[M]^+$, err [ppm] = 1.03.

RP-HPLC (50 – 100% B, 1.5 mL/min, 20 min, 254 nm): t_R (**14a**) = 8.37 min (99% Purity)

RP-HPLC (50 – 100% B, 1.5 mL/min, 20 min, 254 nm): t_R (**14b**) = 7.85 min, 8.39 (34%, 66%)

RP-HPLC (50 – 100% B, 1.5 mL/min, 20 min, 254 nm): t_R (**14c**) = 7.75, 8.40 min (87%, 12%)

10. Analytical data (HPLC, NMR)

Figure S7. ^{1}H-NMR of compound **8aZ**.

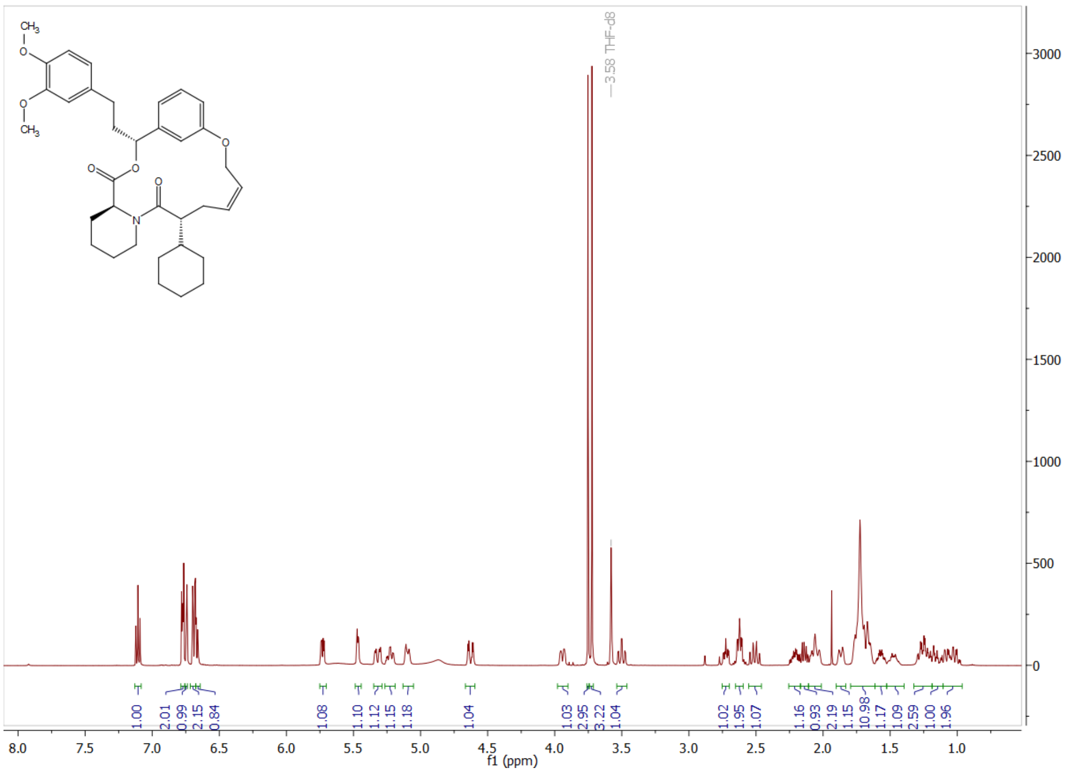

Figure S8. ^{13}C-NMR of compound **8aZ**.

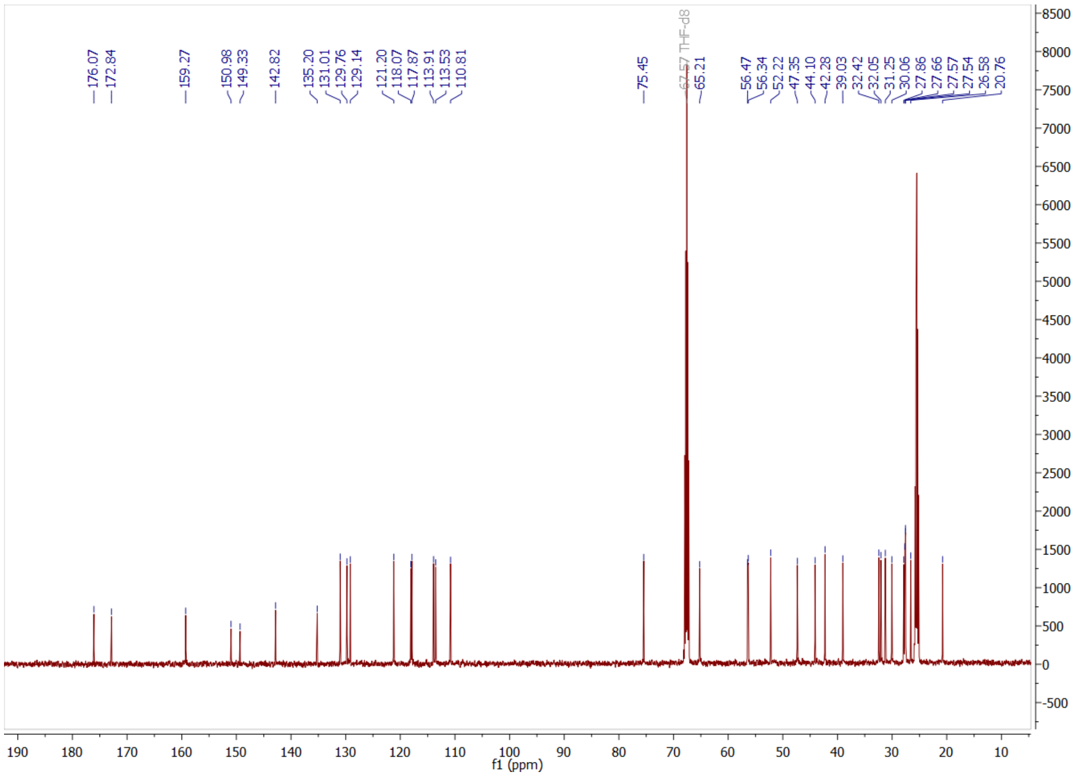

Figure S9. HPLC of compound 8aZ.

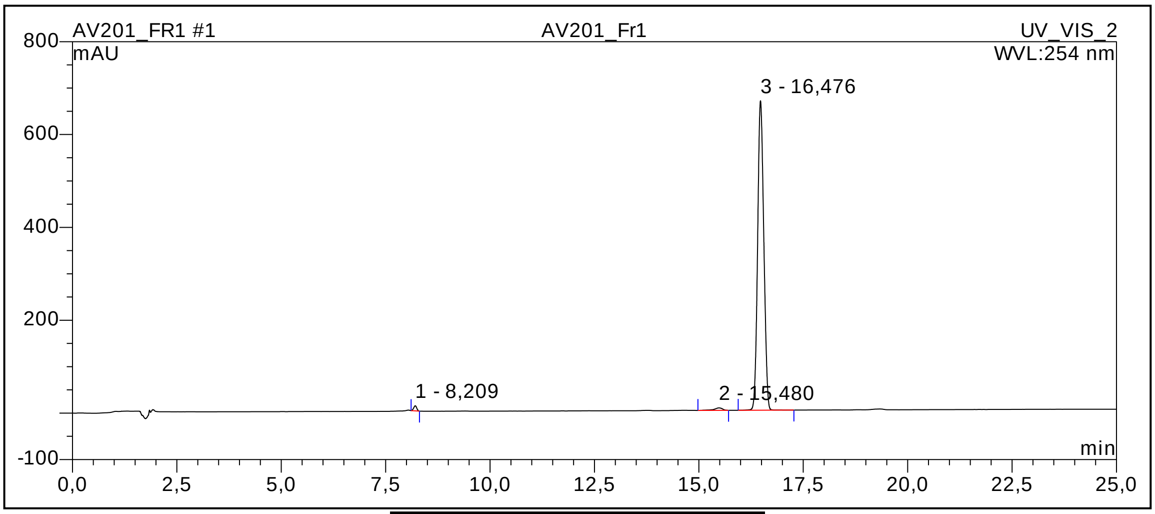

No.	Ret.Time min	Rel.Area %
1	8,21	0,79
2	15,48	1,05
3	16,48	98,15
Total		100

Figure S10. ^{1}H-NMR of compound **8aE**.

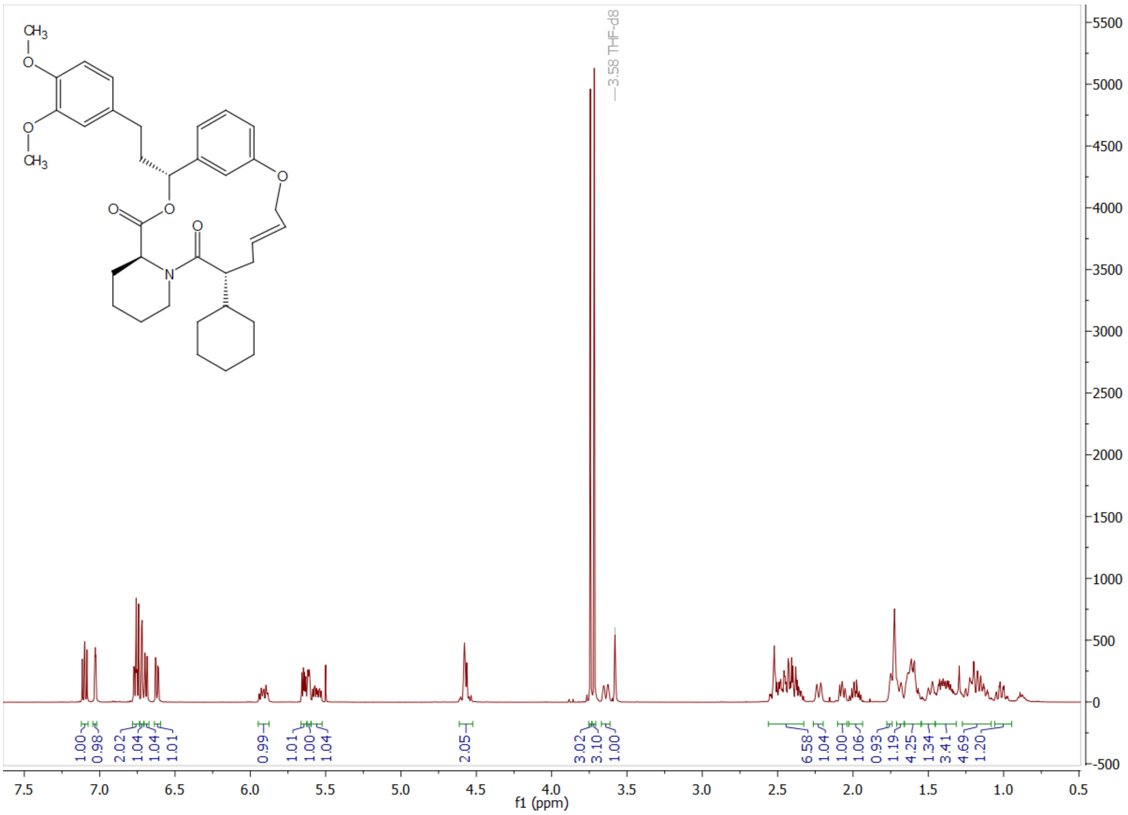

Figure S11. ^{13}C-NMR of compound **8aE**.

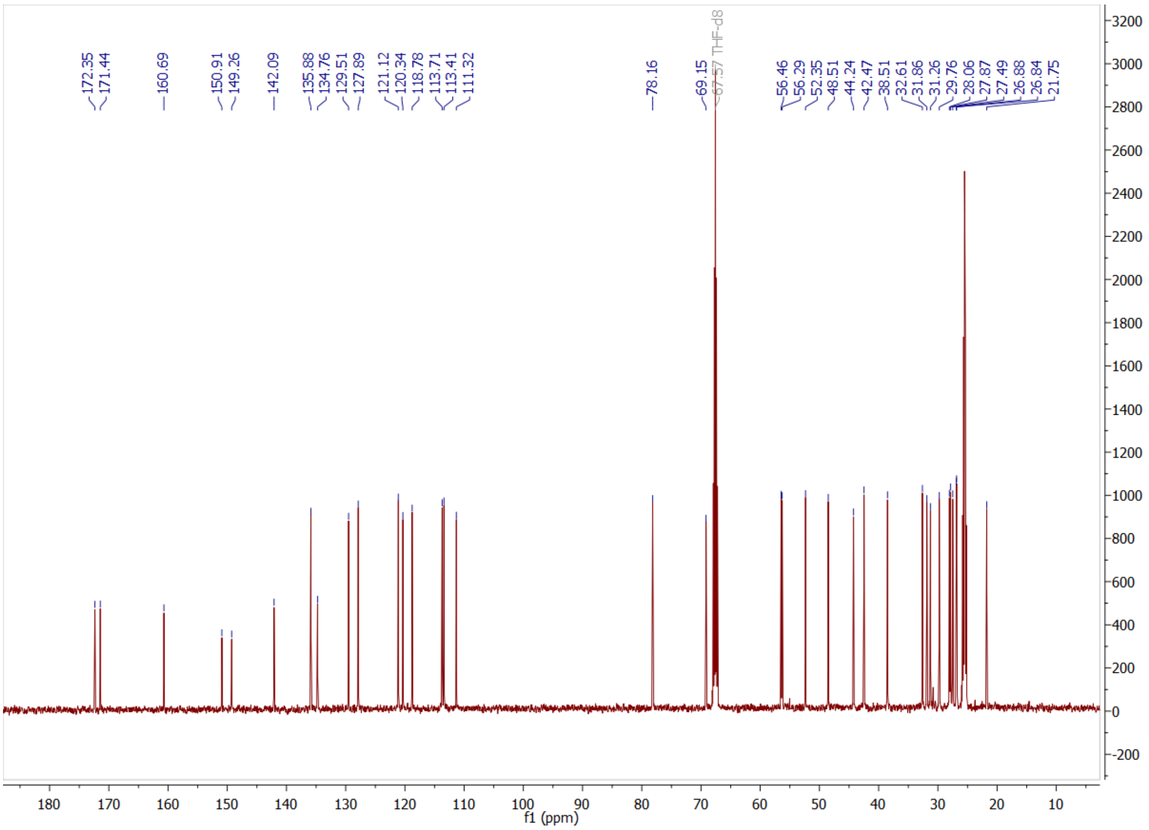

Figure S12. HPLC of compound **8aE**.

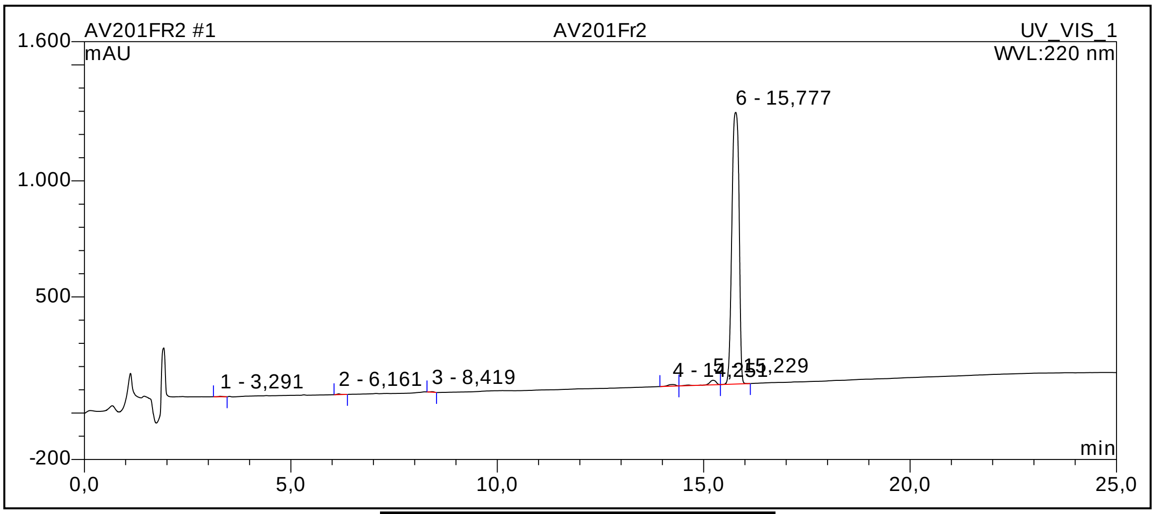

No.	Ret.Time min	Rel.Area %
1	3,29	0,09
2	6,16	0,11
3	8,42	0,11
4	14,25	0,62
5	15,23	1,63
6	15,78	97,45
Total		100

Figure S13. ^{1}H-NMR of compound **8b**.

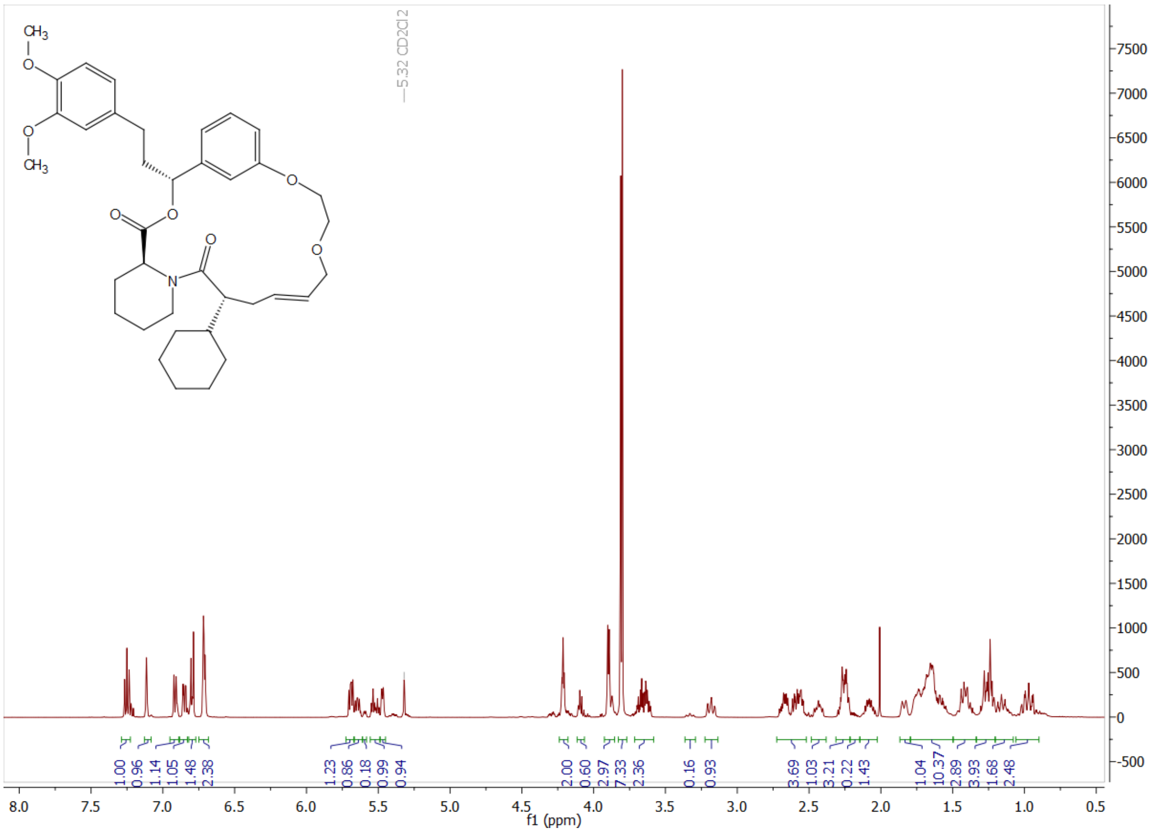

Figure S14. ^{13}C-NMR of compound **8b**.

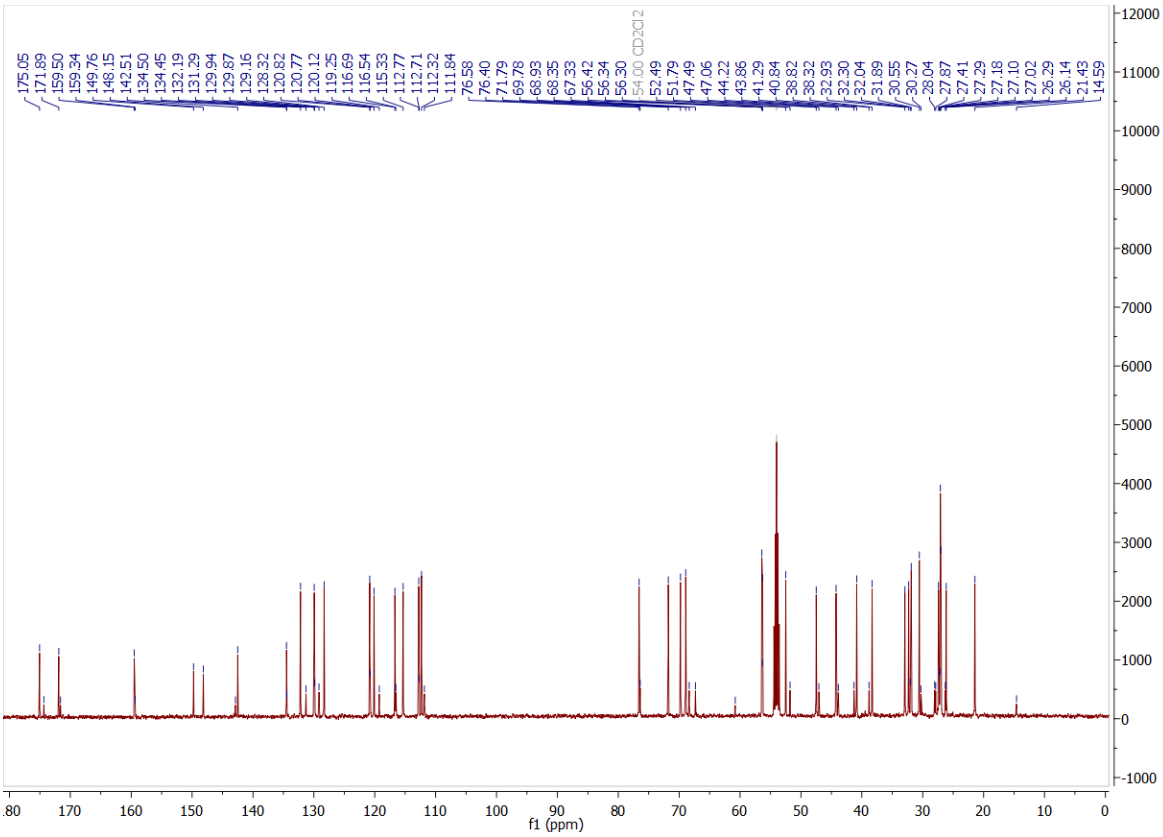

Figure S15. HPLC of compound **8b**.

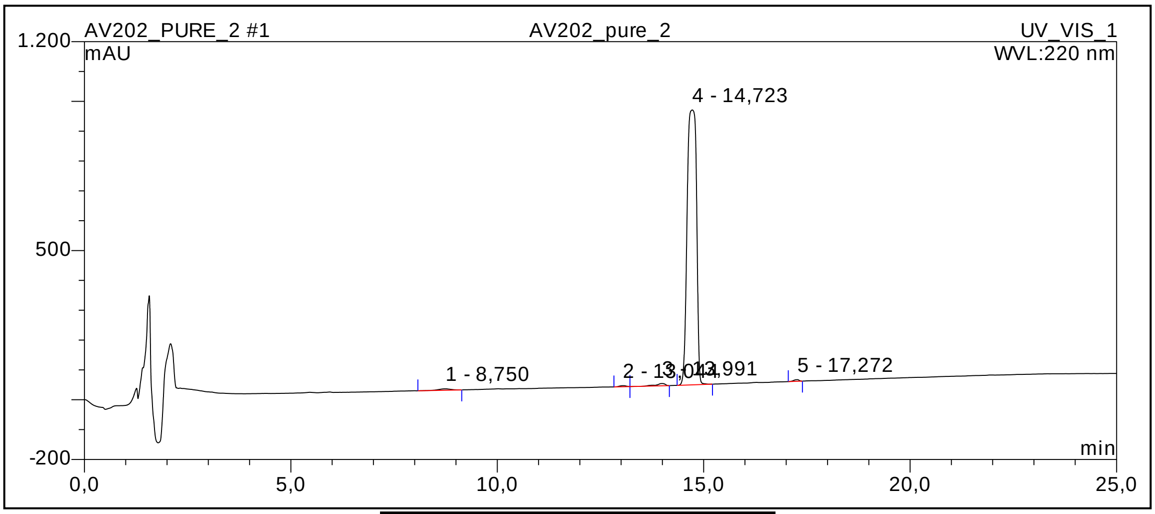

No.	Ret.Time min	Rel.Area %
1	8,75	0,71
2	13,04	0,28
3	13,99	0,79
4	14,72	97,88
5	17,27	0,34
Total		100

Figure S16. ^{1}H-NMR of compound **9a**.

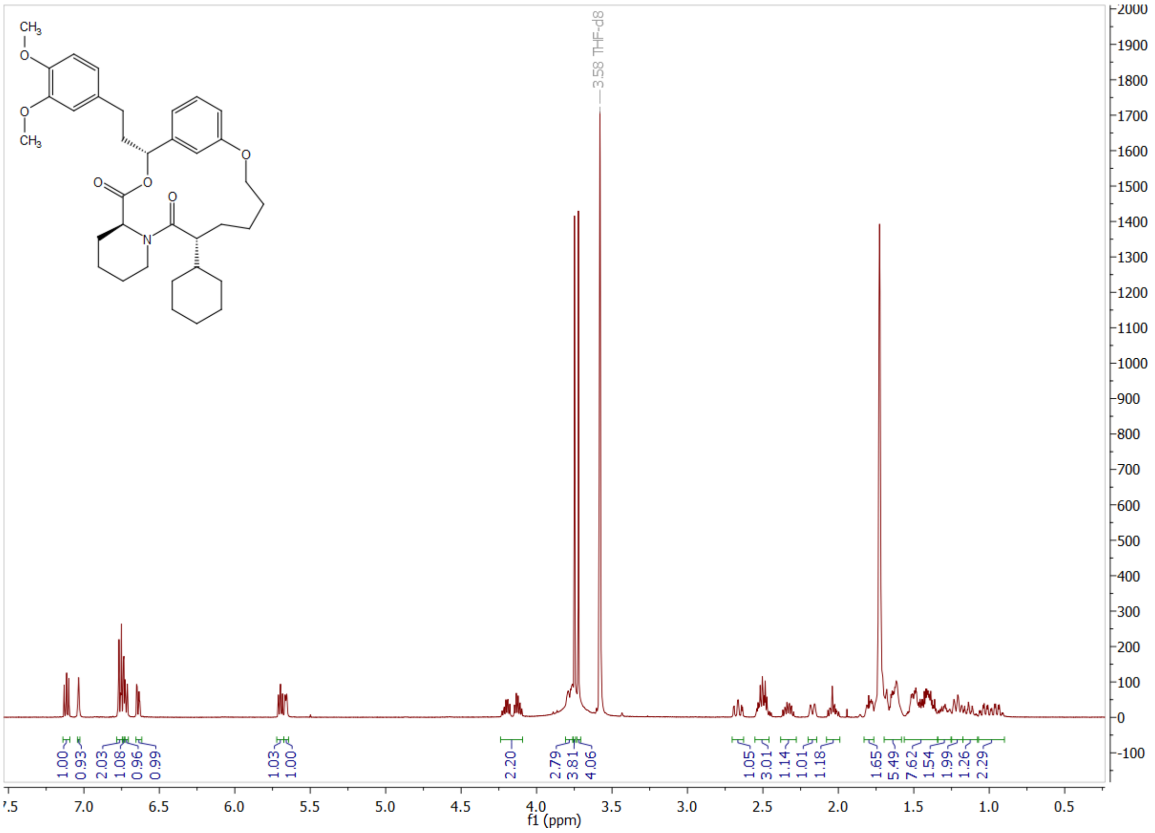

Figure S17. ^{13}C-NMR of compound **9a**.

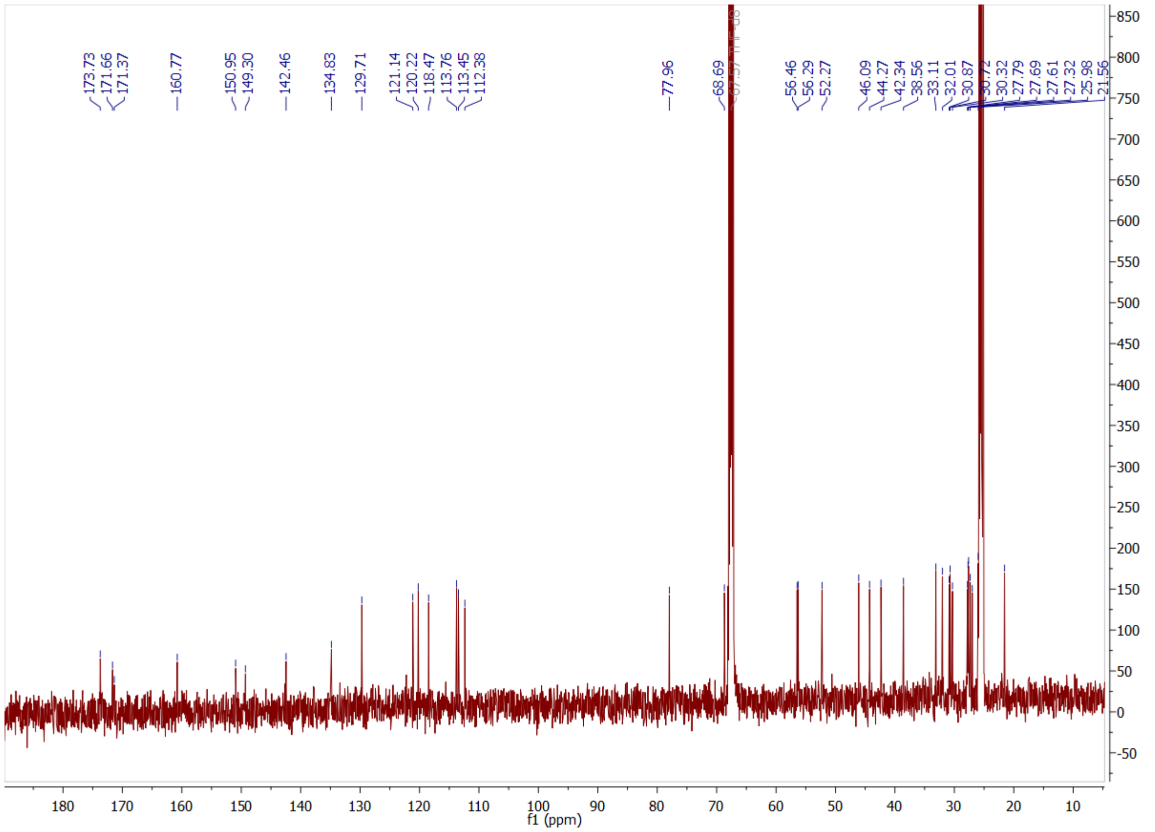

Figure S18. HPLC of compound **9a**.

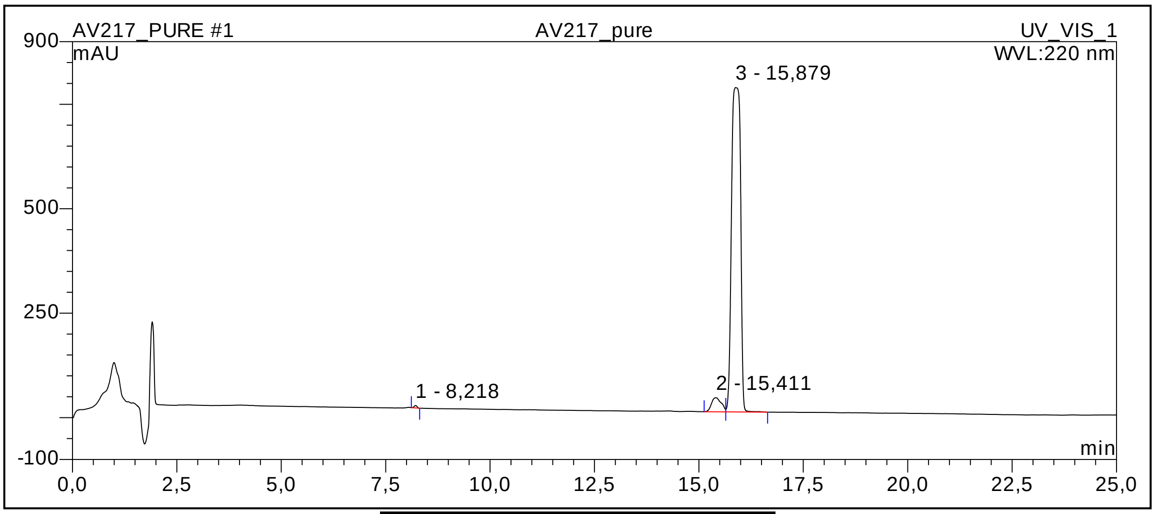

No.	Ret.Time min	Rel.Area %
1	8,22	0,23
2	15,41	4,50
3	15,88	95,27
Total		100

Figure S19. ^{1}H-NMR of compound **9b**.

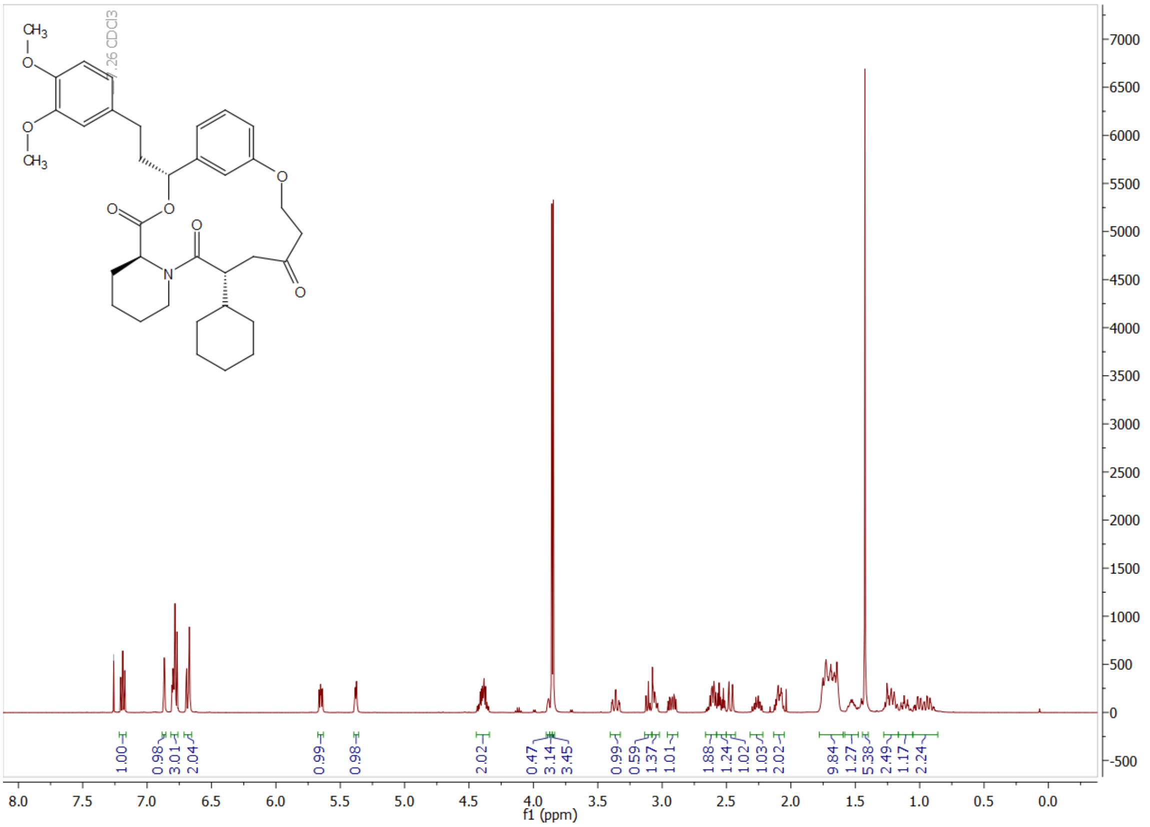

Figure S20. ^{13}C-NMR of compound **9b**.

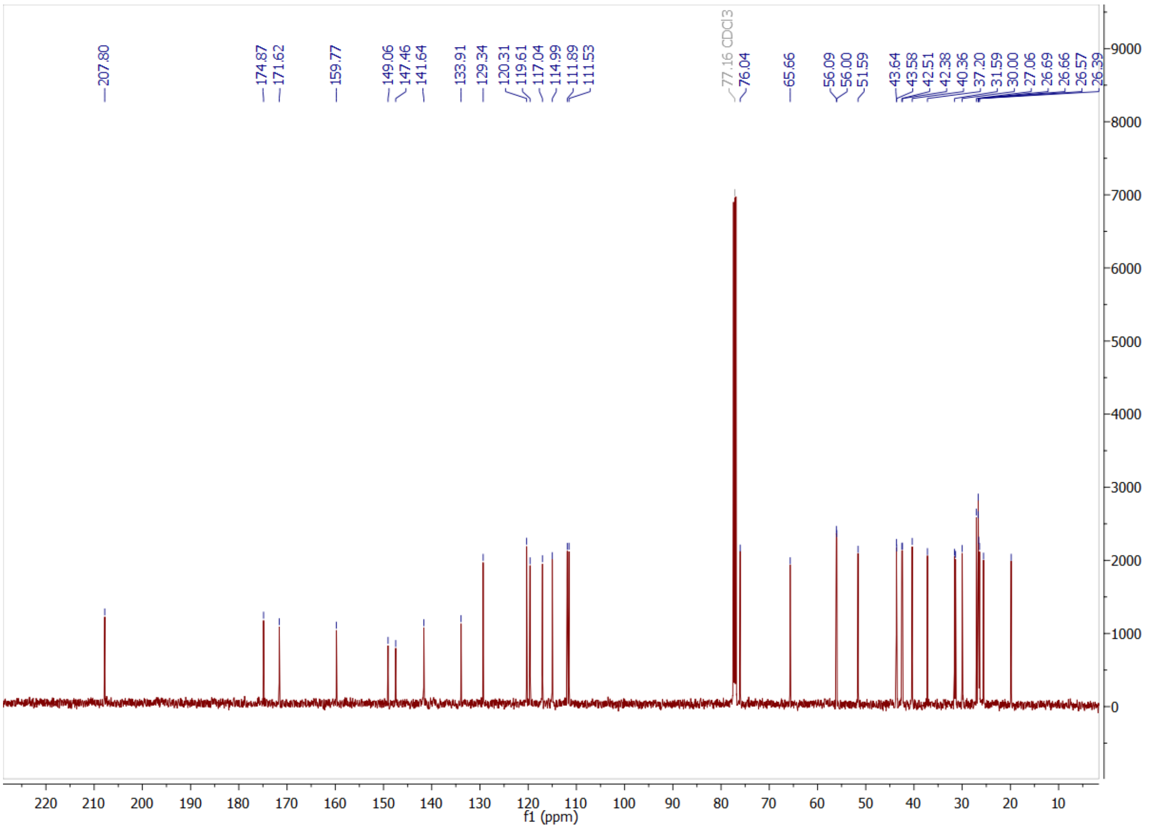

Figure S21. HPLC of compound **9b**.

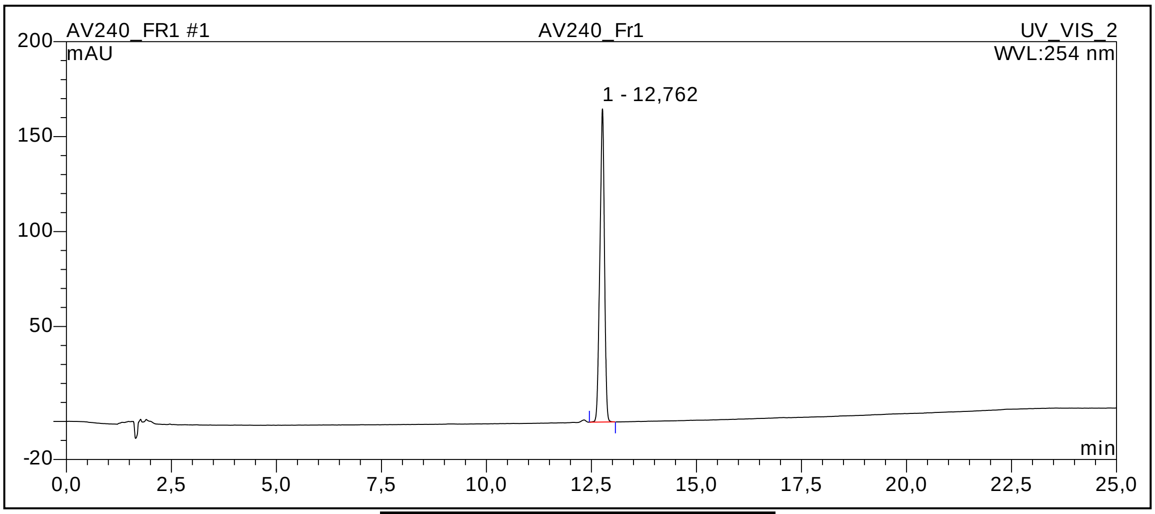

No.	Ret.Time min	Rel.Area %
1	12,76	100,00
Total		100

Figure S22. ^{1}H-NMR of compound **9c**.

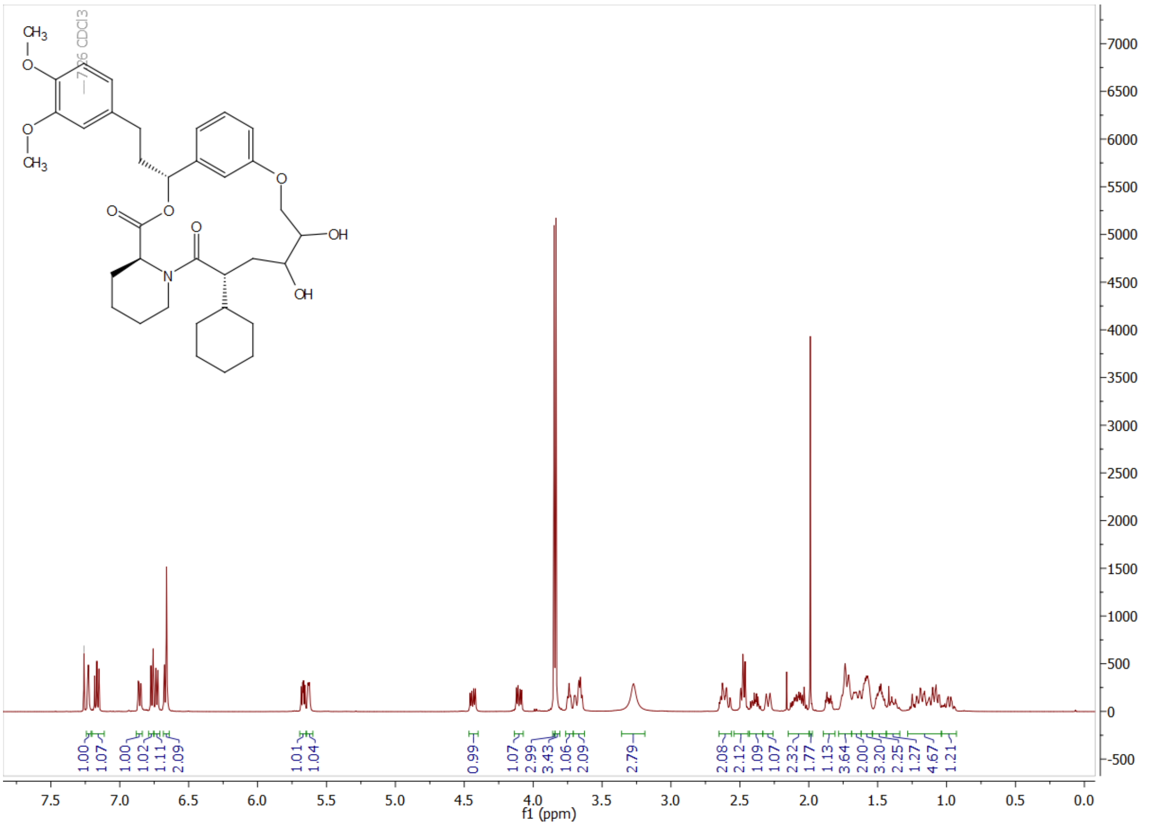

Figure S23. ^{13}C-NMR of compound **9c**.

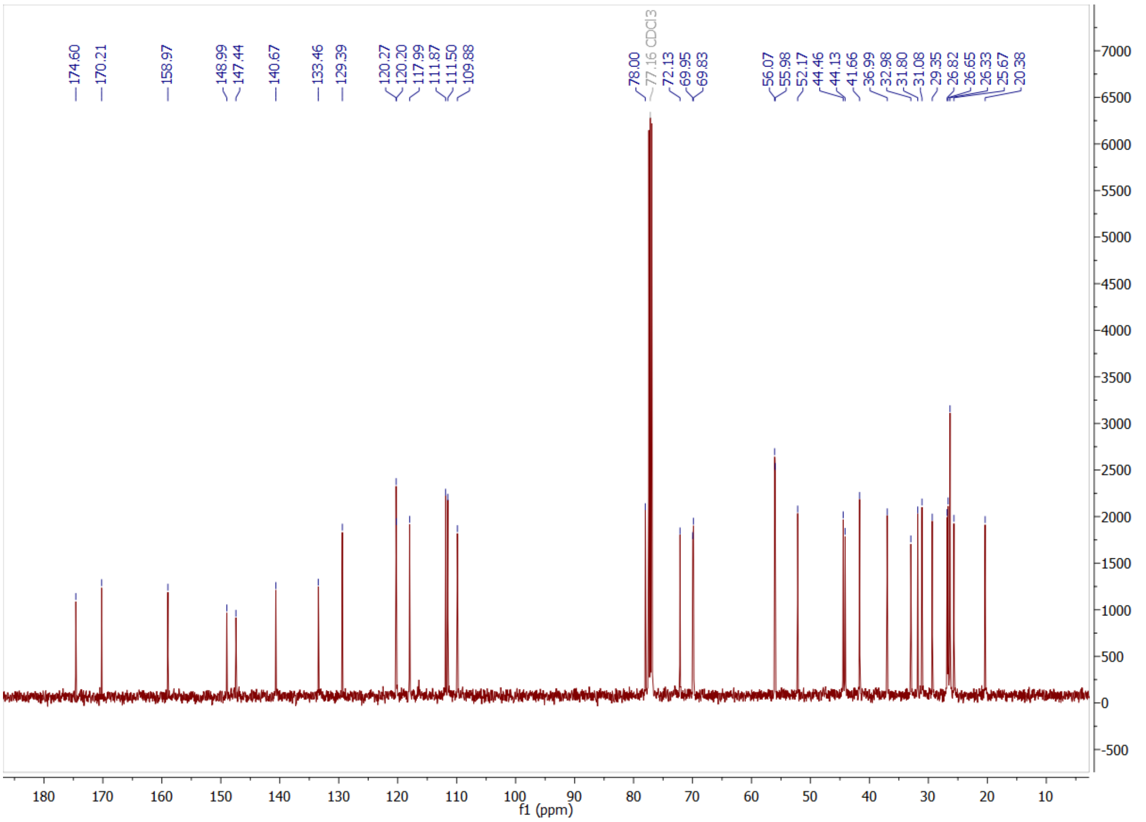

Figure S24. HPLC of compound **9c**.

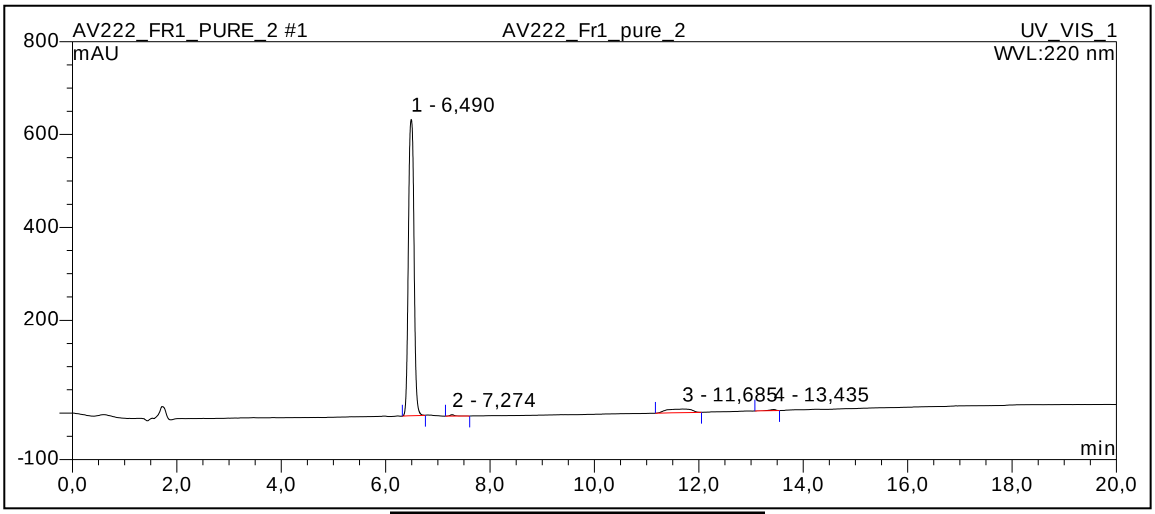

No.	Ret.Time min	Rel.Area %
1	6,49	93,91
2	7,27	0,41
3	11,69	5,25
4	13,44	0,43
Total		100

Figure S25. ^{1}H-NMR of compound **9d**.

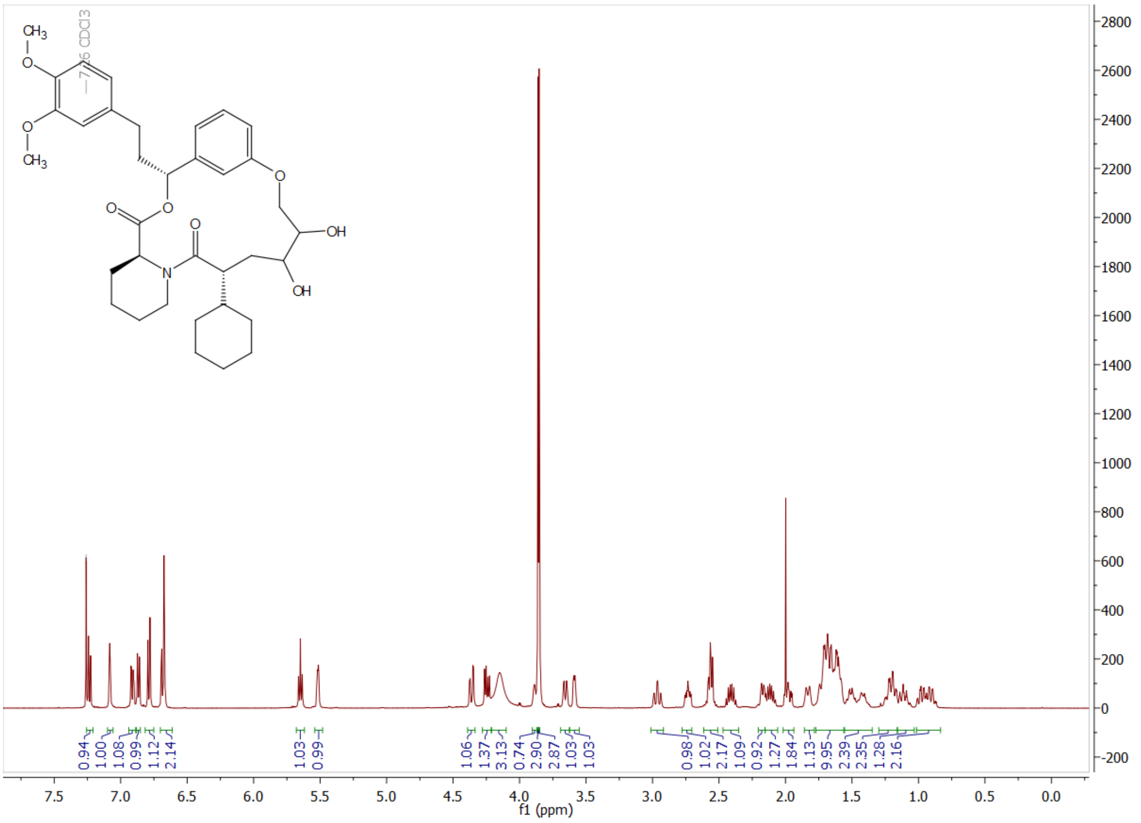

Figure S26. ^{13}C-NMR of compound **9d**.

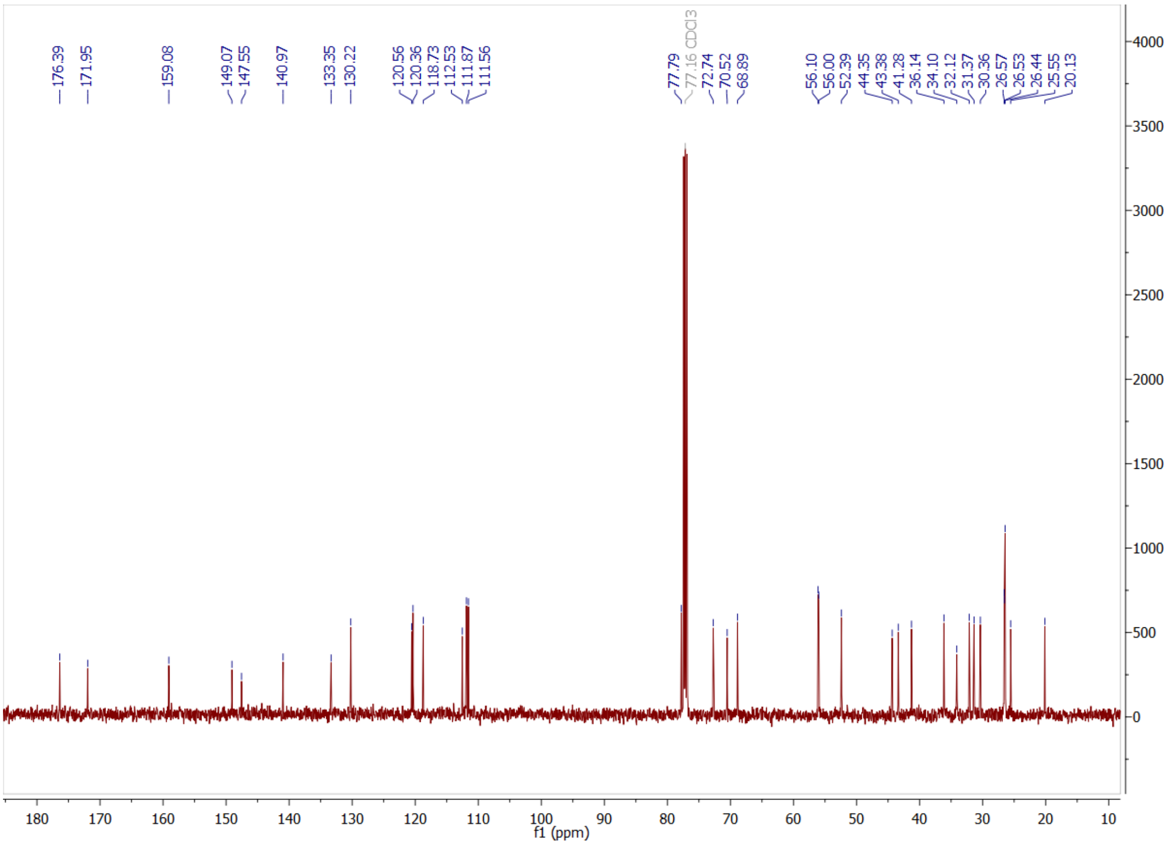

Figure S27. HPLC of compound **9d**.

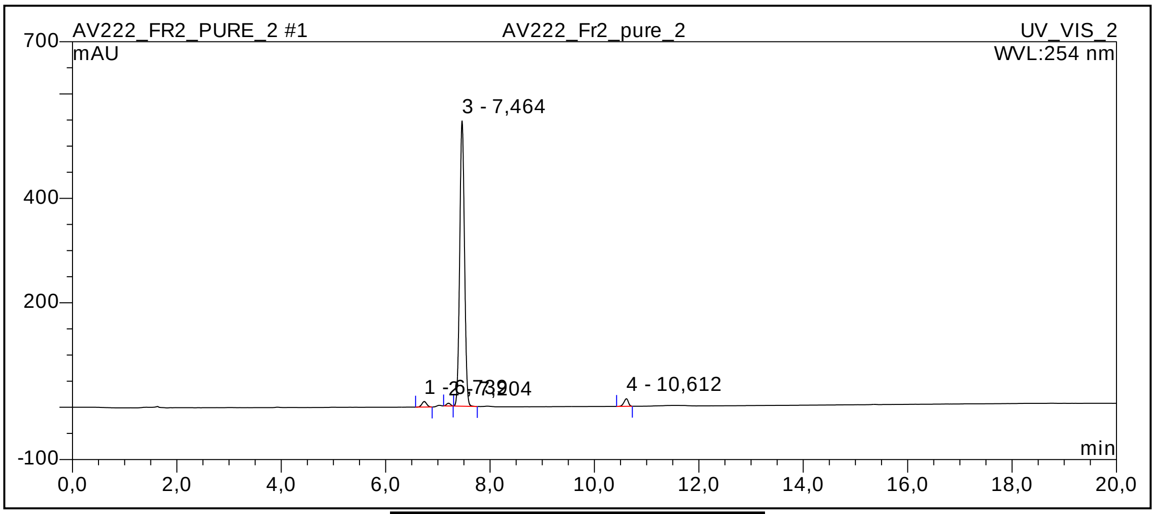

No.	Ret.Time min	Rel.Area %
1	6,74	1,87
2	7,20	0,76
3	7,46	95,03
4	10,61	2,34
Total		100

Figure S28. ^{1}H-NMR of compound **9e**.

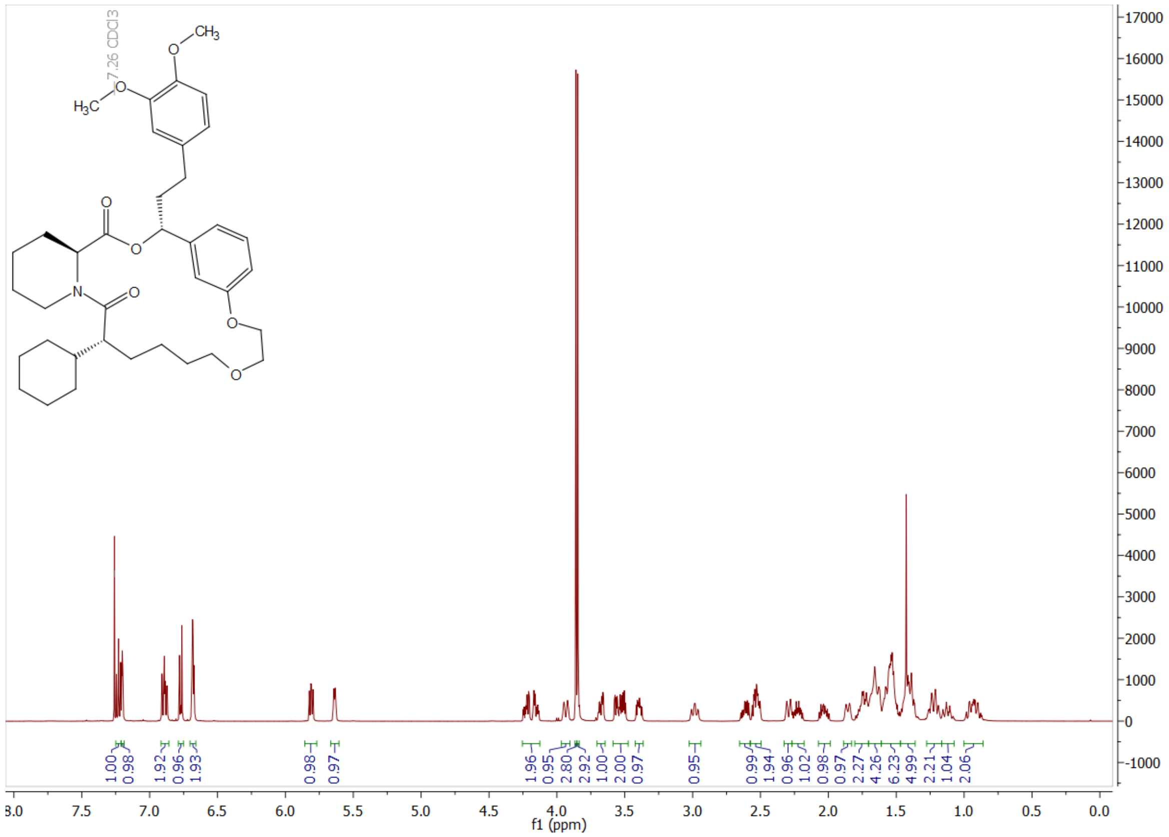

Figure S29. ^{13}C-NMR of compound **9e**.

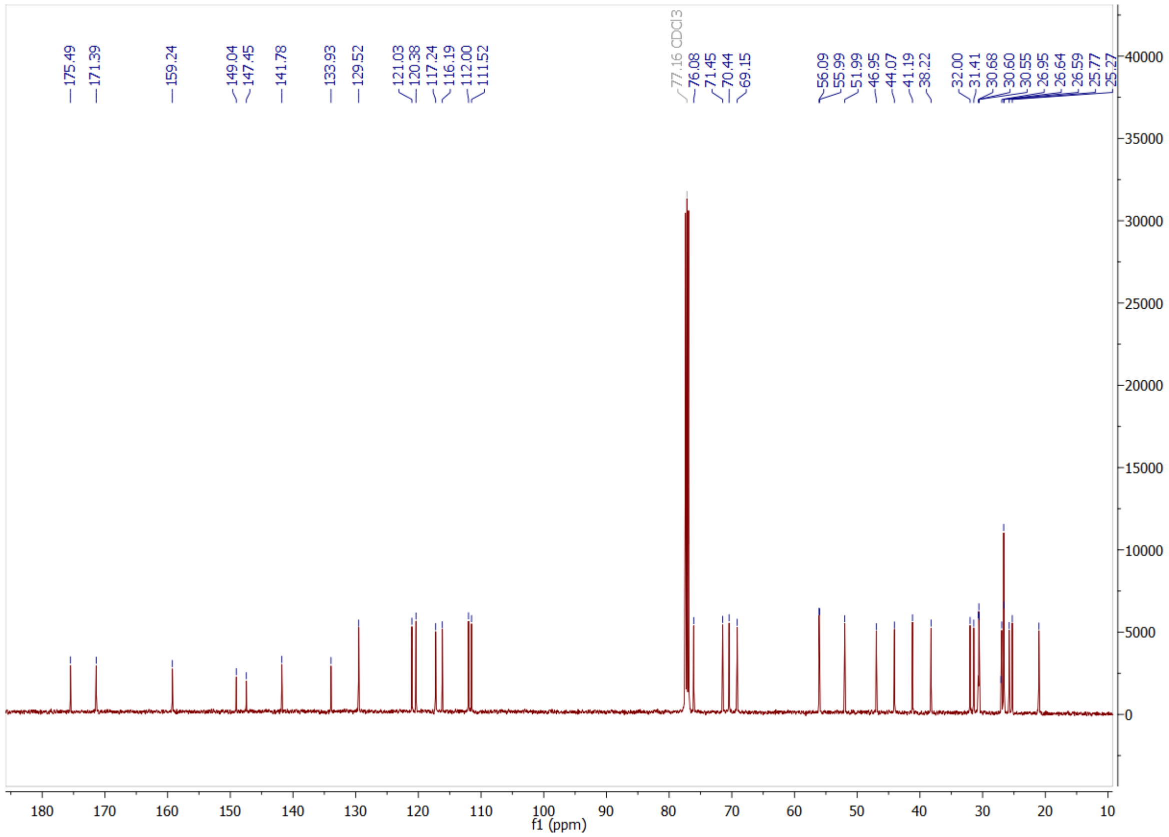

Figure S30. HPLC of compound **9e**.

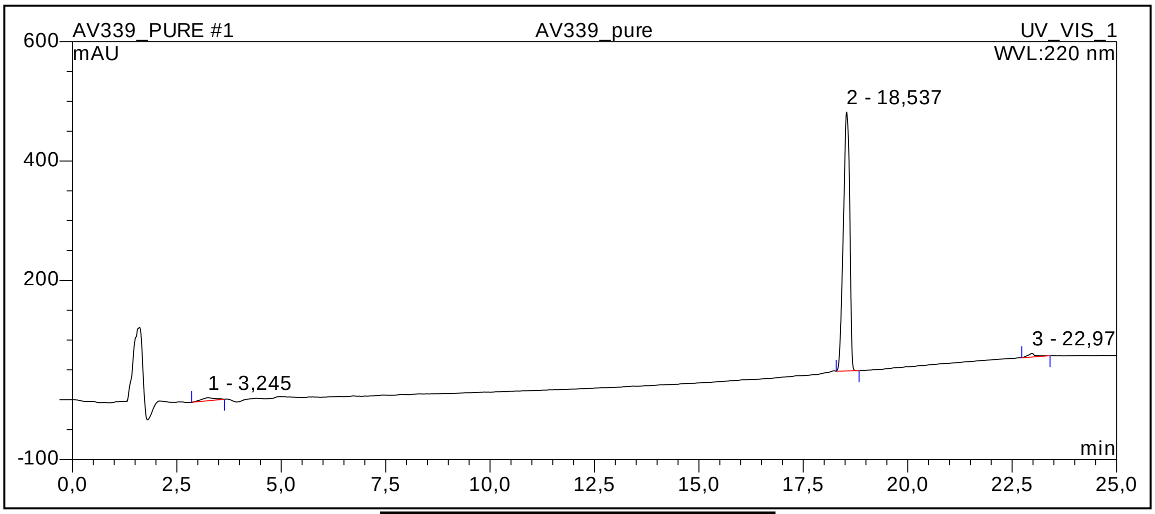

No.	Ret.Time min	Rel.Area %
1	3,25	2,49
2	18,54	95,94
3	22,98	1,57
Total		100

Figure S31. ^{1}H-NMR of compound **9f**.

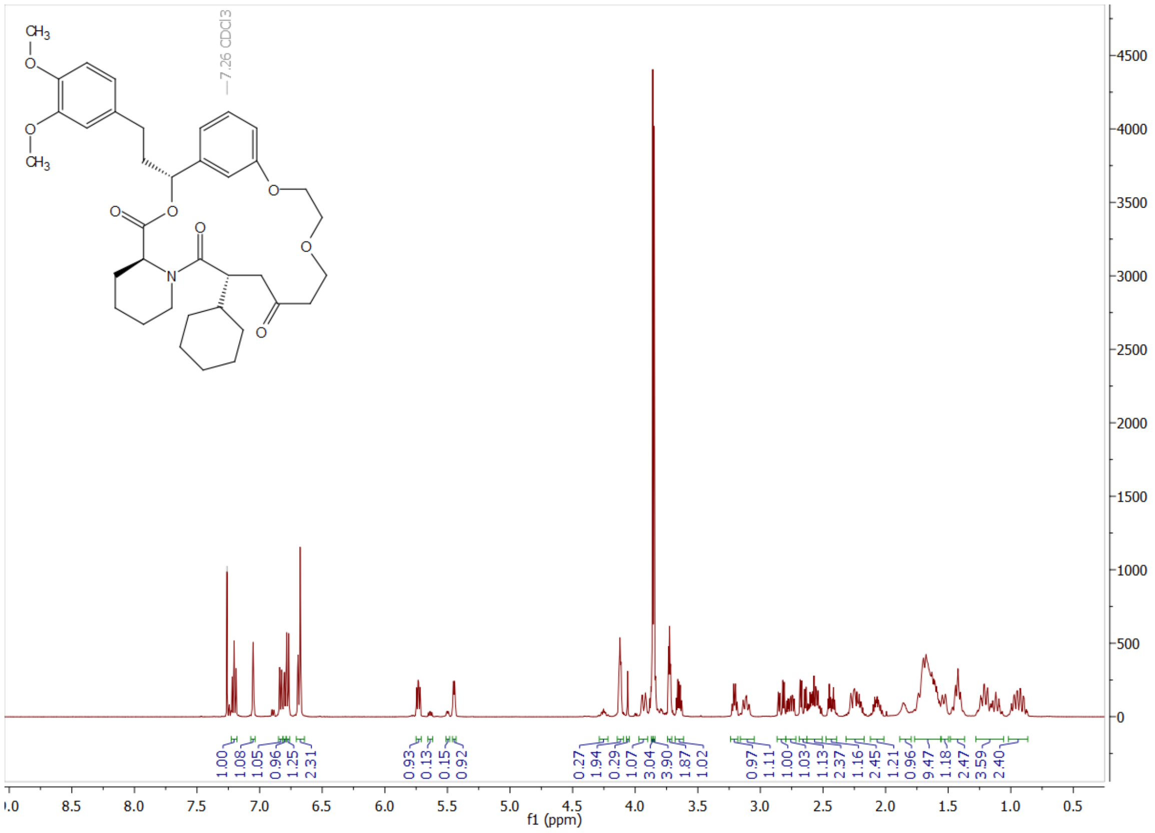

Figure S32. ^{13}C-NMR of compound **9f**.

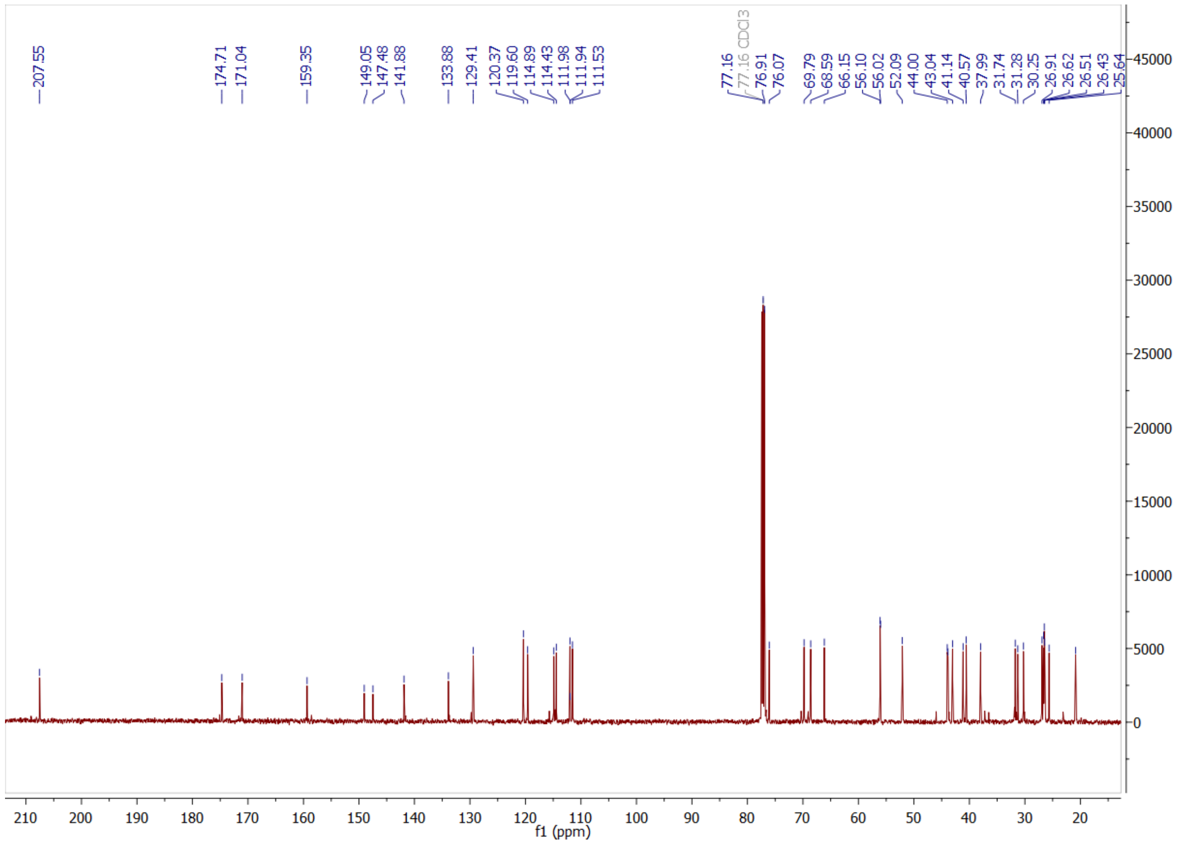

Figure S33. HPLC of compound **9f**.

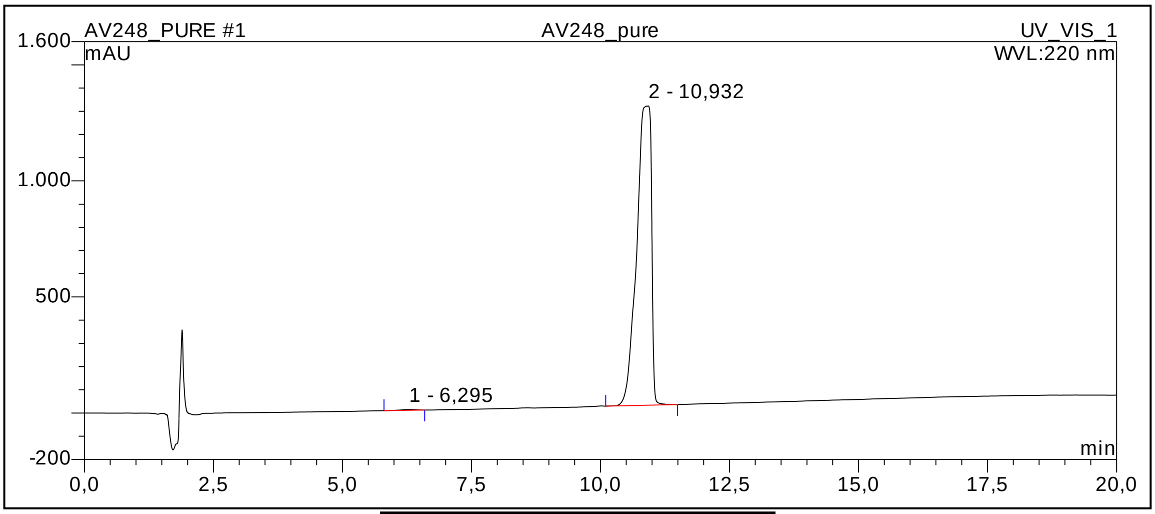

No.	Ret.Time min	Rel.Area %
1	6,29	0,26
2	10,93	99,74
Total		100

Figure S34. ^{1}H-NMR of compound **9g**.

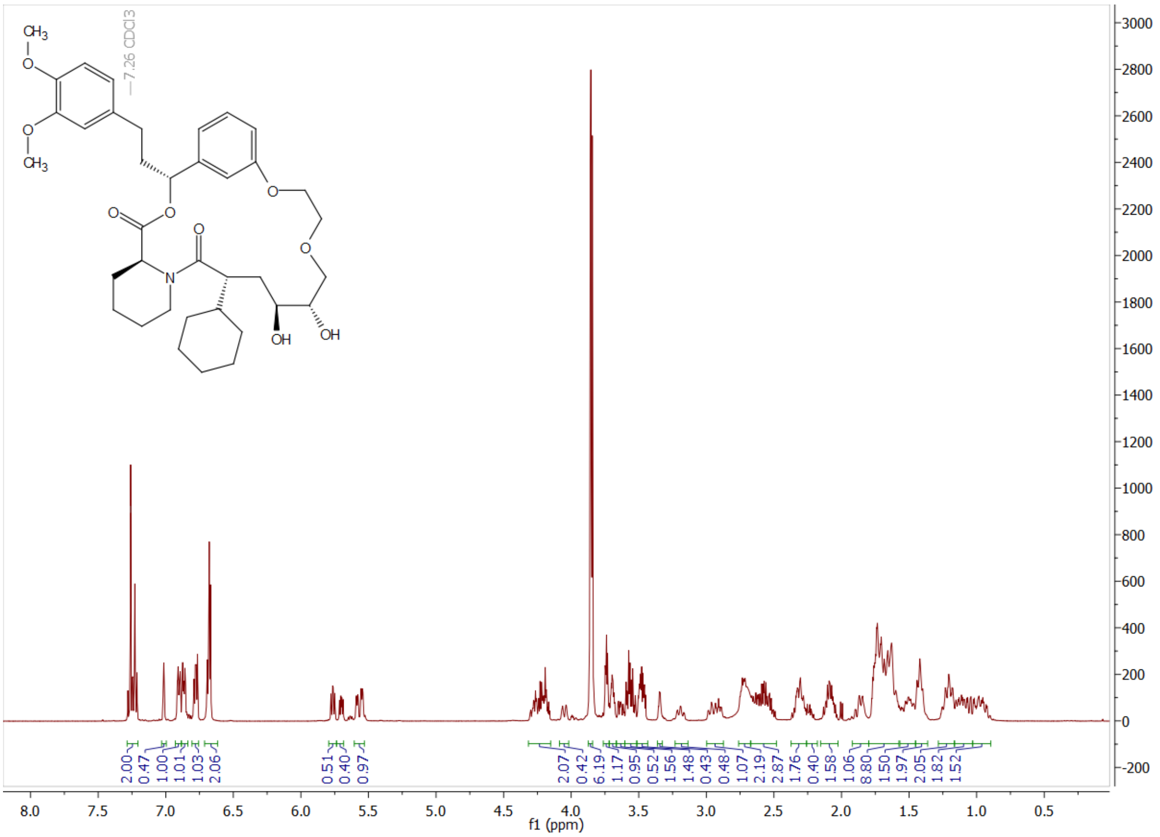

Figure S35. ^{13}C-NMR of compound **9g**.

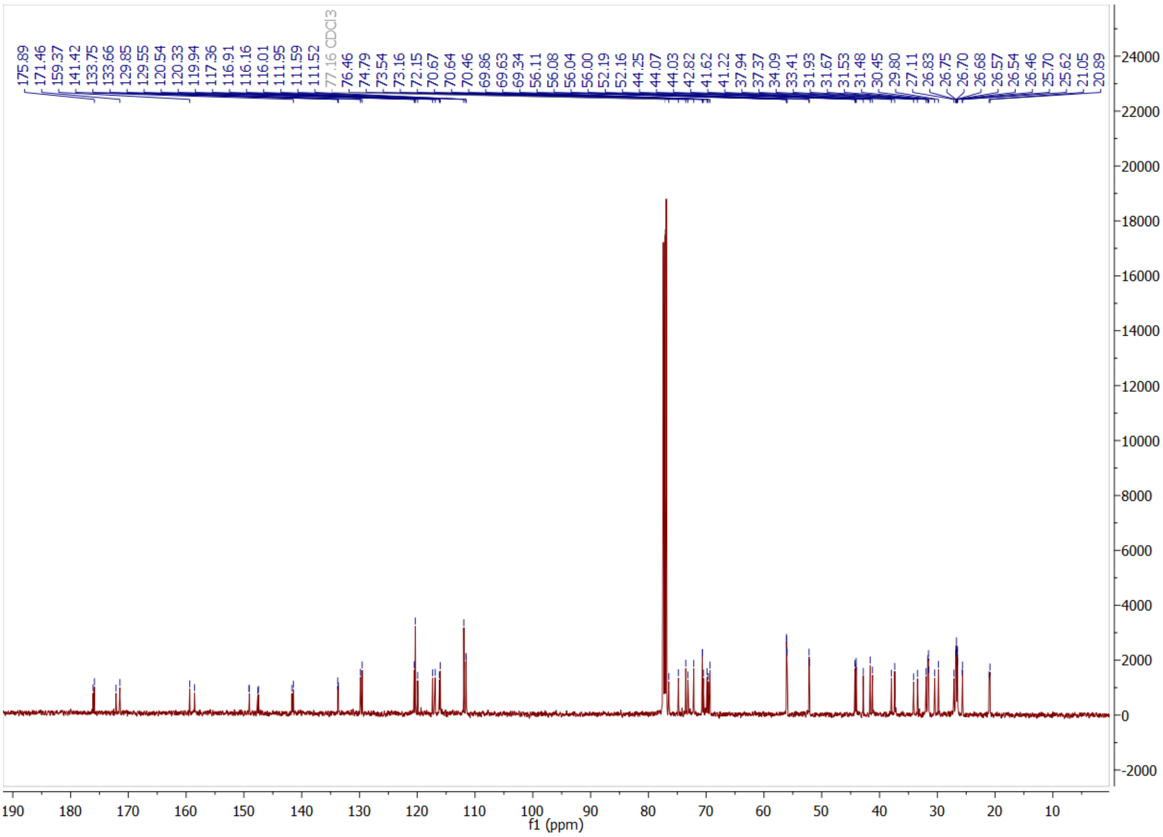

SUPPORTING INFORMATION

Figure S36. HPLC of compound **9g**.

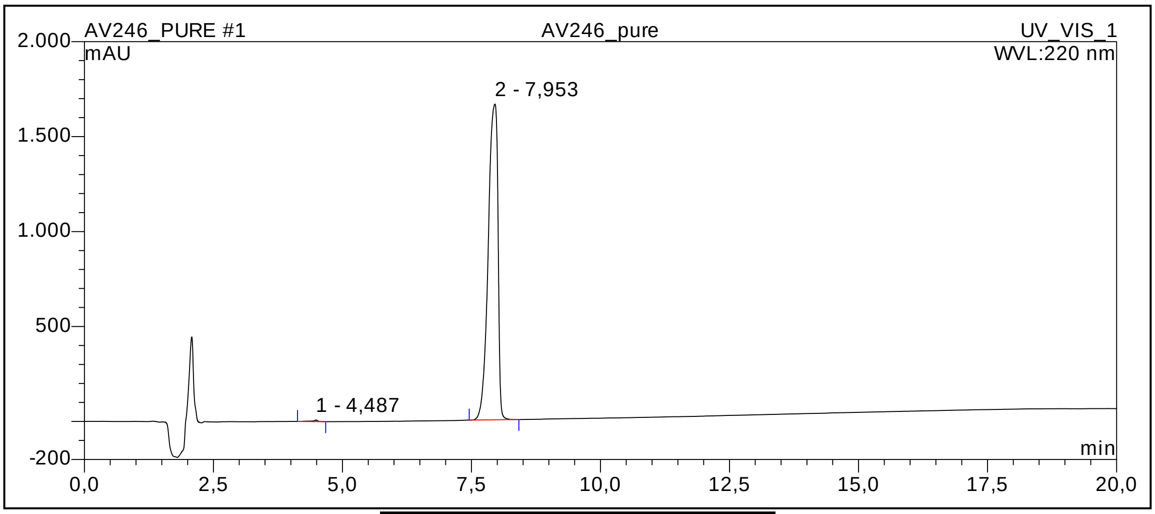

No.	Ret.Time min	Rel.Area %
1	4,49	0,30
2	7,95	99,70
Total		100

Figure S37. ^{1}H-NMR of compound **13a**.

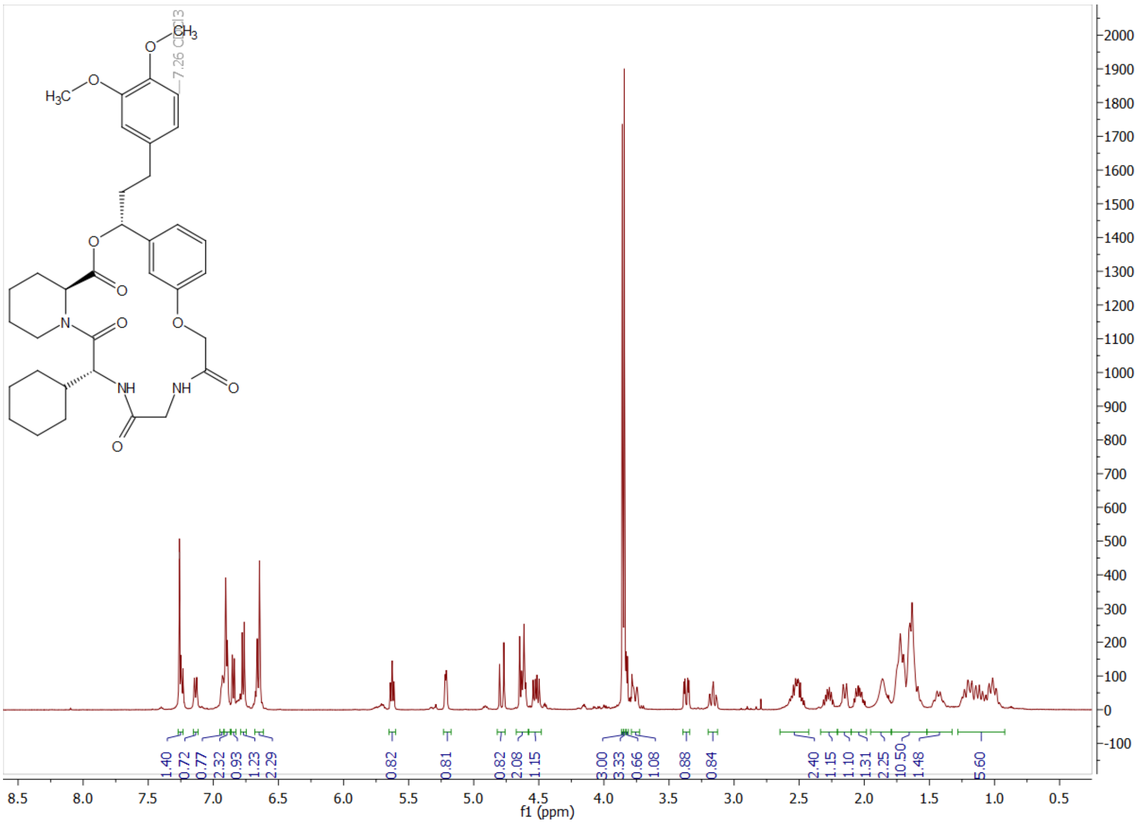

Figure S38. ^{13}C-NMR of compound **13a**.

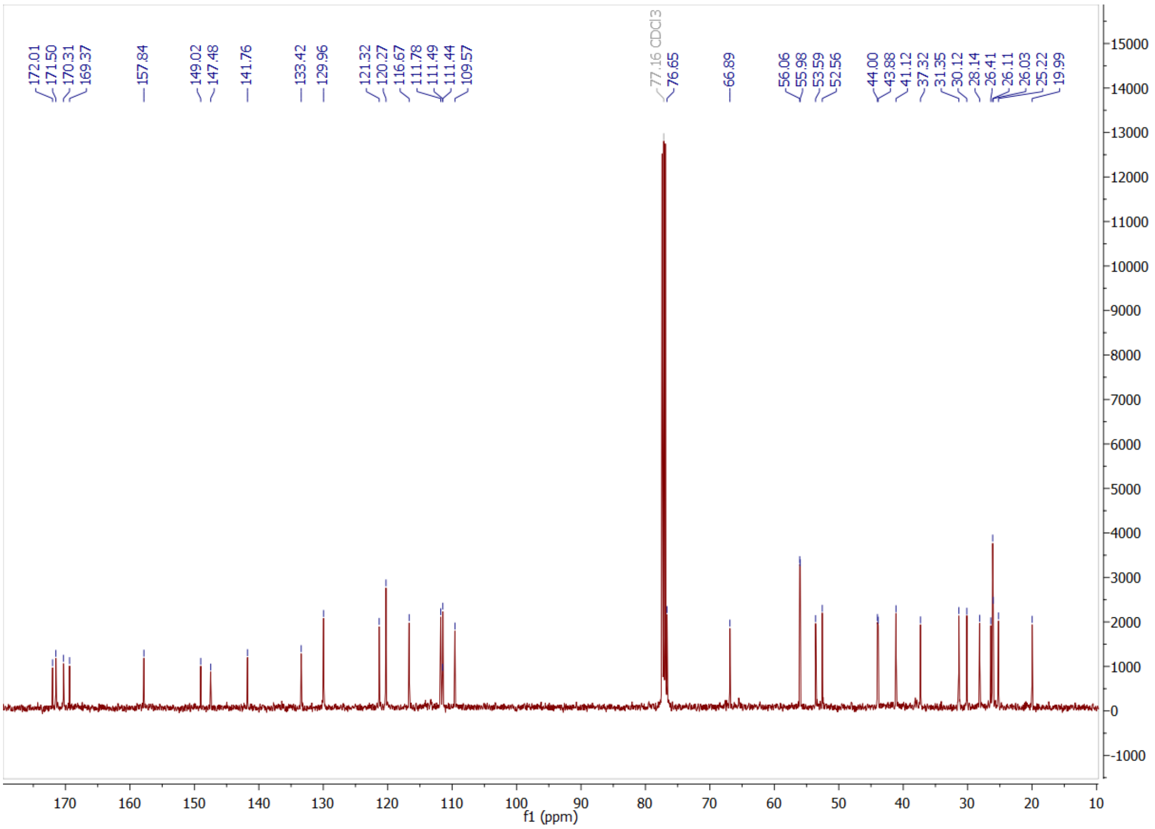

Figure S39. HPLC of compound **13a**.

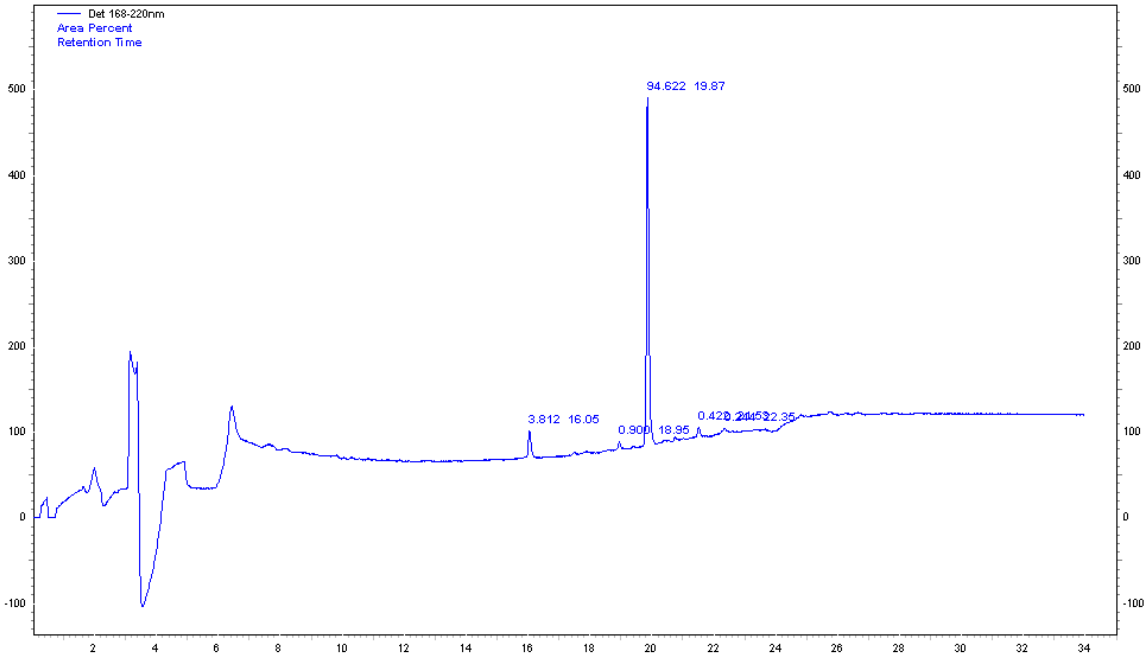

Figure S40. ^{1}H-NMR of compound **13b**.

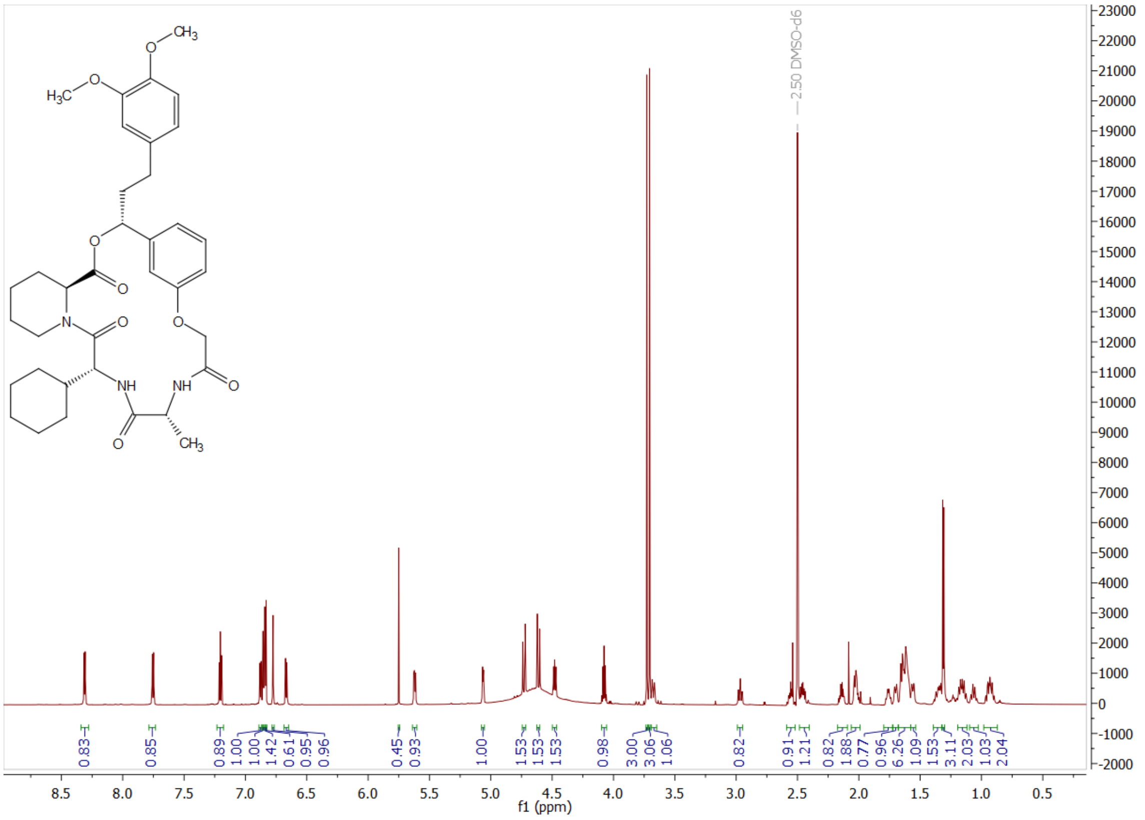

Figure S41. ^{13}C-NMR of compound **13b**.

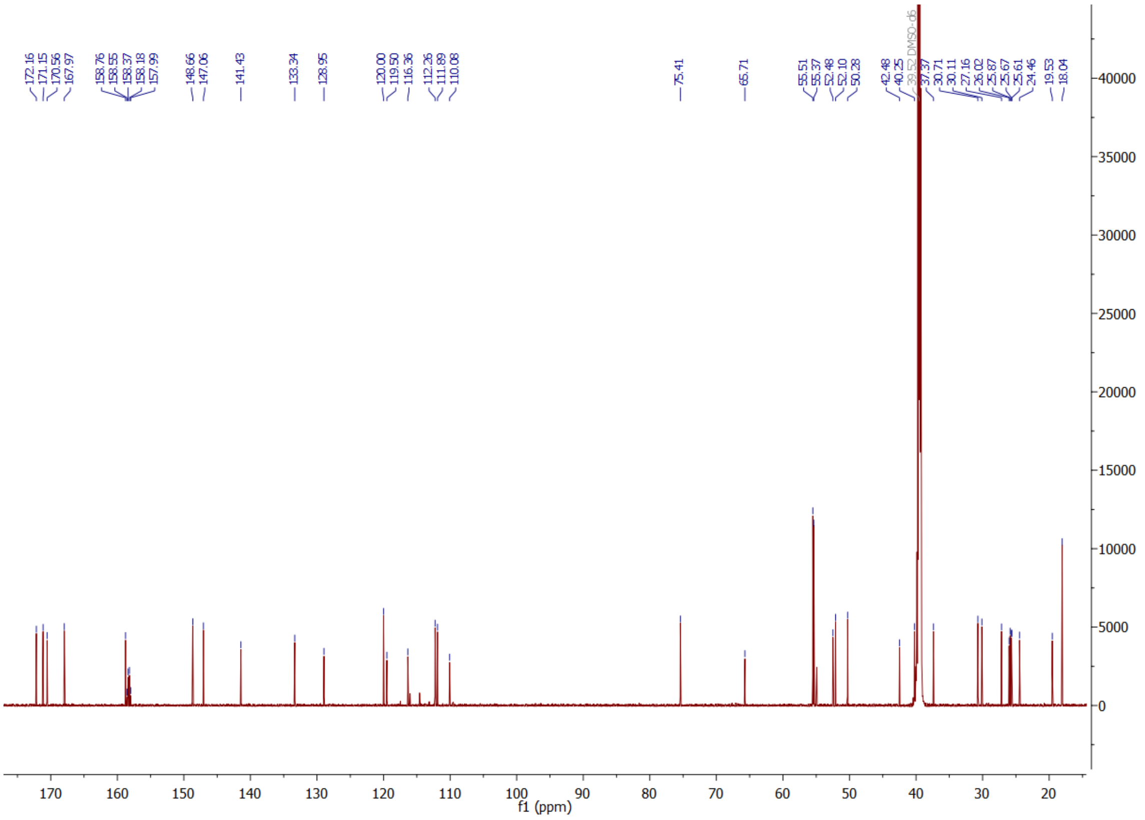

Figure S42. HPLC of compound **13b**.

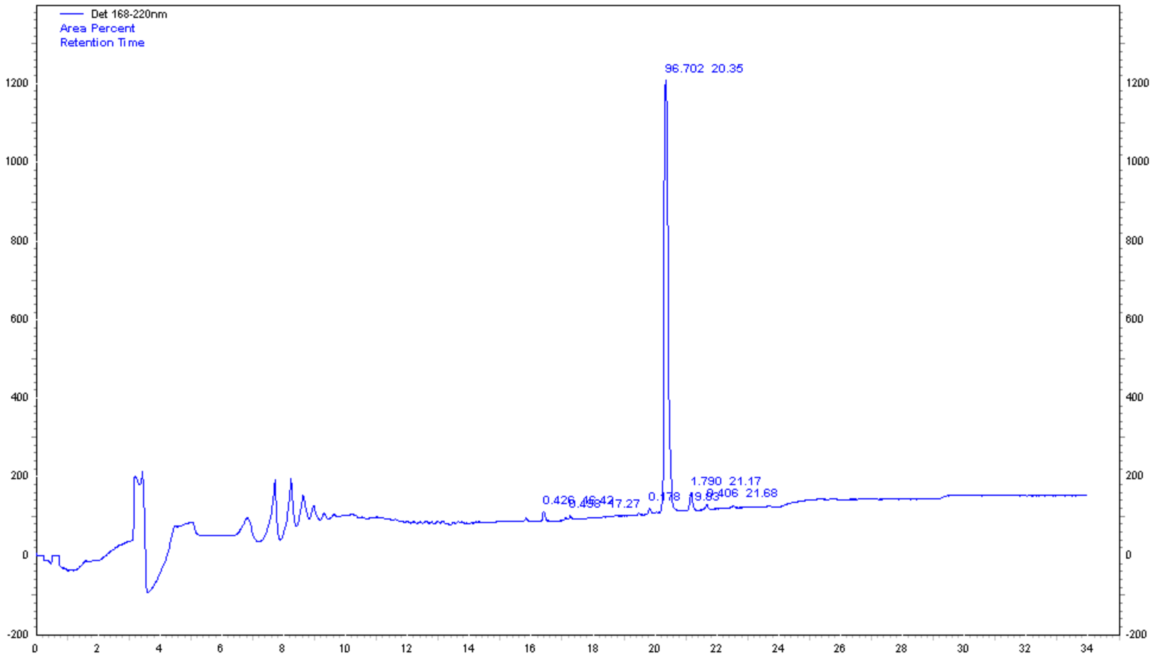

Figure S43. ^{1}H-NMR of compound **13c**.

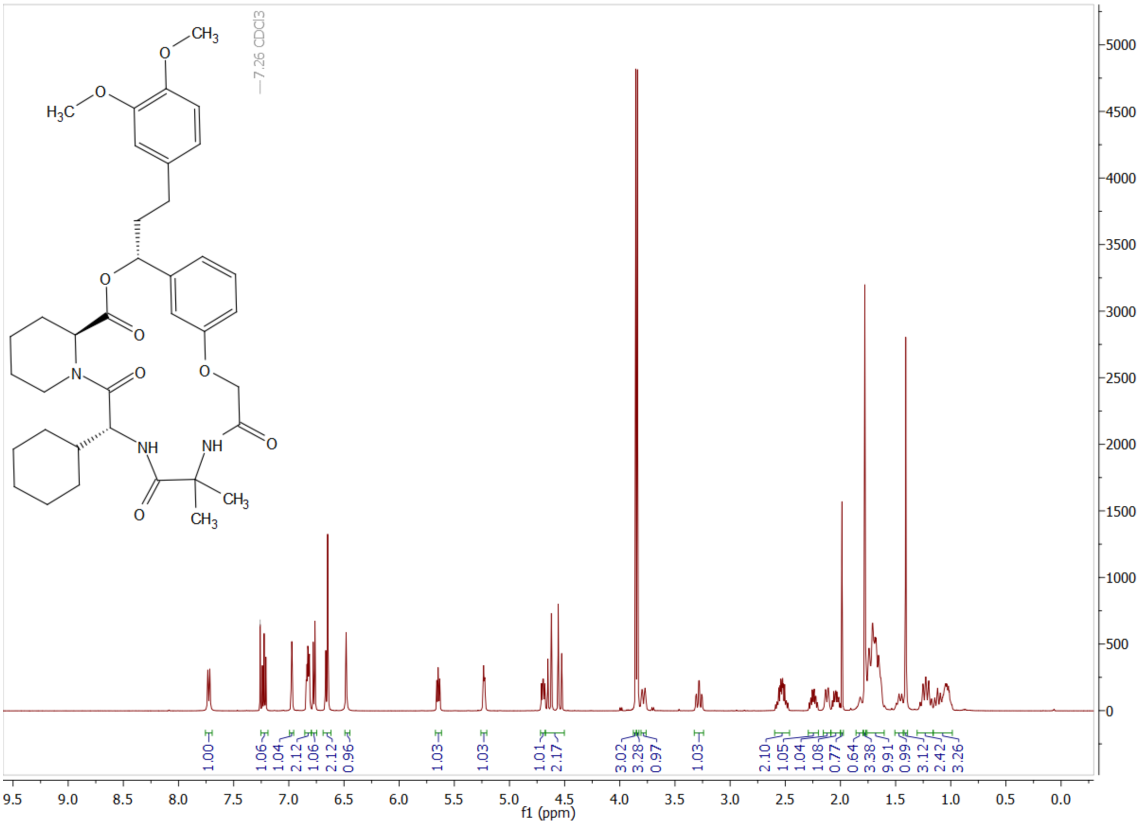

Figure S44. ^{13}C-NMR of compound **13c**.

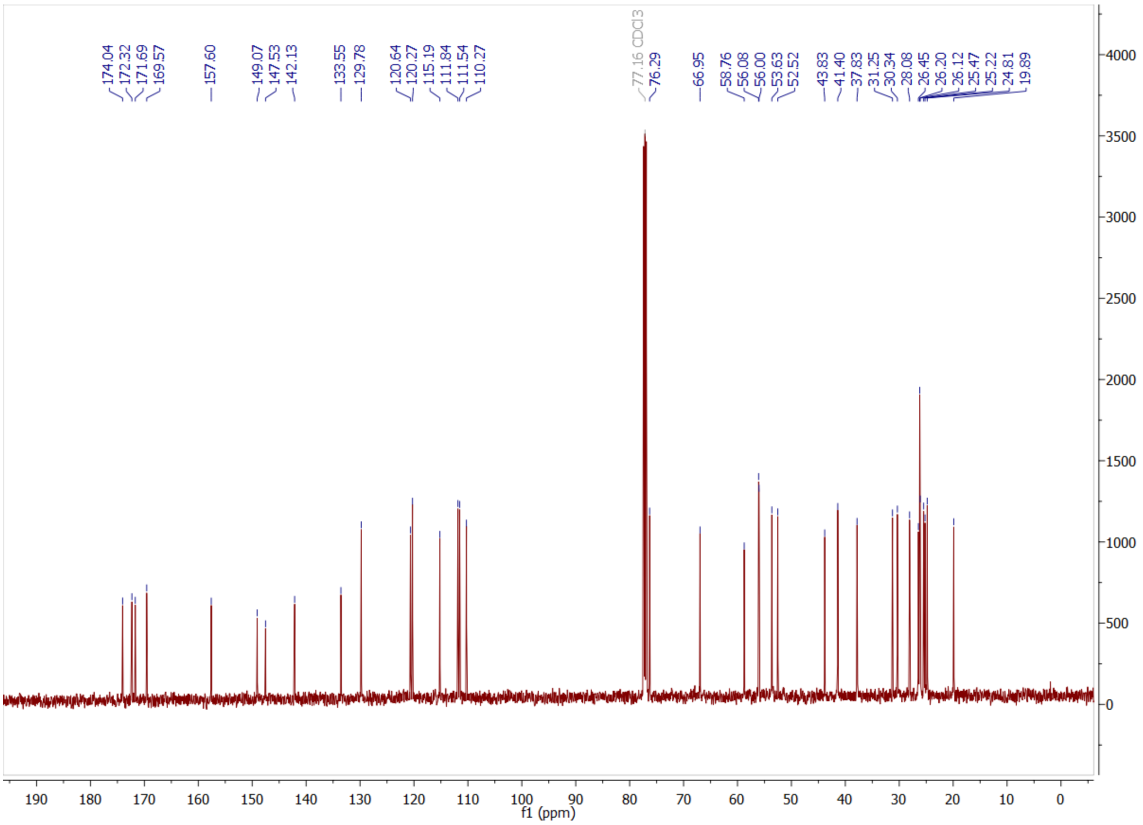

Figure S45. HPLC of compound **13c**.

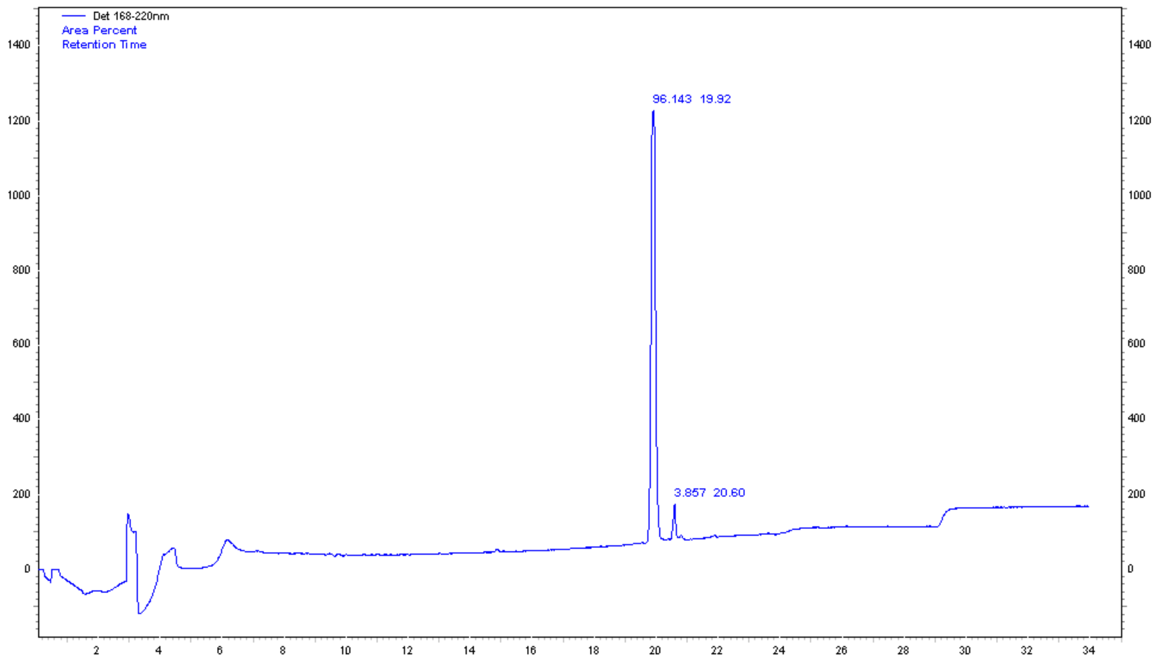

Figure S46. ^{1}H-NMR of compound **13d**.

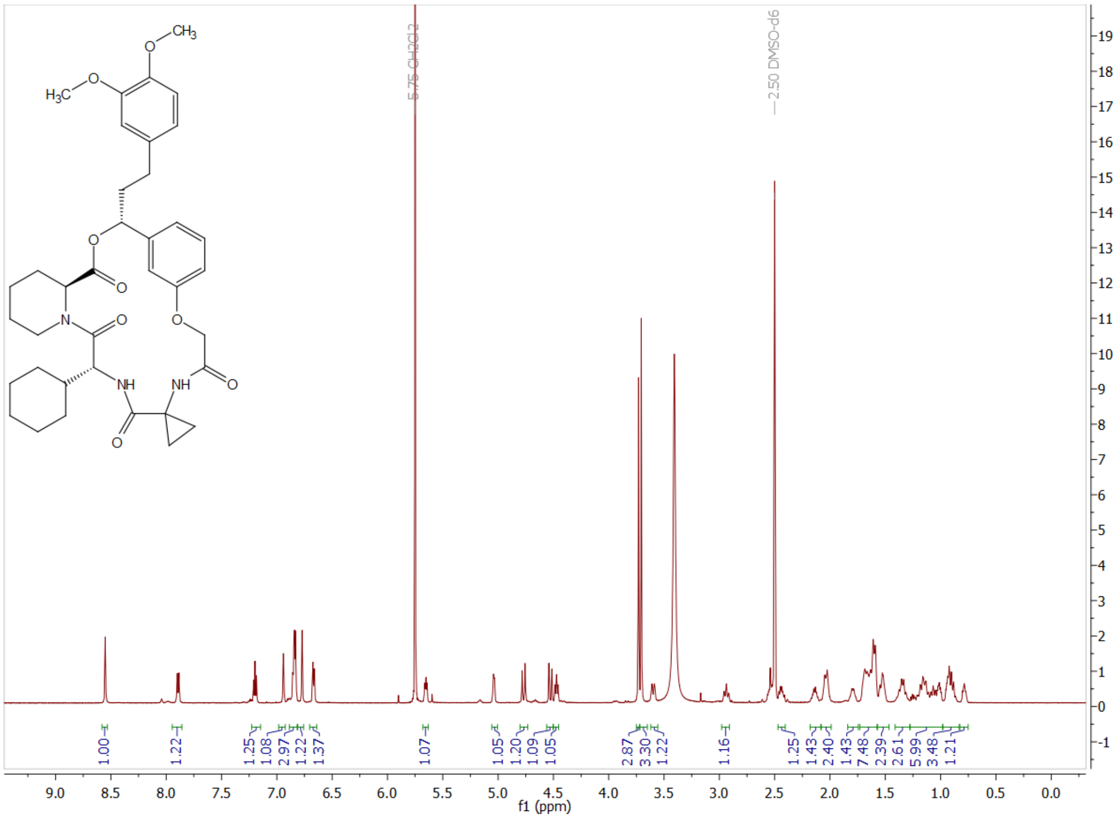

Figure S47. ^{13}C-NMR of compound **13d**.

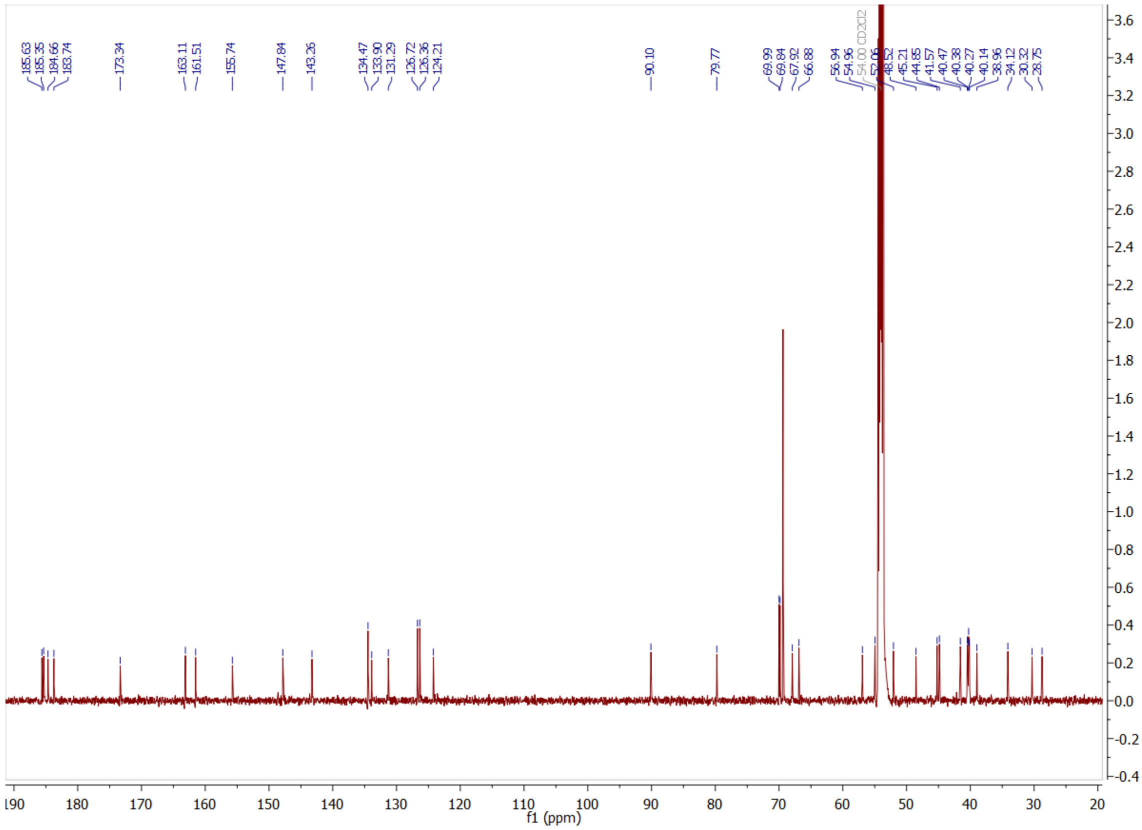

Figure S48. HPLC of compound **13d**.

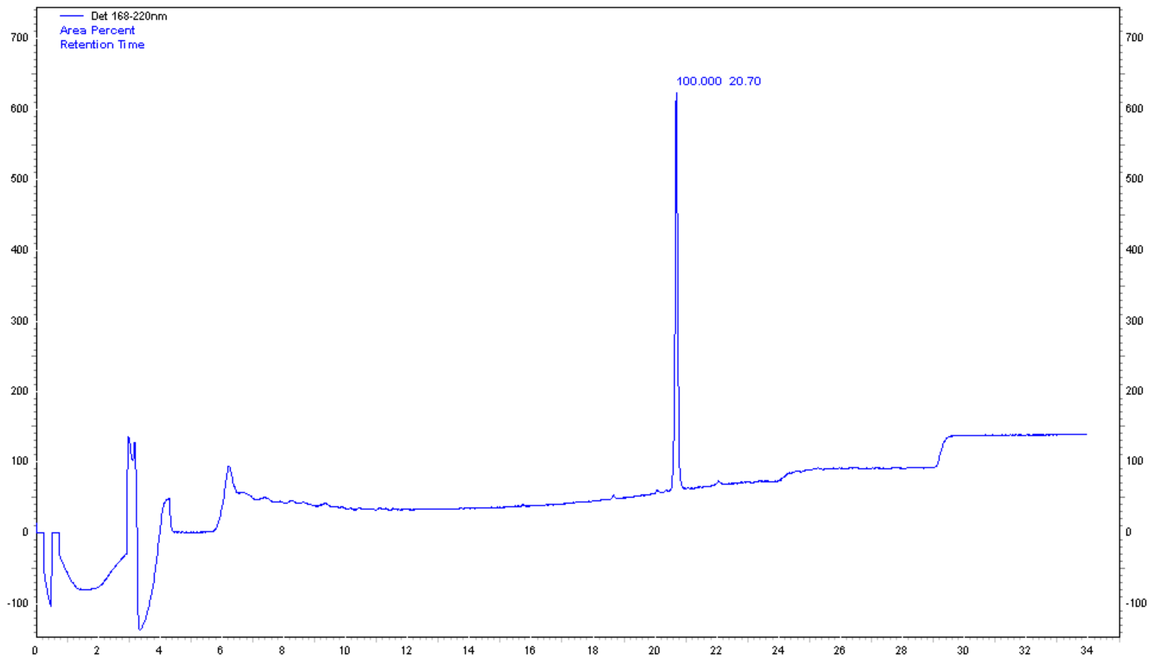

Figure S49. ^{1}H-NMR of compound **13e**.

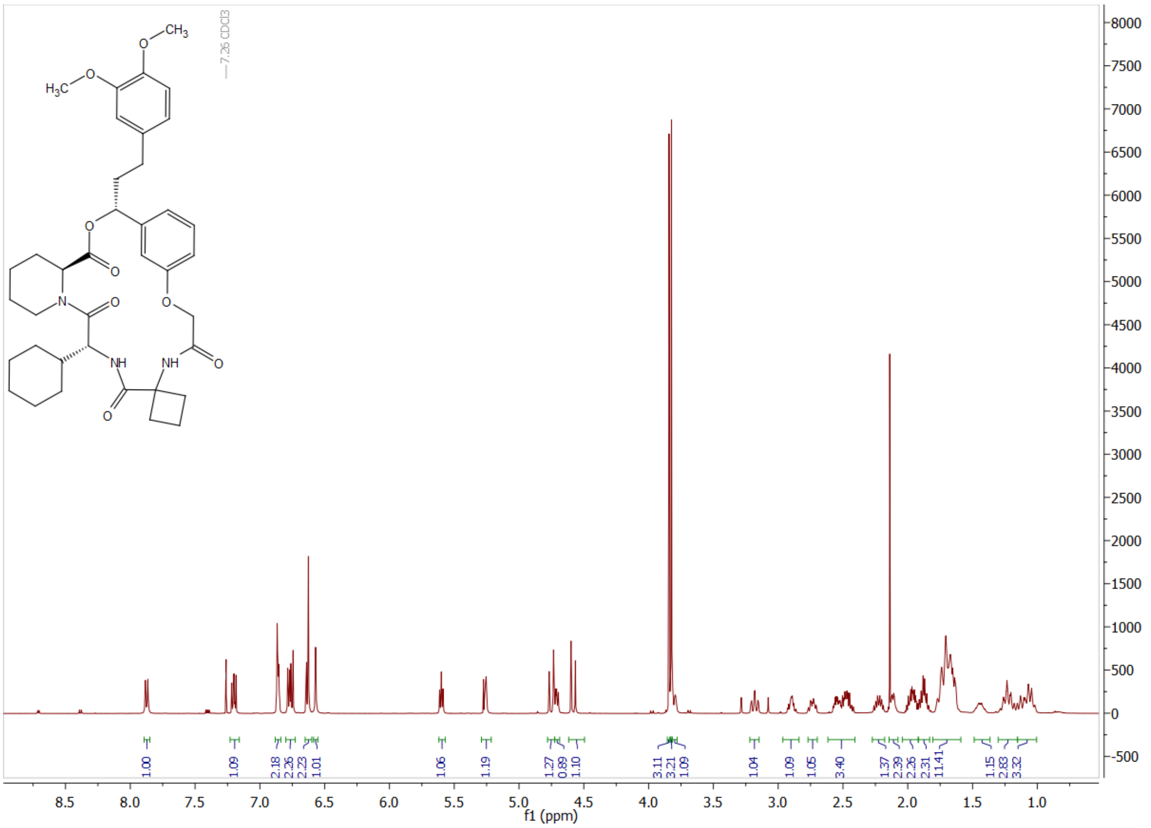

Figure S50. ^{13}C-NMR of compound **13e**.

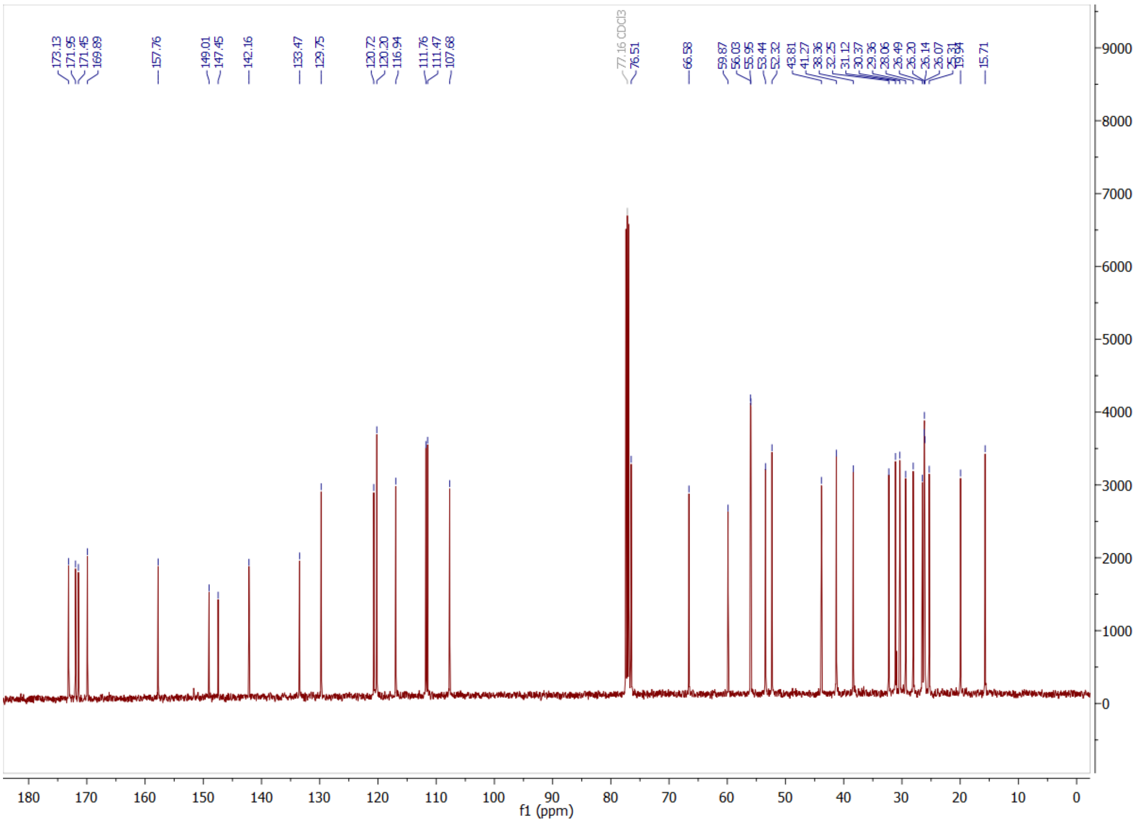

Figure S51. HPLC of compound **13e**.

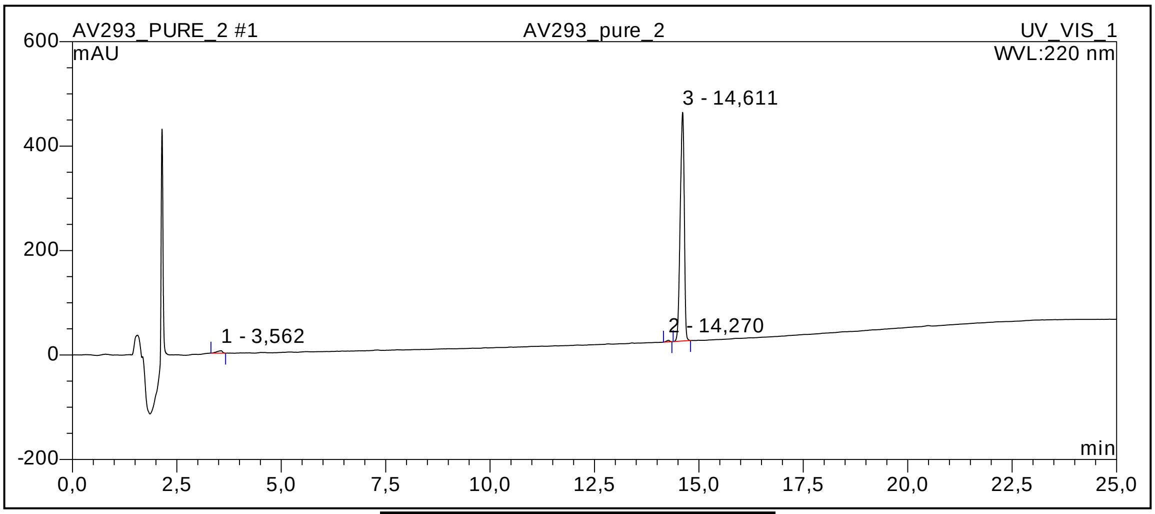

No.	Ret.Time min	Rel.Area %
1	3,56	1,47
2	14,27	0,53
3	14,61	98,00
Total		100

Figure S52. HPLC of compound **18f**.

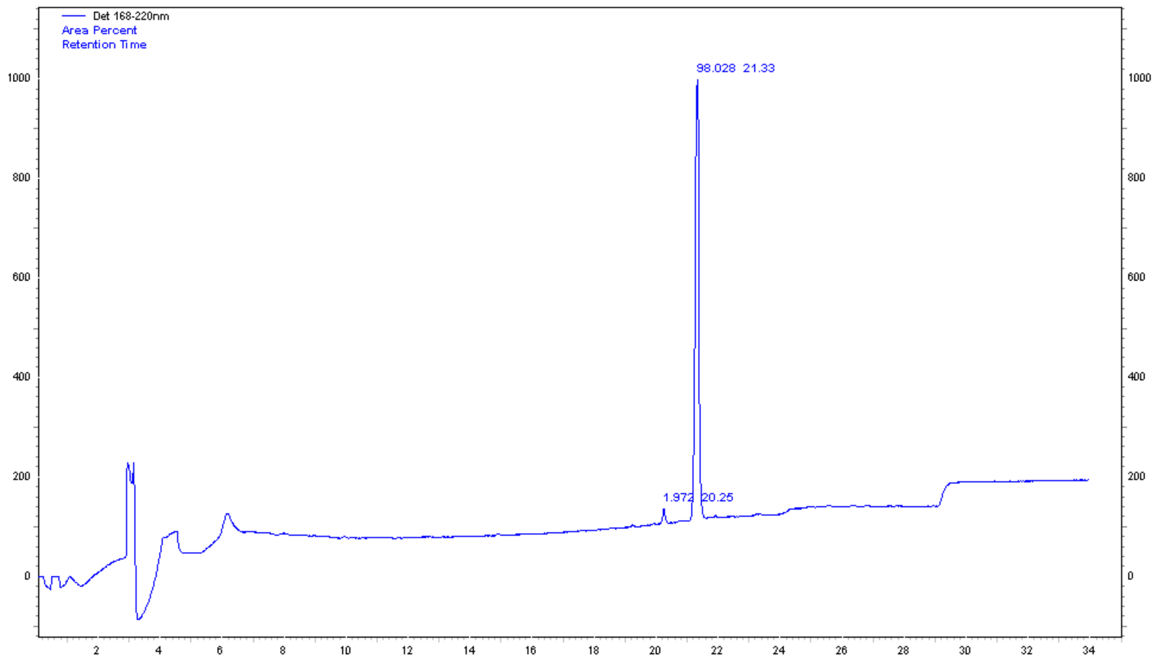

Figure S53. ^{1}H-NMR of compound **13g**.

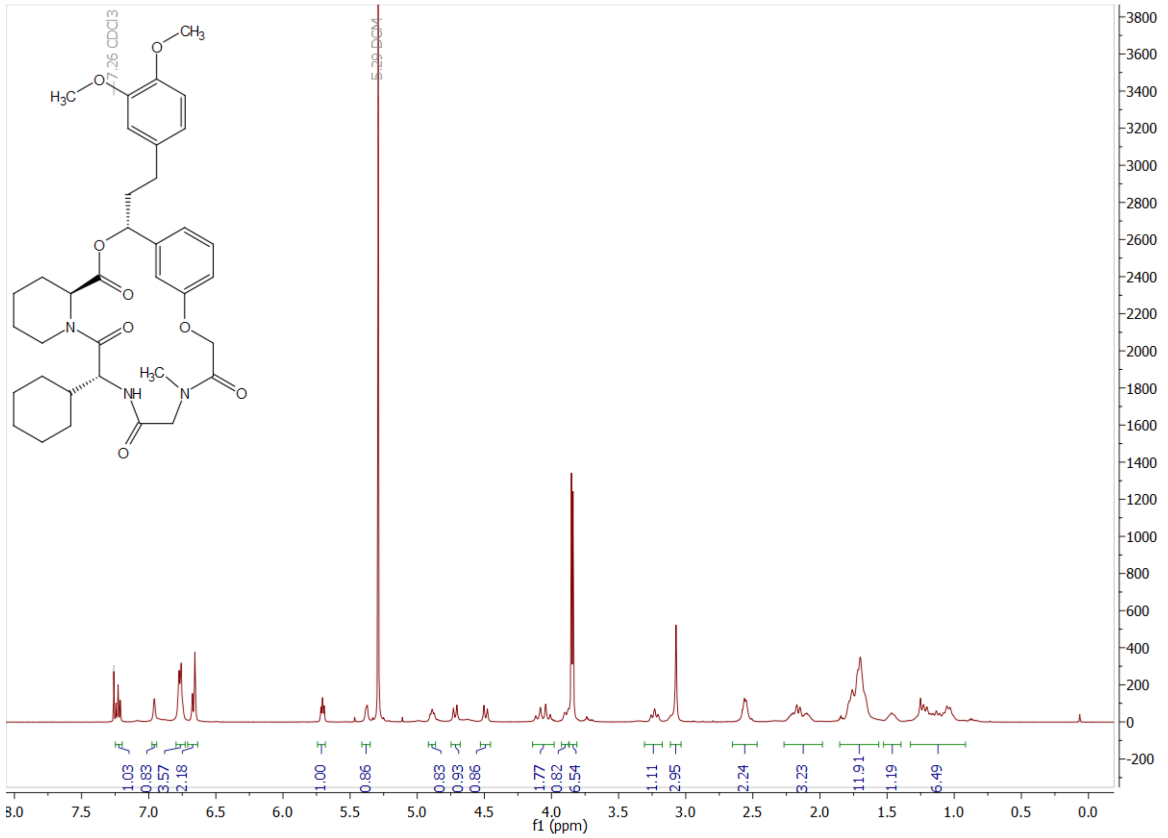

Figure S54. ^{13}C-NMR of compound **13g**.

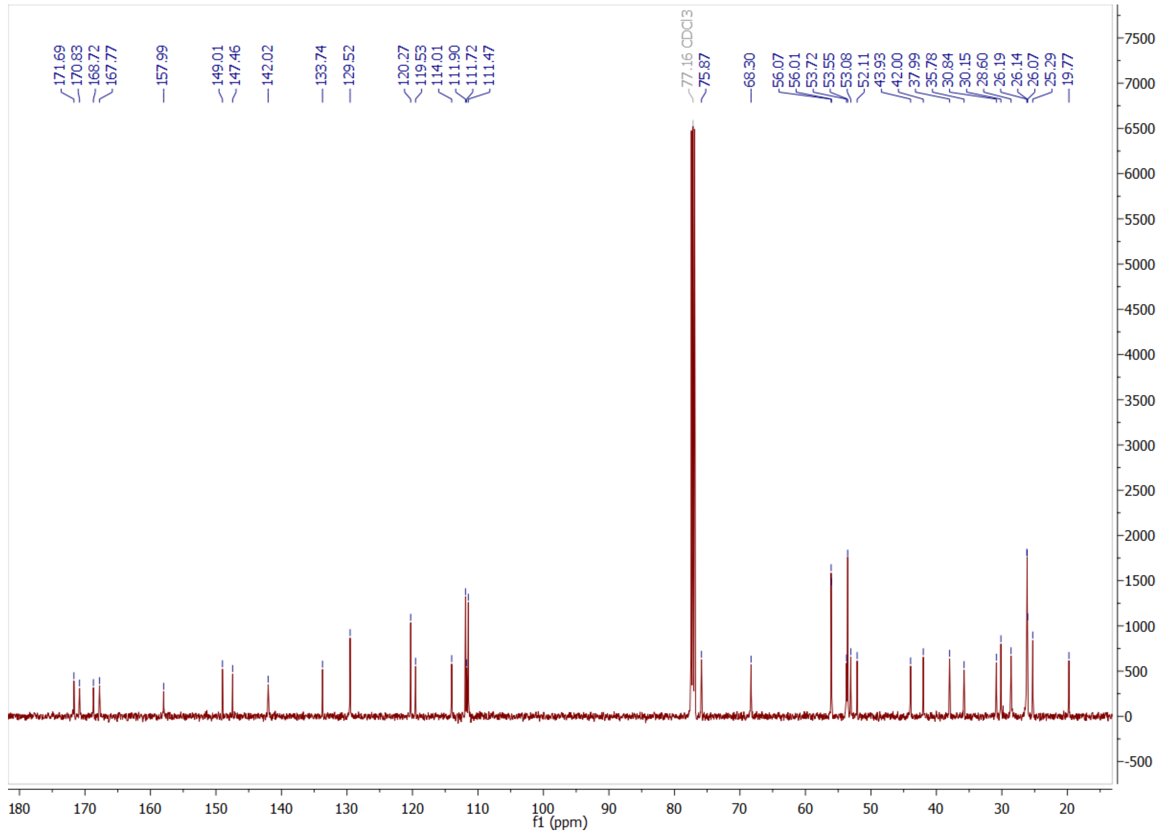

Figure S55. HPLC of compound **13g**.

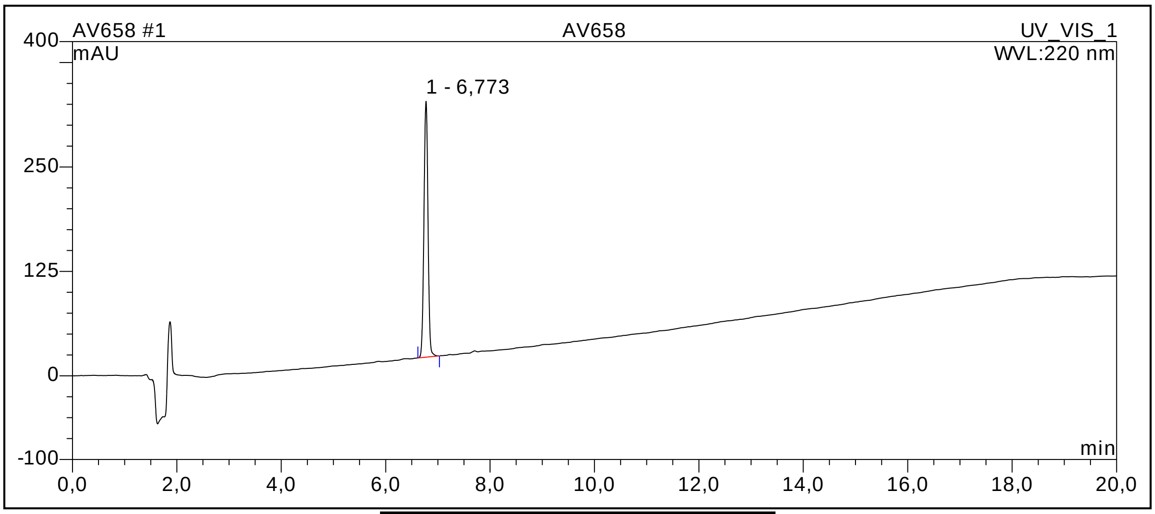

No.	Ret.Time min	Rel.Area %
1	6,77	100,00
Total		100

Figure S56. HPLC of compound **13i**.

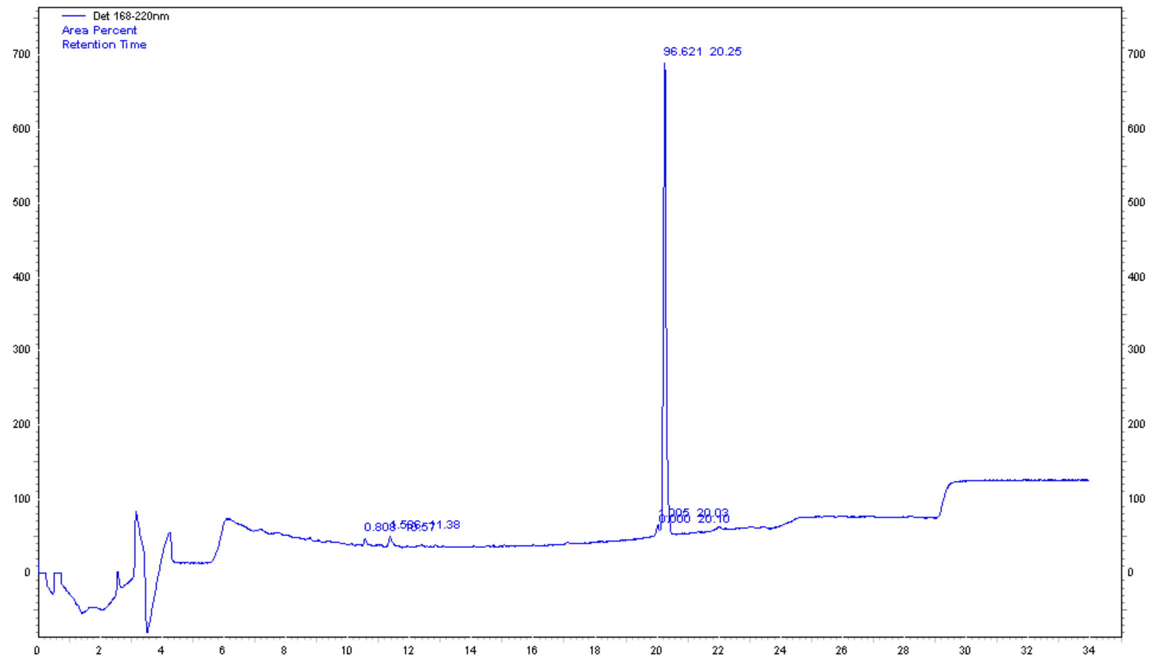

Figure S57. HPLC of compound **13j**.

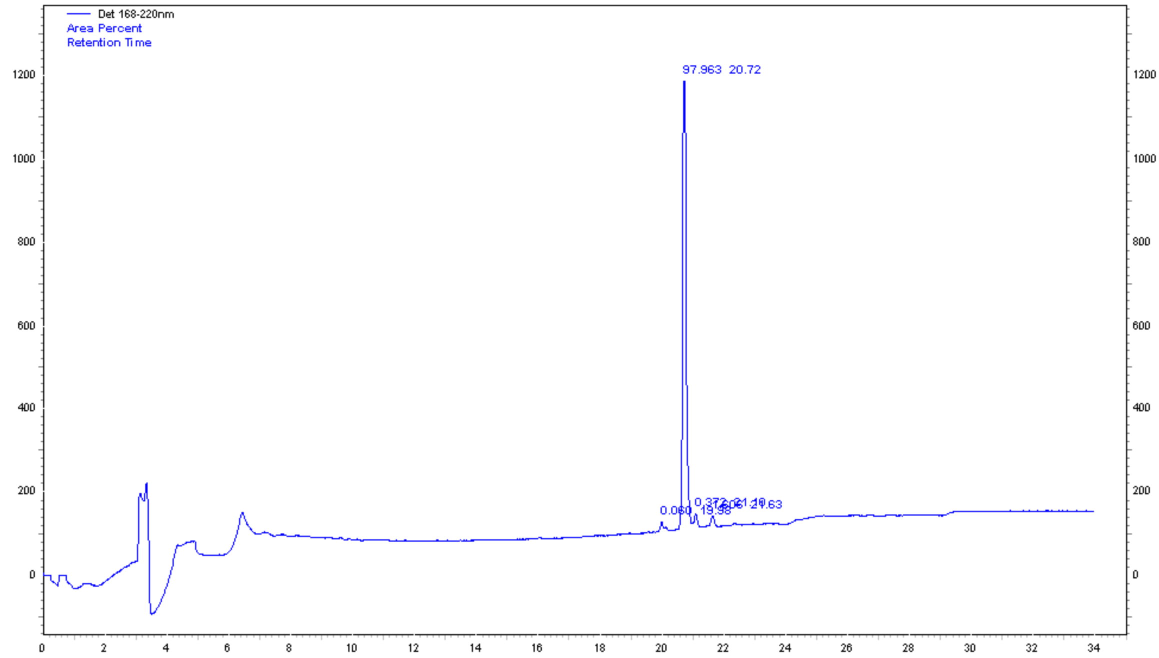

Figure S58. HPLC of compound **13k**.

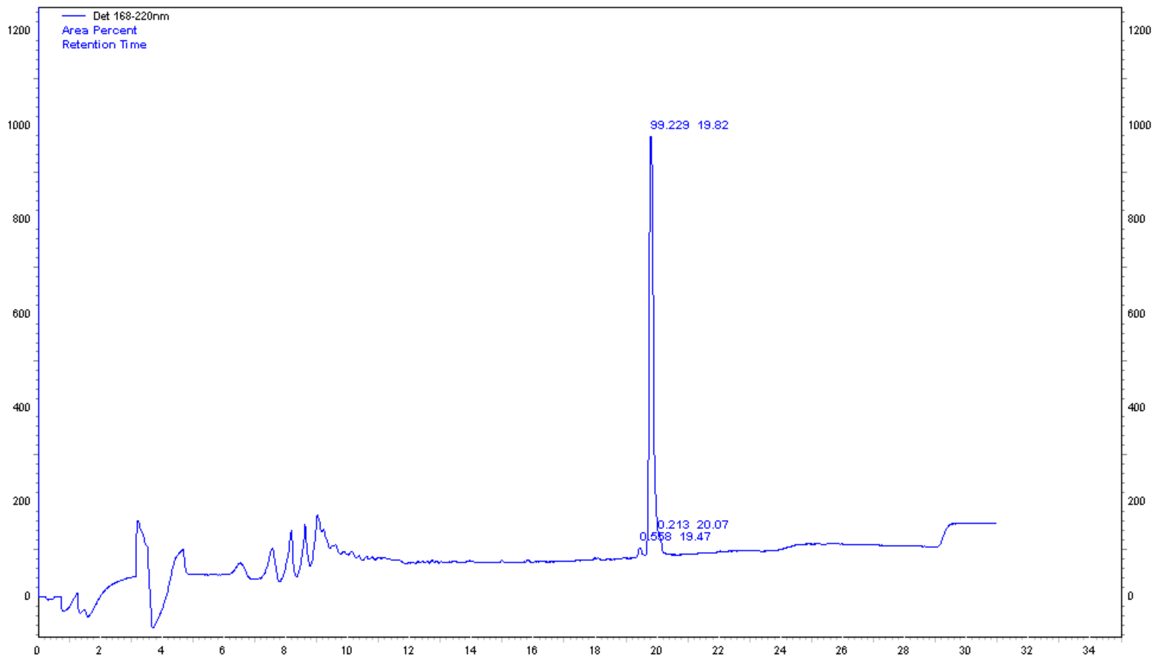

Figure S59. ^{1}H-NMR of compound **13l**.

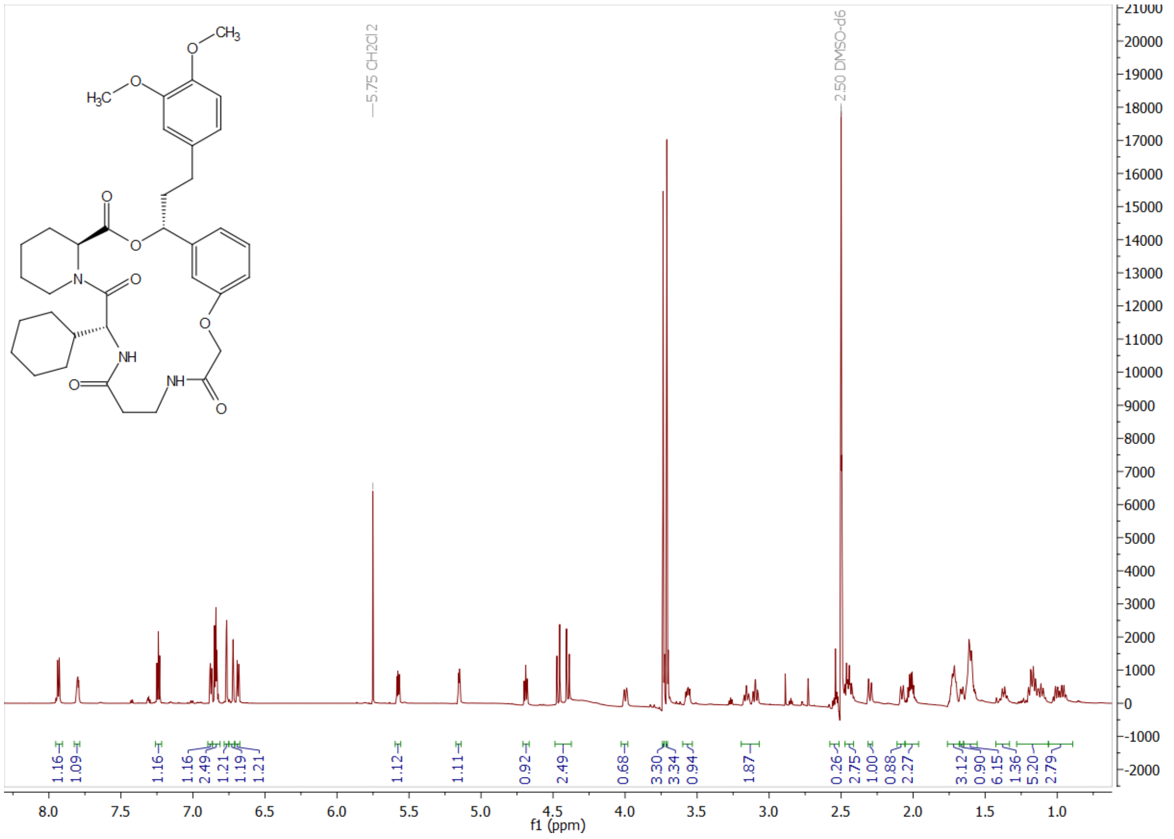

Figure S60. ^{13}C-NMR of compound **13l**.

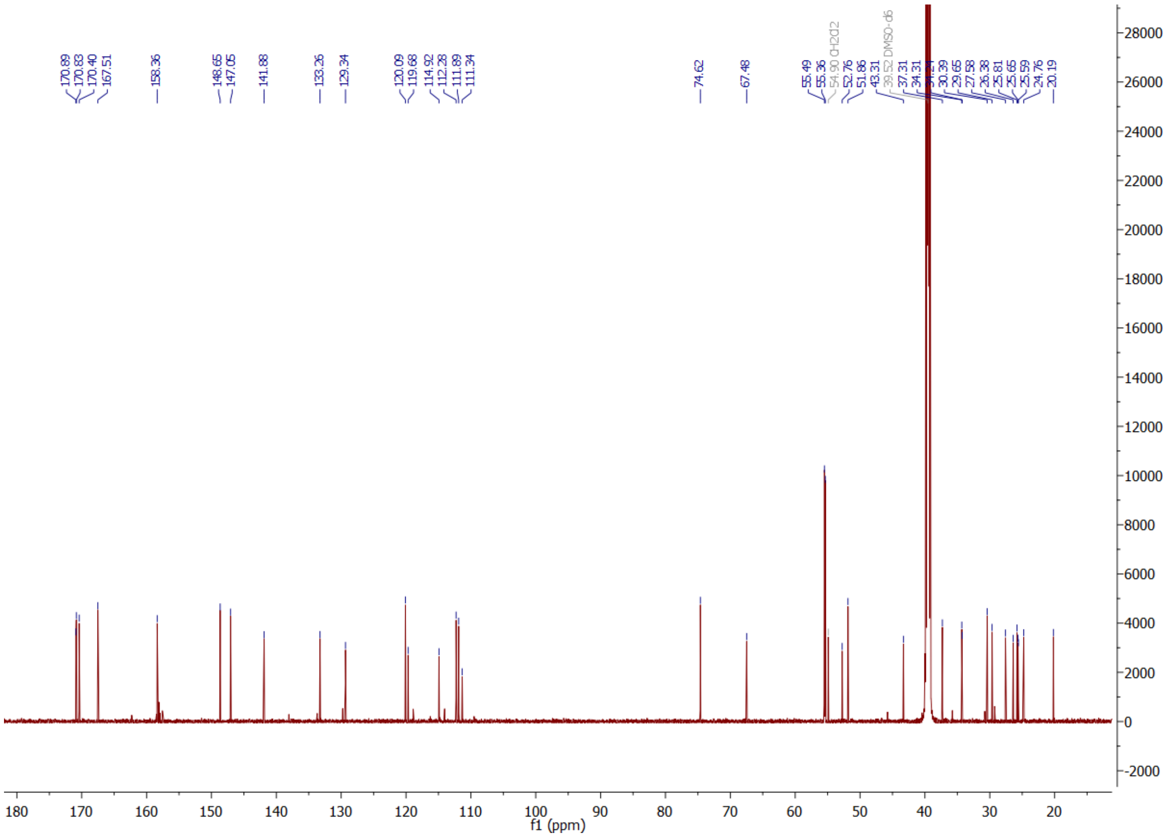

Figure S61. HPLC of compound **13l**.

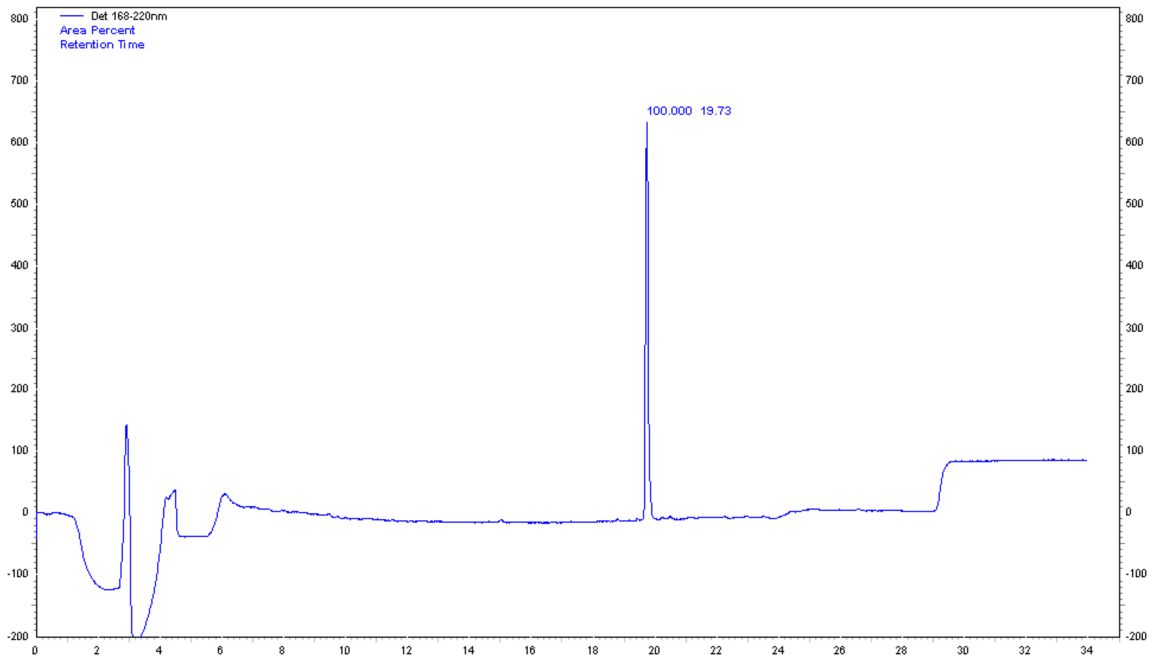

Figure S62. HPLC of compound **13m**.

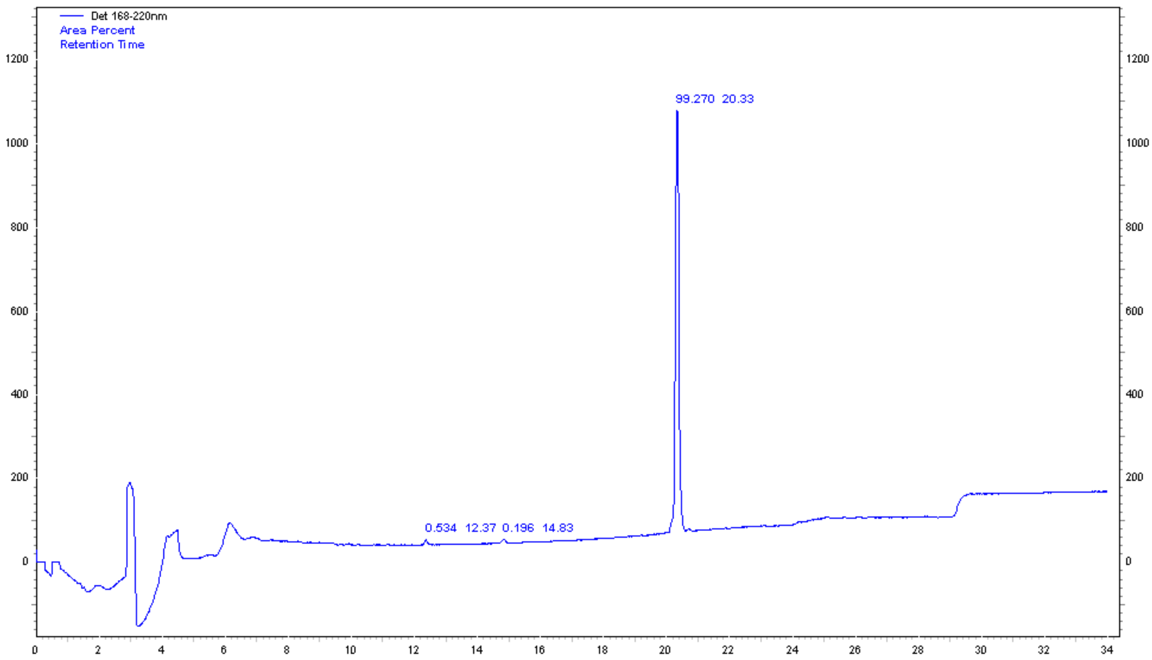

Figure S64. HPLC of compound **13n**.

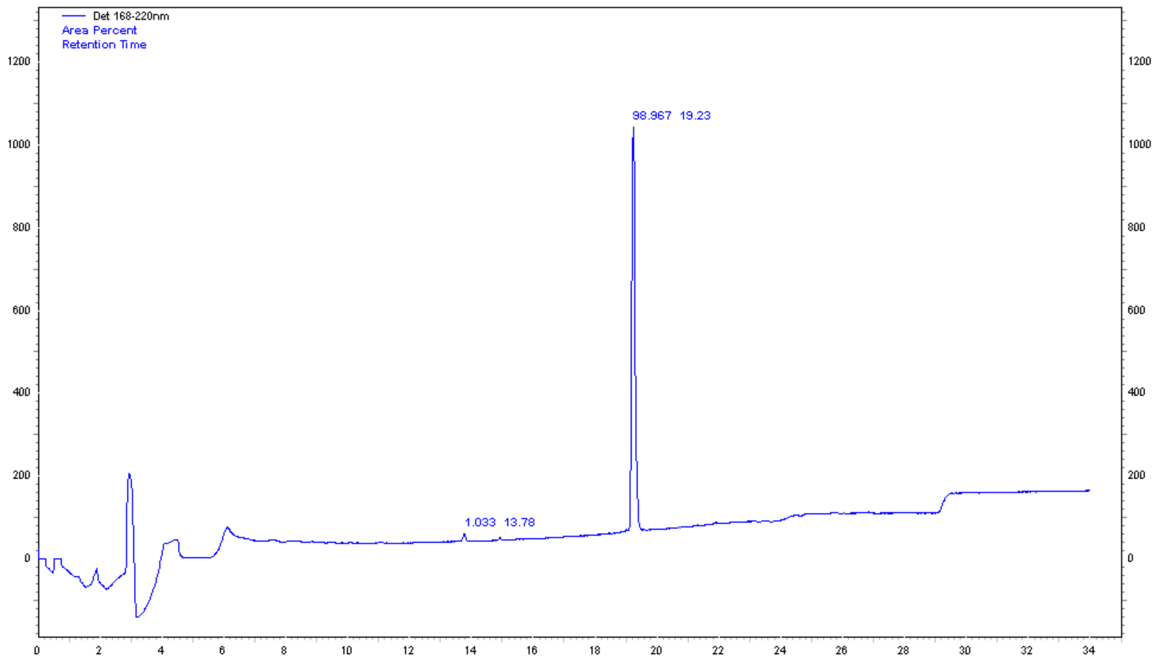

Figure S64. HPLC of compound **13o**.

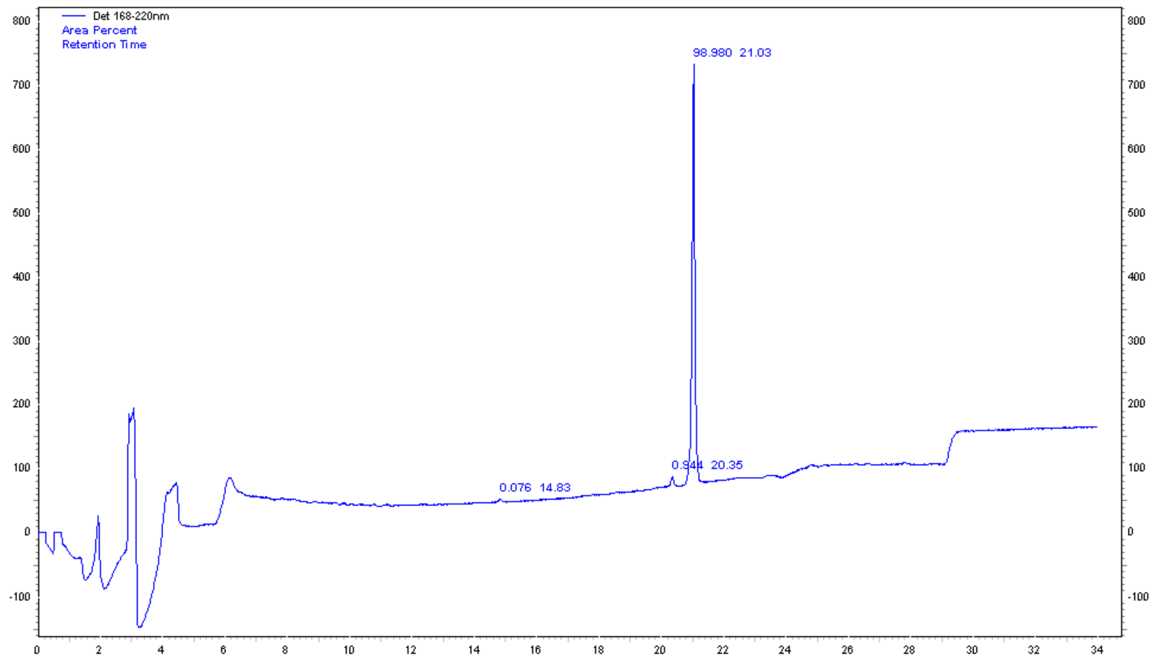

Figure S65. ^{1}H-NMR of compound **25**.

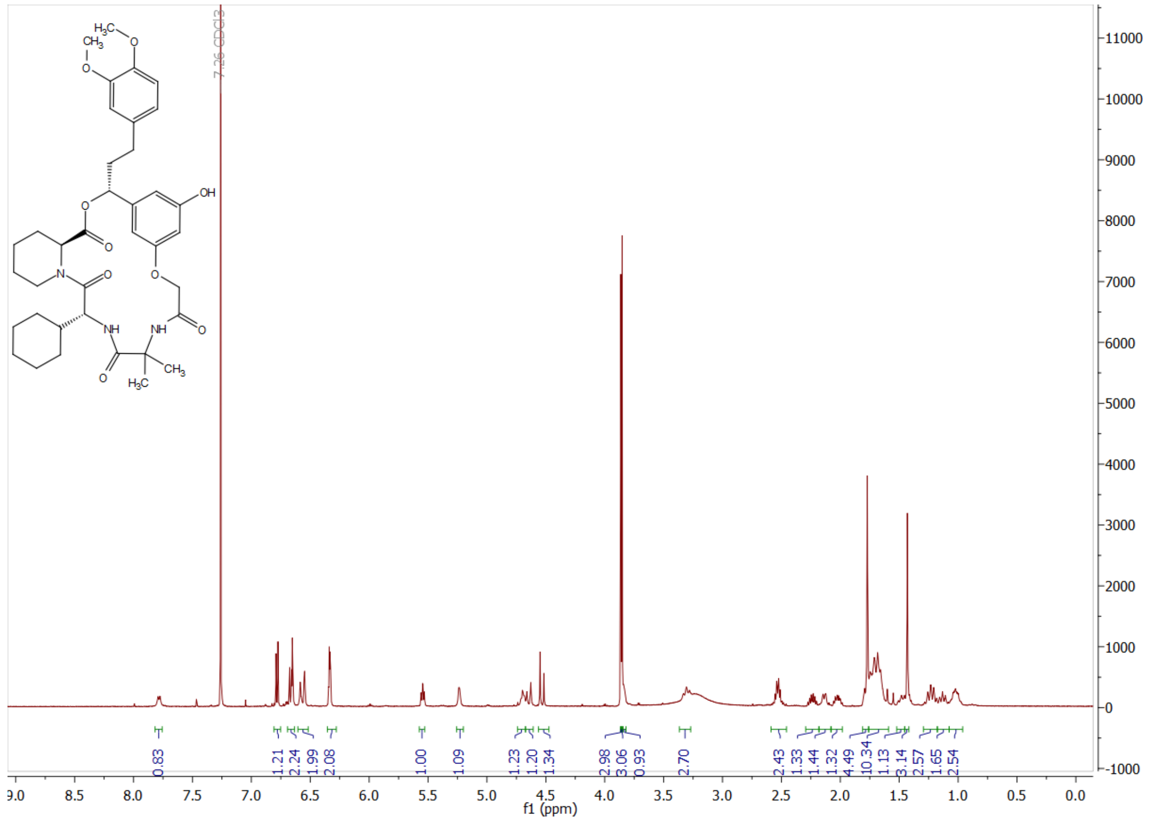

Figure S66. ^{13}C-NMR of compound **25**.

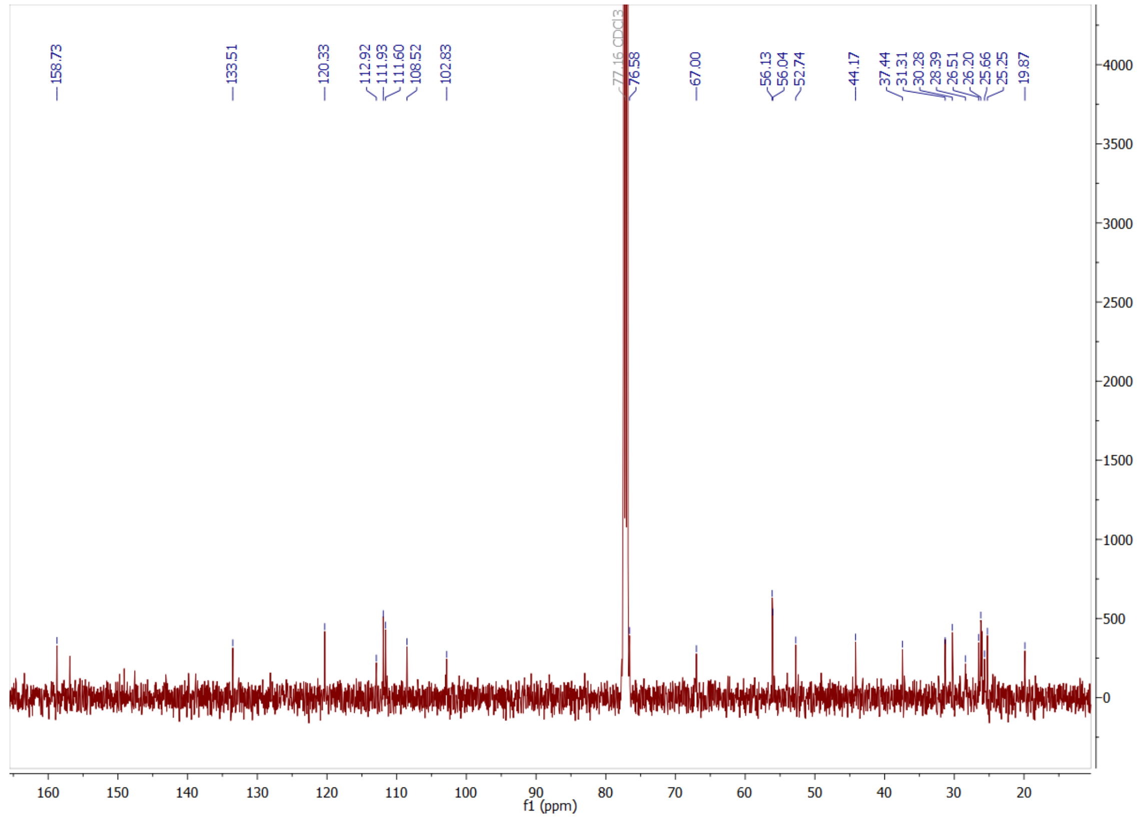

Figure S67. HPLC of compound **25**.

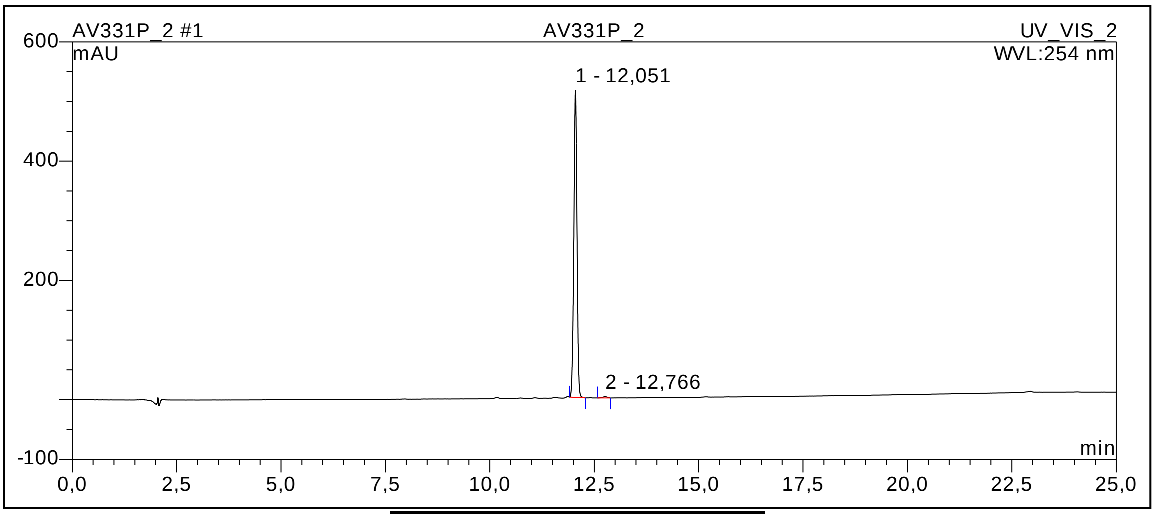

No.	Ret.Time min	Rel.Area %
1	12,05	99,40
2	12,77	0,60
Total		100

Figure S68. ^{1}H-NMR of compound **14a**.

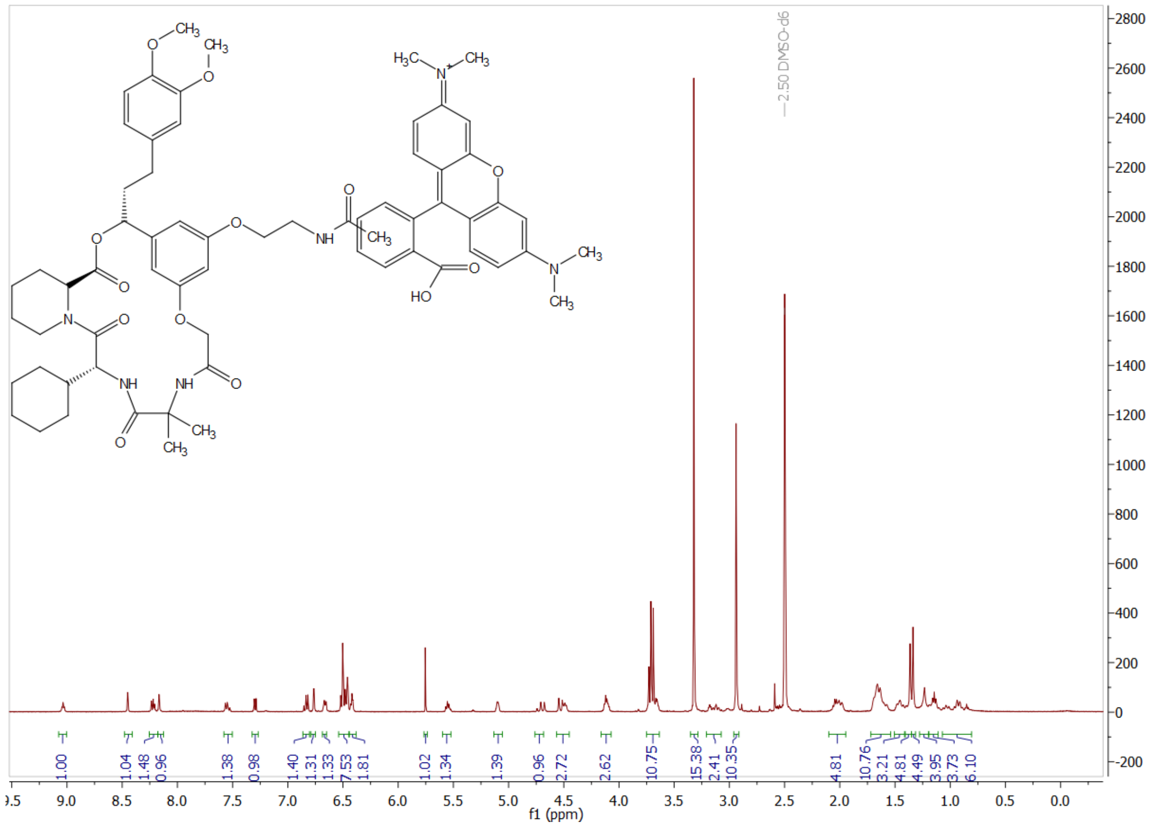

Figure S69. ^{13}C-NMR of compound **14a**.

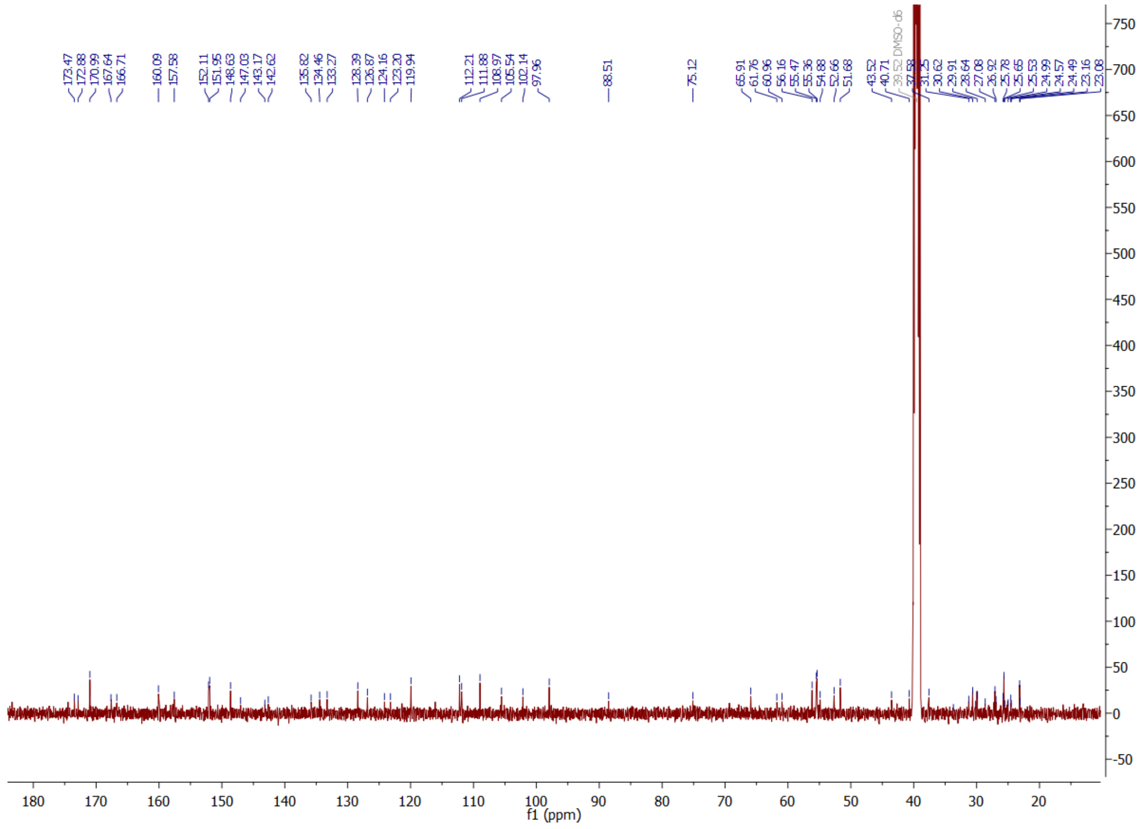

Figure S70. HPLC of compound **14a**.

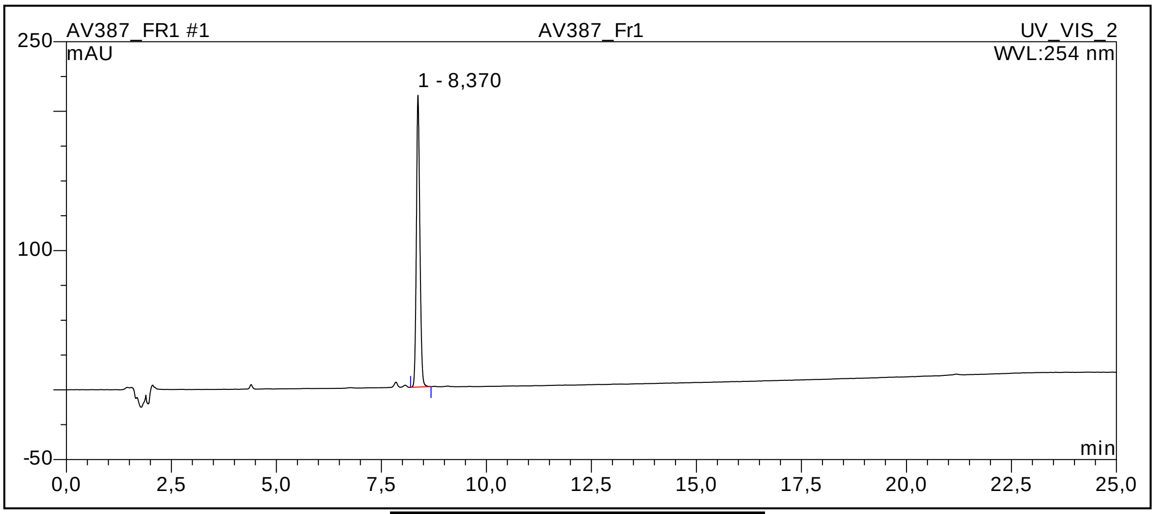

No.	Ret.Time min	Rel.Area %
1	8,37	100,00
Total		100

Figure S71. HPLC of compound **14b**.

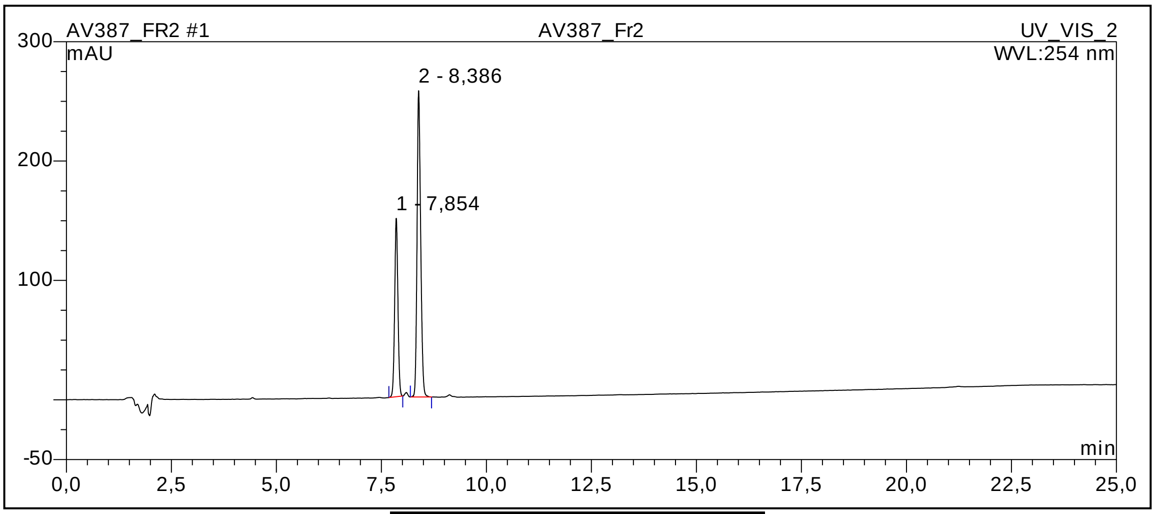

No.	Ret.Time min	Rel.Area %
1	7,85	34,29
2	8,39	65,71
Total		100

Figure S72. HPLC of compound **14c**.

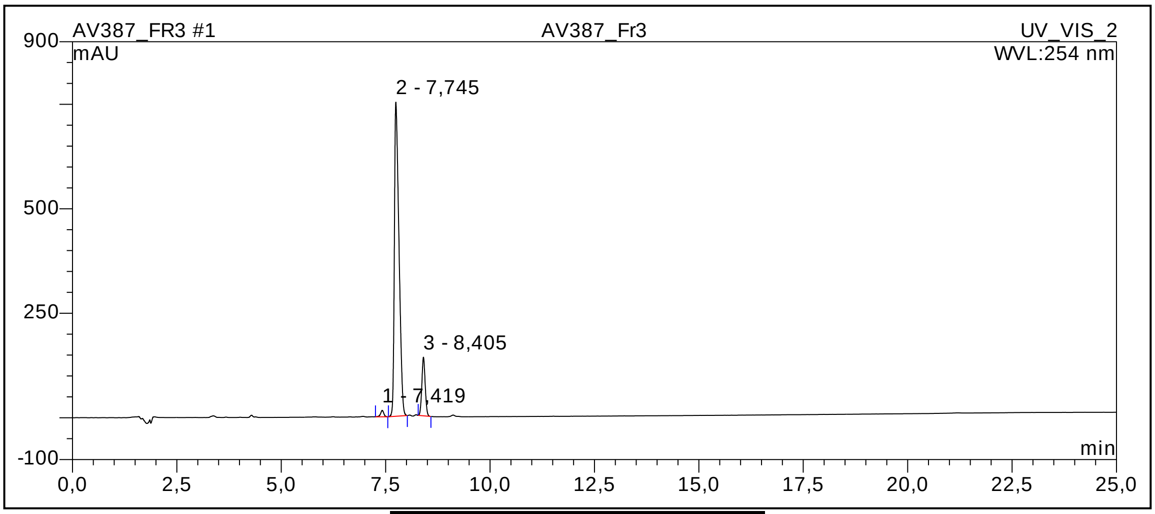

No.	Ret.Time min	Rel.Area %
1	7,42	1,24
2	7,75	86,98
3	8,40	11,78
Total		100

11. References

[1] W. Kabsch, *Acta Crystallogr D Biol Crystallogr* **2010**, *66*, 125-132.

[2] a) E. Potterton, P. Briggs, M. Turkenburg, E. Dodson, *Acta Crystallographica Section D* **2003**, *59*, 1131-1137; b) L. Potterton, J. Agirre, C. Ballard, K. Cowtan, E. Dodson, P. R. Evans, H. T. Jenkins, R. Keegan, E. Krissinel, K. Stevenson, A. Lebedev, S. J. McNicholas, R. A. Nicholls, M. Noble, N. S. Pannu, C. Roth, G. Sheldrick, P. Skubak, J. Turkenburg, V. Uski, F. von Delft, D. Waterman, K. Wilson, M. Winn, M. Wojdyr, *Acta Crystallogr D Struct Biol* **2018**, *74*, 68-84.

[3] a) P. R. Evans, *Acta Crystallogr D Biol Crystallogr* **2011**, *67*, 282-292; b) P. R. Evans, G. N. Murshudov, *Acta Crystallographica Section D-Biological Crystallography* **2013**, *69*, 1204-1214.

[4] a) A. A. Lebedev, A. A. Vagin, G. N. Murshudov, *Acta Crystallogr D* **2008**, *64*, 33-39; b) A. Vagin, A. Teplyakov, *Acta Crystallographica Section D-Biological Crystallography* **2010**, *66*, 22-25.

[5] L. B. Emsley P, Scott WG, Cowtan K, *Acta Crystallogr D Biol Crystallogr* **2010**, *66(Pt 4)*, 486-501.

[6] a) G. N. Murshudov, P. Skubak, A. A. Lebedev, N. S. Pannu, R. A. Steiner, R. A. Nicholls, M. D. Winn, F. Long, A. A. Vagin, *Acta Crystallographica Section D* **2011**, *67*, 355-367; b) S. R. Vagin AA, Lebedev AA, Potterton L, McNicholas S, Long F, Murshudov GN, *Acta Crystallogr D Biol Crystallogr* **2004**, *60(Pt 12 Pt 1)*, 2184-2195; c) L. F. Nicholls RA, Murshudov GN, *Acta Crystallogr D Biol Crystallogr* **2012**, *68(Pt 4)*, 404-417; d) V. A. Murshudov GN, Dodson EJ, *Acta Crystallogr D Biol Crystallogr* **1997**, *53(Pt 3)*, 240-255; e) M. G. Winn MD, Papiz MZ, *Methods Enzymol* **2003**, *374*, 300-321.

[7] D. M. F. van Aalten, R. Bywater, J. B. C. Findlay, M. Hendlich, R. W. W. Hooft, G. Vriend, *Journal of Computer-Aided Molecular Design* **1996**, *10*, 255-262.

[8] S. Gaali, A. Kirschner, S. Cuboni, J. Hartmann, C. Kozany, G. Balsevich, C. Namendorf, P. Fernandez-Vizarra, C. Sippel, A. S. Zannas, R. Draenert, E. B. Binder, O. F. X. Almeida, G. Rühter, M. Uhr, M. V. Schmidt, C. Touma, A. Bracher, F. Hausch, *Nat Chem Biol* **2015**, *11*, 33-37.

[9] X. Feng, C. Sippel, A. Bracher, F. Hausch, *J Med Chem* **2015**, *58*, 7796-7806.

[10] J. Chatterjee, B. Laufer, H. Kessler, *Nat Protoc* **2012**, *7*, 432-444.

[11] R. Wawrzinek, J. Ziomkowska, J. Heuveling, M. Mertens, A. Herrmann, E. Schneider, P. Wessig, *Chemistry* **2013**,19, 17349-57.

12. Author contributions

Andreas M. Voll: design and synthesis, writing of original draft, crystallography, FP-assay evaluation (lead)

Dr. Christian Meyners: FP, FRET, ITC, and FLT assays and evaluation of the tracer compound

Martha C. Taubert: Cellular assays – Jurkat cells

Thomas Bajaj: Cellular assays – SIM-A9 cells

Thomas M. Geiger: Cellular assays – NanoBRET assays

Anna Charalampidou: generation of mono-Cys variants, protein labelling and FLT assays and (supporting)

Tim Heymann, Stephanie Merz, Patrick L. Purder: FP-assay (supporting)

Dr. Andreas Bracher: crystallography (supporting)

Dr. Nils C. Gassen: supervision cellular assays SIM-A9 (supporting)

Jürgen Kolos, Prof. Dr. Pablo Wessig: contribution of essential reagents (supporting)

Prof. Dr. Felix Hausch: conception of the project, project administration, funding acquisition, writing of the manuscript (PI, research supervisor)
